# Targeting NOTCH1-KEAP1 axis retards chronic liver injury and liver cancer progression via regulating stabilization of NRF2

**DOI:** 10.1186/s13046-025-03488-3

**Published:** 2025-08-09

**Authors:** Chunxiao Chang, Meng Wang, Jia Li, Sihao Qi, Xiaojuan Yu, Jun Xu, Shengbin Shi

**Affiliations:** 1 Department of Gastrointestinal Oncology, Shandong Cancer Hospital and Institute, Shandong First Medical University, Shandong Academy of Medical Sciences, Jinan City, 250117 Shandong Province China; 2 Department of Oncology, Shandong Jining First People’s Hospital, Jining City, 272111 Shandong Province China; 3 Department of Abdominal Radiotherapy, Shandong Cancer Hospital and Institute, Shandong First Medical University, Shandong Academy of Medical Sciences, Jinan City, 250117 Shandong Province China; 4 Department of Clinical Research, Shandong Cancer Hospital and Institute, Shandong First Medical University, Shandong Academy of Medical Sciences, Jinan City, 250117 Shandong Province China

**Keywords:** NOTCH1, Hepatocellular carcinoma (HCC), Chronic liver inflammation, Nrf2, Ferroptosis

## Abstract

**Background:**

Chronic liver injury is a key factor in diseases like hepatocellular carcinoma (HCC), steatohepatitis (NASH), and viral hepatitis type B and C (HBV, HCV). Understanding its molecular mechanisms is crucial for effective treatment. The NOTCH1 signaling pathway, though not fully understood, is implicated in liver injury and may be a potential therapeutic target.

**Methods:**

Clinical HCC, HBV, HCV and NASH samples and additional in vitro and in vivo performances were subjected to confirm the role of NOTCH1 and its downstream targets via a series of biochemical assays, molecular analysis approaches and targeted signaling pathway assay, etc.

**Results:**

The present study first verified the abnormal elevation of NOTCH1 in hepatocytes from patients with steatohepatitis, HCC, HBV, HCV, and mouse models. Crucially, we discovered that hepatocyte-specific *NOTCH1* knockout reduces hepatocellular damage in chronic liver inflammation and HCC mouse models, whereas adeno-associated virus serotype 8 (AAV8)-mediated NOTCH1 overexpression in hepatocytes exacerbates liver injury-related phenotype on-setting. Mechanistically, we showed that NOTCH1 has a new role in controlling ferroptosis and oxidative damage in hepatocytes. It interacts with Kelch-like ECH-associated protein 1 (KEAP1) and is directly recruited through its intracellular domain (NICD1). Additionally, the KEAP1 recruited by NOTCH1 impeded the binding stability of KEAP1-NFE2 like BZIP transcription factor 2 (Nrf2), promote the separation of KEAP1 and Nrf2, thereby reducing the stability of Nrf2 and hindering the ubiquitination-related proteasome degradation of Nrf2. Crucially, we also discovered that NOTCH1’s ANK domain is essential for NICD1-KEAP1 contacts and signaling activation. The inability of NOTCH1 with ANK domain mutants (ΔANK) to connect with KEAP1 and increase its expression emphasizes the importance of the ANK domain in KEAP1-NRF2 signaling. By reversing the downregulation of KEAP1 and the overexpression of NRF2, ANK function is linked to ferroptosis and ROS buildup. ANK domain targeting may slow the course of HCC and liver damage.

**Conclusions:**

Targeting the NOTCH1-KEAP1-NRF2 axis as a possible chronic hepatic injury therapy is supported by these findings, which identify NOTCH1-KEAP1 as an NRF2 suppressor that accelerates the progression of liver injury.

**Supplementary Information:**

The online version contains supplementary material available at 10.1186/s13046-025-03488-3.

## Introduction

Chronic hepatic inflammation, a persistent inflammatory condition of the liver, remains a significant global health burden, often progressing to severe complications such as cirrhosis and hepatocellular carcinoma (HCC) [1]. According to the World Health Organization (WHO), chronic hepatitis B and C infections are the leading causes of HCC, accounting for approximately 80% of cases worldwide [1, 2]. The pathogenesis of HCC in chronic liver inflammation involves a complex interplay of viral persistence, immune-mediated liver injury, and dysregulated cellular processes. Of note, liver injury brought on by both acute and chronic liver illnesses, such as alcoholic liver diseases, hepatitis B and C, and nonalcoholic steatohepatitis, results in inflammation of the liver [2, 3]. Numerous cell types, such as natural killer cells, natural killer T cells, T cells, dendritic cells, and macrophages, are drawn in and activated during liver inflammation [4]. In particular, hepatocytes, the primary functional cells of the liver, play a central role in this progression [4–6]. They are not only the primary targets of viral infection but also key mediators of the inflammatory and fibrotic responses that drive carcinogenesis [6–8]. Studies have shown that hepatocyte-derived signaling pathways, including the Wnt/β-catenin, PI3K/AKT, and JAK/STAT cascades, are critically involved in the transformation of normal hepatocytes into malignant cells [9, 10]. Furthermore, hepatocyte apoptosis, senescence, and metabolic reprogramming contribute to the tumor microenvironment, fostering HCC development [11, 12]. Understanding the molecular mechanisms by which hepatocytes influence chronic hepatitis and its progression to HCC is essential for developing targeted therapeutic strategies.

Over the course of liver inflammation development and HCC progression, the NOTCH signaling pathway is an evolutionarily conserved mechanism and plays a crucial role in embryonic development, tissue homeostasis, and the pathogenesis of various diseases, such as cardiovascular disease, chronic metabolic disease, and cancer [13–15]. The NOTCH signaling pathway consists of four membrane receptors (NOTCH 1–4) and five ligands (Delta-like 1, 3, and 4; Jagged 1 and 2). The interaction between a NOTCH ligand from a donor cell and the NOTCH receptor on a receiving cell leads to the cleavage of the NOTCH receptor by ADAM and γ-secretase, resulting in the release of the NOTCH intracellular domain (NICD) [16]. Once released, NICD1 forms a ternary complex to activate the expression of target genes such as HES1 and then induce the occurrence of related diseases [16, 17]. Among NOTCH signaling components, NOTCH1 plays an essential role in mediating the progression of metabolic disorders via regulating numerous biological processes, such as oxidative stress, inflammatory response, fibrosis and apoptosis [18, 19]. Previous reports demonstrated that NOTCH1 loss ameliorates lipid accumulation in liver through inducing fatty acid oxidation to improve fatty liver [20]. In steatohepatitis, NOTCH1 aberrant expression is associated with inhibition of hepatic fibrosis in hepatic stellate cells (HSCs) [20, 21]. NOTCH1 activity is unnoticeable in the liver from healthy adults, but higher activation of NOTCH signaling is detected in hepatocytes of patients with fatty liver [14, 20]. NOTCH1 activation in HSCs contributes to collagen production mediated by advanced osteopontin (OPN) [20, 22]. Additionally, conditional deletion of NOTCH1 in HSCs exerts marked amelioration of steatohepatitis and liver fibrosis in vivo and in vitro through suppressing TLR4-mediated hyperactivation of HSC cells [22]. Thus, these studies revealed that NOTCH1 participates in the pathogenesis of liver injury-related diseases, but the mechanism is still not fully understood. However, there have been relatively few studies of NOTCH1-targeting drugs, and their development and test in liver diseases and metabolic syndrome requires further study.

Our study reveals that disrupting NOTCH1 alleviates liver inflammation, fibrosis, and HCC by reducing inflammatory and fibrogenic cytokines. We uncovered a novel role of NOTCH1 in regulating oxidative injury and ferroptosis in hepatocytes through its intracellular domain (NICD1), which recruits and interacts with KEAP1 to promote proteasome-dependent Nrf2 polyubiquitination and degradation. NOTCH1 drives chronic liver inflammation and cancer via KEAP1, identifying the NOTCH1-KEAP1 axis as an Nrf2 suppressor that exacerbates hepatitis and HCC progression. These findings highlight the NOTCH1-KEAP1 axis as a promising therapeutic target for liver injury.

## Materials and methods

### Reagents and antibodies

The protein synthesis inhibitor cycloheximide (CHX, #R750107, Sigma-Aldrich, USA), the proteasome inhibitor MG132 (#HY13259, MedChemExpress, USA) and the lysosome inhibitor chloroquine (CHO, #C6628, Sigma-Aldrich, USA) were added to the cell medium to explore the mechanism underlying Nrf2 degradation. BCA protein assay kit (#P0009, Beyotime Biotechnology, Shanghai, China) was used to examine protein concentrations. The main antibodies employed in this study, targeting the indicated proteins, were procured from Abcam Inc.: anti-GAPDH (#ab9485; 1/1500 dilutions), anti-NF-κB/p65 (#ab32536; 1/1000 dilutions), anti-phosphorylated NF-κB/p65 (#ab76302; 1/1000 dilutions), anti-4-HNE (#ab48506; 1/200 dilutions), and anti-F4/80 (#ab16911; 1/150 dilutions). Antibodies against NOTCH1 (#MA5-11961 and #MA5-32080, 1/150-1/1000 dilution), SLC7A11 (#PA1-16893; 1/1000 dilutions), anti-Nrf2 (#PA5-27882; 1/1000 dilutions), anti-KEAP1 (#PA5-99434; 1/150-1/1000 dilutions), and α-SMA (#14-9760-82; 1/100 dilutions) were obtained from Thermo Fisher Scientific (Shanghai, China). Furthermore, antibodies against Hes1 (#sc-166410; 1/100-1/500 dilutions) and anti-ACSL4 (#sc-271800; 1/500 dilutions) were obtained from Santa Cruz Biotechnology (USA). NOTCH1 intracellular domain (NICD1) (#4147; 1/1000 dilutions) was purchased from Cell Signaling Technology, Inc (Shanghai, China). Antibodies against anti-Ub (#GB115700; 1/1000 dilutions), anti-COL1A1 (#GB11022; 1/200 dilutions), and anti-GPX4 (#GB124327; 1/100-1/1000 dilutions) were obtained from Servicebio (Wuhan, China). The antibodies against anti-Flag (#14793, 1/1000 dilutions, CST), anti-HA (#ab9110, 1/1000 dilutions, Abcam), and anti-His (#D291-3; 1/1000 dilutions, MBL Beijing Biotech, Beijing, China) were also used in the study. The secondary antibodies used in this study for western blotting or immunohistochemical assays were Rabbit Anti-Mouse IgG (H&L) (#ab6728) and Goat Anti-Rabbit IgG (H&L) (#ab6721) obtained from Abcam (Cambridge, MA, USA), and were used at a 1/5000-1/10,000 dilutions. The antibodies information used in our current work was included in Supplementary Table 1.

### Human patients liver samples

Human donors liver specimens were harvested from adult donors with liver injury who underwent biopsy tissue samples or liver transplantation samples. The relevant control liver tissues were obtained from donors who were not eligible for liver transplantation for non-liver reasons. The hepatitis B virus (HBV) tissue, hepatitis C virus (HCV) tissue, steatohepatitis (NASH) phenotype samples and HCC liver samples were obtained and included in this study. Of note, liver samples from patients with any of the following conditions were excluded from the study: excessive drinking (alcohol > 70 g for female or alcohol > 140 g for male, per week), viral infection or drug abuse (including hepatitis B & C virus infection). Also, prior to this study, the samples of NASH in this cohort were collected from patients without taking statins or insulin. The NASH phenotype liver samples in this cohort were from patients who had taken pioglitazone (15–30 mg/day) for no more than 24 months. Liver sample donors and their families agree & sign written informed consent. Also, Human NASH-associated HCC tumor tissues and adjacent normal tissues were obtained from patients with biopsy-proven NASH-HCC. All the protocols involving human donors in this work were grounded on *the Ethical Principles for Medical Research Involving Human Subjects*,* Declaration of Helsink (64th WMA general assembly)*, and totally approved by the *Academic Committee of Experimental Animal Ethics*,* Use & Care Union* in Shandong First Medical University and other participating units.

### Animals and administration

The methodologies and protocols employed in this animal study were conducted in compliance with the Regulations of the People’s Republic of China on the Administration of Experimental Animals (Revised & Exposure Draft), as stipulated by the Ministry of Science and Technology of the People’s Republic of China (http://www.most.gov.cn). All mice were housed under standard laboratory conditions, with a light cycle of 12-h light/12-h dark (lights on at 7:00 a.m.) and maintained at a temperature of 22–25 °C in the specific-pathogen-free (SPF) environment. They had *ad libitum* access to water and were provided with either standard laboratory normal chow diet (NCD, #D09100304, BioPike, Beijing, China). Water bottles and cages underwent autoclaving prior to use. Cages containing standard corncob bedding were changed three times weekly. For all in vivo experiments, littermate control mice were utilized. The mouse genotype did not result in any observable differences in initial weight, health status, or immune function. Different groups were assigned in a randomized manner, and the investigators remained blinded to the allocation of these groups during both surgical procedures and outcome assessments.

### #1 Hepatocyte-specific *Notch1* deletion mouse (NOTCH^CKO^) and hepatocyte-specific *Keap1* deficiency mouse (KEAP1^CKO^) strains construction

Mice with a hepatocyte-specific deletion of Notch1 (NOTCH1^CKO^) were generated by using Alb-Cre recombinase, which expresses CRE recombinase exclusively in hepatocytes. Alb-Cre mice, (B6.FVB(129)-Tg(Alb1-cre)1Dlr/J) (RRID: IMSR_JAX:016832) and 129 S.Cg-Speer6-ps1Tg(Alb-cre)21Mgn/MdfJ (RRID: IMSR_JAX:035593) were obtained from the Jackson Laboratory (Bar Harbor, ME, USA). NOTCH1^*flox/flox*^ (NOTCH1^f/f^) mice with a C57BL/6 background were generated through CRISPR/Cas9-mediated genome editing, with two *loxP* sites inserted around exon 3 and 4 of the NOTCH1 gene. Homozygous floxed NOTCH1 mice (NOTCH1^f/f^) were crossed with Alb-Cre mice to generate double heterozygous floxed NOTCH1 mice (Alb-Cre; NOTCH1^*flox/+*^). Alb-Cre; NOTCH1^*flox/+*^ mice were then backcrossed to generate hepatocyte-specific *Notch1* knockout mice (NOTCH1^CKO^). Mice with wide type alleles and Cre expression served as controls (NOTCH1^f/f^).

Then, the Alb-Cre recombinase, which expresses CRE recombinase exclusively in hepatocytes, was used to create KEAP1^*flox/flox*^ (KEAP1^f/f^) mice with a C57BL/6 background. Two loxP sites were inserted around exon 2 of the Keap1 gene in KEAP1^*flox/flox*^ (KEAP1^f/f^) mice, which were then backcrossed with Alb-Cre mice to create hepatocyte-specific *Keap1* knockout mice (KEAP1^CKO^). For generation of hepatocyte-specific dual-deletion of Notch1-Keap1 (*NOTCH1-KEAP1*^DKO^), the *NOTCH1-KEAP1*^DKO^ mice were produced by mating NOTCH1^CKO^ with KEAP1^f/f^ mice. The obtained offspring without NOTCH1 and KEAP1 protein expression were identified and selected by western blotting assay and used for further indicated experiments.

### #2 Transgenic Rosa^*NOTCH1*^ mice construction and additional AAV-TBG-based transgenic mice models establishment

To construct mice with conditional overexpression of NOTCH1 alone, the Rosa^*NOTCH1*^ mice ground on C57BL/6 strain were constructed by NOTCH1 conditional knockin at the mouse locus of *Gt(ROSA)26Sor* by CRISPR-Cas9 genetic engineering editing system. The designed cassette of *Rosa26*-(pCAG)-*loxp*-STOP-*loxp*-m*Notch1*-pA section was inserted into 1st intron of *Rosa26*. Then, the targeting vector, guide RNA, and Cas9 were co-injected into eggs for Rosa^*NOTCH1*^ mouse establishment. In the indicated experiments, the conditional overexpression of *Notch1* in hepatocytes were triggered by injection of AAV-TBG-Cre vectors via intravenous injection and then determined by immunoblotting assay (HTG). Rosa^*NOTCH1*^ littermates with AAV-blank injection were used as controls. To restore the gene expression of *Notch1*, *Keap1* or *Notch1-Keap1*, the *NOTCH1-KEAP1*^DKO^ mice were injected with adenovirus (Ad)-loading Notch1 (Ad*NOTCH1*), Ad*KEAP1*, Ad*KEAP1 +* Ad*NOTCH1* to express NOTCH1, KEAP1 or both of NOTCH1 & KEAP1 protein, respectively. The *NOTCH1-KEAP1*^DKO^ mice injected with AdshRNA were treated with controls.

### Animal model construction

For the corresponding experiments in the current investigation, three distinct rodent liver injury models were built. The mice were given a week to become used to their new surroundings before any studies were conducted properly. The mice were housed in sterile conditions with a conventional 12-hour light/12-hour dark cycle, adequate water and food, and a constant temperature and humidity (managed by a Haier central air conditioner, #RFC140MXSAVB, China) of 25 °C ± 5 °C, 50–60%.

To establish the chronic liver inflammation model, mice were given intraperitoneal injections of carbon tetrachloride (CCl_4_) (#HY-Y0298, MedChemExpress, Shanghai, China) diluted 1:3 in corn oil (#T5137, TargetMol, Shanghai, China) or control (corn oil) at a dose of 0.5 µL/g of body weight twice a week for a total of 12 injections over 6 weeks in order to create the chronic liver inflammation model. After 48 h following the final treatment, the mice were sacrificed for next experimental procedure. On the other hand, the chronic liver inflammation model was created by injecting mice twice a week for a total of 12 injections with dimethylnitrosamine (DMN) (#HY-78177, MedChemExpress, Shanghai, China) dissolved in olive oil at a dose of 0.5 mg/kg body weight. The mice received olive oil alone were regarded as controls. Next, to determine the functional effect of NOTCH1-KEAP1 on occurrence of liver inflammation-related HCC, the NOTCH1^f/f^, NOTCH1^CKO^, Rosa^*NOTCH1*^ mice (with indicated AAV-TBG-Cre or AAV-TBG-Blank injection), *NOTCH1-KEAP1*^DKO^ mice (with indicated AdshRNA, Ad*KEAP1*, Ad*NOTCH1* or Ad*NOTCH1* + Ad*KEAP1* injection) were preconditioned with 25 mg/kg diethylnitrosamine (DEN) (#N109571, ALADDIN, Shanghai, China) via intraperitoneal injection, followed by CCL_4_ (0.5 mL/kg) treatment every two weeks, and euthanized at 16 weeks, respectively.

### Cell culture and administration

Human THLE2 cell line (#CRL-2706), human HCC-related cell lines MHCC97H, SNU-182, SNU-398 and Hep3B were purchased from the American Type Culture Collection (ATCC). All cell lines associating with corresponding in vitro or in vivo experiments were compulsorily examined for *mycoplasma* interference via polymerase chain reaction detection, and then cultured in indicated corresponding medium. The cell lines were respectively cultured in Dulbecco’s Modified Eagle Medium (Cat: BC-M-005, Bio-Channel Biotechnology Co., Ltd., China) or Roswell Park Memorial Institute (RPMI) 1640 medium (Cat: G4534, Servicebio^®^, Wuhan, China) containing 1% penicillin + streptomycin (Cat: BL505A; Biosharp Life Sciences), 10% premium quality FBS (Cat: 085–150, WISENT), and were maintained in a 5% CO_2_, 37℃ directly-heated type cell incubator (SANYO).

Isolation and protocols for the primary hepatocytes culture were performed according to previous reports [7]. Mouse primary cultured hepatocytes with indicated genes deletion used in this experiment were isolated and concentrated from indicated experiments’ animals by liver perfusion. In brief, after anesthesia with sodium pentobarbital, mice abdominal cavity was opened. Then, the liver samples were tardily perfused with 1×liver perfusion working solution (Cat: 17701038, Gibco™) and 1×liver digest working solution (Cat: 17703034, Gibco™) via the portal vein. Then, the digested liver tissue was filtered using a 100 μm steel mesh. The primary isolated hepatocytes were produced by harvesting the filter solution after 800 rpm centrifugalization (4℃, 5–10 min), and next purified using percoll-solution (Cat: 40501ES60, YEASEN, Shanghai, China). The isolated hepatocytes were maintained in corresponding DMEM medium (10% FBS & 1% penicillin-streptomycin) and then cultured in a 37℃, 5% CO_2_ condition. To mimic hepatic inflammation of in vivo experiments, the THLE2 cells, and mouse primary hepatocytes were administrated with the indicated dose of CCl_4_ or DMN to investigate the indicated signaling events.

### Construction of the knockout cell and knockdown lines

The establishment methods and procedures of targeted gene-deletion cell lines participated in this study were constructed as described previously [6, 7]. Briefly, THLE2 cell lines with *KEAP1* knockout (*KEAP1* KO), *NOTCH1* knockout (*NOTCH1* KO), *KEAP1-NOTCH1* double knockout (*KEAP1-NOTCH1* DKO) were established by CRISPR-Cas9 system, respectively. On the other hand, SNU-398, Hep3B, SNU-182 and MHCC97H cell with *KEAP1* knockout, *NOTCH1* knockout or *KEAP1-NOTCH1* double knockout were accordingly constructed, respectively. The designed small guide RNA (sgRNA) for these indicated genes were obtained to create the Cas9/sgRNA-loaded lentivirus. Ready-made small guide RNA (sgRNA) for establishment of knockout cells were purchased from Santa Cruz Biotechnology, Inc. The cells with indicated single gene knockout or double genes knockout were subjected to repeated screening according to CRISPR/Cas9 KO Plasmid product instruction. The obtained cell clones-containing gene deficiency were distinguished by western blotting. For establishment of adenovirus-mediated targeted genes knockdown in HCC cell lines or THLE2 cells, the short hairpin RNA (shRNA) for knockdown expression were obtained from Santa Cruz Biotechnology, Inc., were packed into adenovirus (Addgene, Watertown, MA, USA), respectively.

### Ferroptosis-related indexes determination

GSH and GSSG was quantified utilizing assay kit (#S0053) from Beyotime Biotechnology (Shanghai, China). MDA (#A003-1-2) levels were calculated using commercially available kit from Nanjing Jiancheng BioEngineering Institute (Jiangsu, China) following the manufacturer’s protocols. The Iron Assay Kit (#ab83366, Abcam) was utilized to assess Fe^2 +^ and/or Fe^3+^ concentrations according to the manufacturer’s instructions. Briefly, cells (1 × 10^6^) were resuspended in 100 µl of iron assay buffer and lysed via sonication. Following centrifugation, supernatants were incubated with the iron probe for a duration of thirty minutes at 37 °C. The absorbance values for each sample were subsequently measured at a wavelength of 593 nm. ELISA kit (#G01670, Westang, China) was used for the measurement of ferric ion under the instructions of manufacturer. The ATP levels in cells was examined using an Enhanced ATP Assay Kit (#S0027, Beyotime Biotechnology) following the manufacturer’s protocols by preparing an ATP standard curve and assessing the concentration of the supernatant accordingly. Meanwhile, the protein concentrations of the cellular supernatants were measured with a BCA protein assay kit according to the protocols provided by the manufacturer, and the ATP contents were finally calculated per one µg of protein. Tissues or cells were homogenized and centrifuged based on the manufacturers’ instructions.

### ROS production measurements

To examine cellular ROS production, the cells after treatments were washed in PBS and incubated with 10 µM of dichloro-dihydro-fluorescein diacetate (DCFH-DA, #S0034M, Beyotime Biotechnology) or dihydroethidium (DHE) (#HY-D0079, MCE) at 37 °C for 30 min according to the manufacturer’s procedures, respectively. After incubation, the cells were washed with PBS to remove unincorporated DCFH-DA or DHE. The fluorescence intensity of the cells was finally and immediately examined under a fluorescence microscope (Leica, Germany). Mitochondrial ROS production in cells were also examined using a Mito-SOX reagent (#HY-D1055, MCE) staining following the manufacturer’s protocols. Cells were seeded on 6-well plates. After adherence and treatments, the culture medium was removed and washed once with PBS. Then, 1 ml of MitoSOX Red staining working solution was added to each well and incubated at 37 °C for 30 min, followed by washing with PBS. Nuclei were stained using Hoechst 33,342 (#P0133, Beyotime Biotechnology) for another 20 min. After washing with PBS, the cells were observed under a fluorescence microscope (Leica, Germany). The fluorescence intensity was analyzed to reflect the production of cellular and mitochondrial ROS.

### BODIPY 581/591-C11 and MitoTracker assay

BODIPY 581/591-C11 detection was used to examine lipid peroxidation in the treated cells. In brief, cells after treatments were incubated with 2 µM of BODIPY 581/591-C11 (#D3861, Thermo Fisher Scientific) for 30 min. Next, the cells were washed with PBS twice. Images were captured using a fluorescence microscope (Leica, Germany). The excitation and emission wavelengths for the oxidized form were within the ranges of 460–495 nm and 510–550 nm, respectively. In contrast, the excitation and emission wavelengths for the reduced form were within the ranges of 565–581 nm and 585–591 nm, respectively. We utilized the ratio of oxidized to reduced forms as an indicator of lipid peroxidation. As for MitoTracker assay, a Mito-Tracker Red CMXRos kit (#C1035, Beyotime Biotechnology) was used to examine the mitochondrial dysfunction. After each treatment, cells were incubated with 200 nM of Mito-Tracker Red CMXRos working solution for 30 min at 37 °C, followed by nuclei staining using Hoechst 33,342 (#P0133, Beyotime Biotechnology) for another 20 min. After washing with PBS, the cells were observed under a confocal microscope (FV3000, Olympus, Japan). For image quantification, analysis was performed using Image-J software (Version 1.8.0, NIH, USA), ensuring that all evaluations were performed blindly.

### Plasmids preparation and transfection

With the objective of enhancing NOTCH1 and KEAP1 expression, full-length cDNA expression vectors for Homo sapiens were generated through PCR-based amplification. These vectors were subsequently cloned into pcDNA3.1 plasmids, which were tagged with either 3×HA or 3×Flag (Addgene, Watertown, MA, USA). A collection of truncated NOTCH1 (Flag-△PEST, Flag-△TAD, Flag-△ANK, Flag-△RAM) and KEAP1 (HA-△NTR, HA-△BTB, HA-△IVR, HA-△KELCH/CTR) expression plasmids were accordingly prepared by PCR and cloned into the pcDNA3.1 plasmids tagged with Flag and HA expression vectors, respectively. The transfection of vectors into cells was performed using ViaFect™ Transfection Reagent (#E4982, Promega, Beijing, China) according to the manufacturer’s instructions. *Homo sapiens* full-length NOTCH1 sequences and designed short hairpin RNA (shRNA) targeting human NOTCH1 (sh*NOTCH1*) (sequences RNA: CCGGGACATCACGGATCATAT; Sigma-Aldrich, USA) and human wild type NOTCH1 sequences with ANK domain mutation were respectively packaged into adenovirus by Adeno-X™ Adenoviral System 3 Kit (#632269, Takara Bio Inc.). The control groups for expression suppression (knockdown) or overexpression were served by the empty adenovirus constructs, defined as Ad-shRNA and Ad-GFP, respectively. The adenovirus (Ad) vectors that were produced were purified and quantified to a tire of 5 × 10^10^ plaque forming units (PFU) with the Vivapure AdenoPACK purification kit (#VS-AVPQ022, Sartorius, Shanghai, China) standardly following the manufacturer’s protocols. All plasmids underwent verification through sequencing post-construction. KEAP1 gene expression was silenced by the use of siRNA purchased from Santa Cruz Biotechnology Inc (#sc-43878, CA, USA), and the control siRNA (#sc-37007, Santa Cruz Biotechnology Inc) was included. Cells were transfected with siRNAs using Lipofectamine RNAi Max (#13778075, Invitrogen™, Thermo Fisher Scientific, Shanghai, China) as per manufacturer’s instructions.

### Histopathological analysis

To perform histopathologic and immunohistochemical assay, the tissue was consequently fixed with 4% formaldehyde-tissue fixative solution (Cat: 80096618, Sinopharm Chemical Reagent Co., Ltd., China), embedded in paraffin wax (Cat: 69018961, Sinopharm Chemical Reagent Co., Ltd., China), and then sectioned transversely. The tissue slices were subjected to H&E staining (Cat: G1120, Hematoxylin-Eosin/HE Staining Kit, Solarbio Life Sciences, Beijing, China) to visualize the degree of hepatic inflammation. Moreover, to visualize collagen contents in liver tissue, slices were subjected to Masson staining (Cat: abs9348, Masson’s Trichrome Stain Kit, Absin, Shanghai, China) and sirius red staining (Cat: PH1099, Enhanced Sirius Red Staining Kit, Scientific Phygene^®^, China). To perform immunohistochemical analysis, parrffin-embedded slices were dewaxed prior to incubation with indicated primary antibodies at 4 °C refrigeration overnight. The corresponding anti-rabbit IgG, anti-mouse IgG or anti-goat IgG antibodies were used as the secondary antibody (Thermo Fisher Scientific). All histological experiments were performed according to standard protocols presented in the reagent instructions and operation manual, and were performed by three histologists blinded to treatment procedures. Pictures were displayed using a light microscope (Leica, Germany) for samples section detection and a confocal laser microscopy system (FV3000, Olympus, Japan) for immunofluorescence section detection.

### Co-immunoprecipitation (Co-IP)

Cells or liver tissues were disintegrated in ice-chilled IP buffer (20 mM Tris-HCl pH 7.4, 150 mM NaCl, 1 mM EDTA and 1% Triton X-100) encompassing the complete Protease Inhibitor (#04693132001, Merck Roche, Germany) and PhosStop phosphatase inhibitor (#4906837001, Merck Roche, Germany). The disintegrated mixtures were centrifuged at 12,000 g for 15 min and subsequently gathered and incubated with the designated antibodies and Protein G Agarose beads (#11719416001, Merck Roche, Germany) throughout the night at 4 °C. After being washed three times in frigid immunoprecipitation buffer, the immunocomplexes were amassed and exposed to immunoblotting employing the indicated primary antibodies and corresponding secondary antibodies.

### Glutathione S-transferase (GST) Pull-down assay

The approaches of Glutathione S-transferase (GST) Pull-down carried out in accordance with our previous reports [7, 8]. Direct protein binding between indicated proteins was performed using the GST pull-down analyses. The MagneGST™ Pull-Down System Kit (#V8870, Promega, Beijing, China) was used to detect the protein binding in this regard of the experiment. Briefly, the *Rosetta*-DE3 competent cells were transformed with the corresponding plasmid based on pGEx-4T-1/GST and then induced vectors expression by treating with 0.5 m Misopropyl β-D-thiogalactoside (IPTG) (Cat: HY-15921, MedChemExpress, Shanghai, China). Then, the extractions were co-treated with corresponding GST particles for 1 h, 4℃. Interacting proteins were eluted in elution buffer followed by western blotting analysis using the indicated antibody. The *Rosetta*-DE3 competent cells expressing only GST-tag were regarded as the control.

### RNA extraction, quality control and quantitative PCR (qPCR) assay

TRNzol Universal Reagent (Cat: DP424, TIANGEN^®^, Beijing, China) was used to separate the total RNA from the required liver tissue or cells in accordance with the procedures suggested by the product specification. The extracted RNA was stored for a maximum of 14 days at -80 °C. A Nanodrop photometer (Tecan) analysis was used to establish the absorption of RNA contents at 260 nm. Verification of the 260 nm/280 nm adsorption ratio (values > 2.00) ensured the highest RNA purity. The universal RT-PCR Kit (M-MLV) (#RP1100, Solarbio Life Sciences, Beijing, China) and TaqMan^®^ Universal PCR Master Mix (#P/N 4304437, Applied Biosystems™) were then used to inversely transcribe 1 µg of isolated RNA. After one hour of inverse transcription at 42 °C, the enzymes were inactivated for ten minutes at 70 °C. SensiMix SYBR Master Mix Kits (#QP100001, OriGene Technologies) and PowerUpTM SYBR™ Green (#A25742, Thermo Fisher Scientific) were used in the PCR process in ABI PRISM 7900HT equipment (Applied Biosystems). Sangon Biotech (Shanghai, China) created the specific primer sequences for the important genes linked to lipid metabolism, inflammation, and fibrosis, or OriGene Technologies, Inc. provided pre-made primers. Fold difference data were calculated using the 2(-ΔΔCt) expression, where ΔCt is the cycle threshold difference between GAPDH and the target gene and ΔΔCt is the relative change in the differences between the control and indicated experimental groups. The sequences of primer were included in Supplementary Table 2.

### Immunoblotting detection

To perform immunoblotting analyses, liver tissue or cells were subjected to RIPA (radio immunoprecipitation assay) lysis buffer (Cat: PH0317, Scientific Phygene^®^) to yield lysates. Then, the final supernatant was compressed by centrifugation at 4℃, 13,000 rpm for 30 min. Protein concentration of obtained supernatant was confirmed by BCA Protein Quantification Kit with fat-free BSA as a control. The total extracted protein samples were then processed to immunoblotting assay. The same amounts of total protein isolated from the indicated cells or liver samples were processed to 10% or 12% SDS/PAGE gel (Cat: 20328ES50, SDS/PAGE Gel Preparation Kit, YEASEN, Shanghai, China) and then subjected to a 0.45 µM Immun-Blot polyvinylidene fluoride (PVDF) membrane (Cat: 10600023, GE Healthcare Life Science, Germany) via wetting transfer method, followed by western blotting using the assigned primary antibodies. Subsequently, the immunoblotting membranes were incubated with blocking buffer (5% nonfat-dried milk) (Cat: LP0033B, Biosharp^®^ Life Science, Beijing, China) in 1×TBS working buffer solution (Cat: PH1402, Scientific Phygene^®^) containing 0.1% Tween-20 (Cat: 9005-64-5, Sinopharm Chemical Reagent Co., Ltd., China) (1×TBST working buffer solution) for 1 h, and mingled with the assigned primary antibodies at 4 °C refrigeration overnight. Then, the PVDF membranes were washed in 1×TBST working buffer solution for 3 times, followed by co-treated with horseradish peroxidase-tagged goat anti-rabbit IgG (H + L) or anti-mouse IgG (H + L) (Cat: 33201ES60; Cat: 33101ES60, YEASEN, Shanghai, China) for 1–2 h at 25–30 °C. Immunoblotting membranes were visualized by New-SUPER (Hypersensitivity Type) ECL Kit (Cat: KGP1128, KeyGen BioTECH, China) and exposed to FUJI Medical X-ray film (Cat: 4741023952, FUJIFILM, China). Corresponding protein levels were then calculated as gray-scale score(Version 1.8.0, Microsoft Windows, Image J, NIH, USA) and normalized to GAPDH and standardized as a fold change of controls.

### Cell viability assay and cell penetration detection

Cells were seeded into 96-well plates and cultured for the indicated times. Cell viability was evaluated using a CyQUANT™ kit (Cat#: C7026, Thermo Fisher Scientific). The value of OD450 was calculated by micro-plate reader (Varioskan™ LUX, Thermo Fisher Scientific) and presented as the mean ± SEM from three individual experiments. With the analysis of cell penetration, the cultured cells were incubated with different FITC-marked tested chimeras for indicated times. The cells with tested chimeras penetration were determined and calculated using micro-plate reader and presented as the mean ± SEM from three individual experiments.

### Immunofluorescence (IF) staining assay

Immunofluorescence staining was carried out on the cells and paraffin-embedded liver sections, adhering to the identical deparaffinization, antigen retrieval, and rehydration procedures as the IHC staining process. Cells post treatments were washed with PBS twice. Then, liver sections and the cells were fixed with 4% paraformaldehyde for 20 min and washed with PBS twice, followed by 1 h of blockage in 10% donkey serum containing 0.2% Triton X-100 (#P0096, Beyotime, Shanghai, China) at 37 °C. Tissue sections and cell samples were subsequently incubated overnight at 4 °C with the following primary antibodies, including NOTCH1 (#MA5-11961 or #MA5-32080), COL1A1 (#GB11022), α-SMA (#14-9760-82), GPX4 (#GB124327), KEAP1 (#PA5-99434), HES1 (#sc-166410), and NRF2 (#PA5-27882). On the next day, secondary antibodies including Goat Anti-Mouse IgG H&L (Alexa Fluor^®^ 647) (#ab150115; 1/300 dilutions), Donkey Anti-Rabbit IgG H&L (Alexa Fluor^®^ 647) (#ab150075; 1/300 dilutions), Goat Anti-Rabbit IgG H&L (Alexa Fluor^®^ 488) (#ab150077; 1/400 dilutions), and Goat Anti-Mouse IgG H&L (Alexa Fluor^®^ 488) (#ab150113; 1/400 dilutions) were employed for 1 h of incubation at 37 °C. Nuclei were stained with DAPI (#P0131, Beyotime Biotechnology) for final 5 min. Images were obtained using an Olympus FV3000 confocal microscope (Olympus, Japan). Image-J software (Version 1.8.0, NIH, USA) was used for fluorescent intensity evaluation by a blinded fashion.

### Detection of hepatic ROS contents

The liver tissues for ROS production were evaluated by DHE (MCE). Briefly, cryosections (10 μm thick) were incubated with 10 µM DHE at 37 °C for 30 min. Fluorescence was detected by a confocal microscope system (FV3000, Olympus, Japan). Then, 10 randomly selected nonoverlapping fields from each sample were analyzed by Image-J (Version 1.8.0, NIH, USA) in a blinded manner. The fluorescence intensity was analyzed to reflect the production of cellular and mitochondrial ROS.

### RNA-sequencing (RNA-Seq) analysis

The technology of transcriptome sequencing analysis was underpinned by (Servicebio, Wuhan, Hubei, China). The principal experimental procedures encompassed total RNA extraction, assessment, quantification, library establishment, clustering and sequencing, and ultimate data analysis. The library quality was evaluated on the Agilent Bioanalyzer 2100 system. Differential expression analysis was executed using the DESeq2 R package. Genes with an adjusted *p* values less 0.05 and absolute fold change ≥ 1.5 were regarded as significantly differentially expressed. GO enrichment analysis of differentially expressed genes (DEGs) was conducted using the cluster Profiler R package. GSEA analysis of diverse signaling pathways was carried out based on the Molecular Signatures Database of the GSEA web interface.

### Fractionation assay

Fractionation of cell proteins was performed using the Cytosolic and Nuclear Extraction Kit (Pierce) Subcellular Protein Fractionation Kit (Novus Biologicals). The nucleus was lysed with lysis buffer (50 mM TEA, pH 7.3, 150 mM NaCl, 4% SDS, 1×protease inhibitor cocktail (Roche, EDTA free), 1500 units/ml benzonase nuclease and 2 mM PMSF). EDTA was added to a final concentration of 5 mM after solution removal. The cytoplasmic proteins were diluted with the lysis buffer containing 5 mM EDTA before APE assay.

### Prediction of protein–protein interactions

The protein structure of NOTCH1 and KEAP1 was downloaded from Uniprot database (https://www.uniprot.org/id-mapping/), and the interaction mode of NOTCH1 and KEAP1 was studied by Hdock (http://hdock.phys.hust.edu.cn/). Pymol 2.3.0 software (The PyMOL Molecular Graphics System, 2023) was used to analyze the interaction mode of the docking result.

### Stable isotope-labeling by amino acids in cell culture (SILAC)

The transfected human THLE2 cells underwent stable isotope labeling through cultivation by seven doubling times in ^13^C_6_-lysine/^13^C_6_-arginine-containing RPMI 1640 media (Merck), which served as a heavy isotope-labeled group because it is challenging to label cells in living organisms by isotopes. The SILAC protocol used in our current work was performed in accordance with previous reports with certain modifications [54, 55]. To measure the level of stable isotope incorporation after seven doubling times, a mass spectrometer was applied to verify the level of heavy isotope incorporation. The results showed that the level of heavy isotope incorporation in the cells was 97–98%. The non-transfected THLE2 cells (control) were classified as light isotope-labeled groups. Stable heavy isotope-labeled THLE2 cells were individually lysed in RIPA buffer (0.5% Triton X-100, 0.5% NP-40, and 0.1% SDS in 50 mM Tris buffer, pH 7.8) and centrifuged at 17,500 × g at 4 °C for 15 min. The protein concentrations of each sample were measured using Bradford assay (Bio-Rad). The control group cell lysates were gently mixed with heavy isotope-labeled THLE2 cell lysates at a protein ratio of 1:1. The lysate mixture was subjected to SDS-PAGE and the running gel was then divided into 15 fractions. The proteins in each gel fraction were incubated in 5 mM of dithiothreitol at 60 °C for 20 min and 20 mM of iodoacetamide at room temperature for 10 min. For protein in-gel trypsinization, the proteins in each gel fraction were incubated in Sequencing Grade Modified Trypsin (10 µg/ml trypsin, Promega) at 37 °C overnight. The samples were acidified using 1% trifluoroacetic acid (Merck), and the peptides were extracted using 3, 50, and 100% acetonitrile. The extracts were dried by a SpeedVac vacuum (Thermo Fisher). The peptides were resuspended in 0.1% trifluoroacetic acid and purified using the ZipTip C18 column (Merck). The peptides in the column were washed three times with 10 µl of 0.1% trifluoroacetic acid and then eluted using 30 µl of 50% acetonitrile with 0.1% trifluoroacetic acid. One third of the collected sample was used for LC-MS/MS analysis. Subsequently, the peptides were separated on a 40 cm, 75 μm internal diameter packed emitter column (Coann emitter from MS Wil, Poroshell EC C18 2.7 micron medium from Agilent) using an EASY-nLC 1200 (Thermo Fisher Scientific). The column was maintained at 50 °C. Buffer A and B were 0.1% formic acid in water and 0.1% formic acid in 80% acetonitrile, respectively. Peptides were separated at a flow rate of 300 nl / min, on a gradient from 6 to 31% buffer B for 57 min, from 31 to 44% buffer B for 5 min, followed by a higher organic wash. Eluting peptides were analysed on a Orbitrap Fusion Tribrid mass spectrometer (Thermo Fisher Scientific). Peptide precursor m/z measurements were carried out at 60,000 resolution in the 350 to 1500 m/z range. The most intense precursors with charge state from 2 to 7 only were selected for higher-energy C-trap dissociation fragmentation using an isolation window of 1.6 and 27% normalised collision energy. The cycle time was set to 1 s. The m/z values of the peptide fragments were measured at a resolution of 30,000 using an AGC target of 2e5 and 54 ms maximum injection time. Upon fragmentation, precursors were put on a dynamic exclusion list for 45 s. Next, raw data were analysed with MaxQuant version 1.6.1.0. Peptide fragmentation spectra were searched against the canonical and sequences of the Mus musculus reference proteome (proteome ID UP000000589, downloaded December 2018 from UniProt). Methionine oxidation and protein N-terminal acetylation were set as variable modifications; cysteine carbamidomethylation was set as fixed modification. Multiplicity was set to two; Arg10 and Lys8 were set as heavy labels. The digestion parameters were set to ‘specific’ and ‘Trypsin/P’, The minimum number of peptides and razor peptides for protein identification was 1; the minimum number of unique peptides was 0. Protein identification was performed at a peptide spectrum match and protein false discovery rate of 0.01. The ‘second peptide’ option was on. Differential abundance analysis was performed using limma, version 3.34.9 in R, version 3.4.3. Protein groups annotated as Potential contaminant, Reverse, or Only identified by site were removed prior to the statistical analysis. The mass spectrometry proteomics data have been deposited to the ProteomeXchange Consortium via the PRIDE partner repository with the dataset identifier PXD055907.

### Statistical analysis

The associated results showed in this work were processed independently at least 3 times. All raw data involving this work were independently analyzed by suitable statistic protocols, as assigned in the figures legends. Unless otherwise stated, quantitative scores of data are presented as mean ± SEM. ANOVA was used for comparison approach, multiple groups were performed using *Dunnett’ s* multiple comparison test, and two groups comparison was performed using 2 tailed Student’ s *t* test. GraphPad Prism Software (Version 9.4.1.681 for Microsoft Windows; GraphPad Software, San Diego, USA), the R packages and IBM SPSS Statistics (Version 25.0, Microsoft Windows, IBM, USA) were performed for the final data presentation. *P* < 0.05 means statistically significant difference.

## Results

### Hepatocyte NOTCH1 expression is positively correlated with liver injury severity but negatively correlated with NRF2 levels

**Fig. 1:**
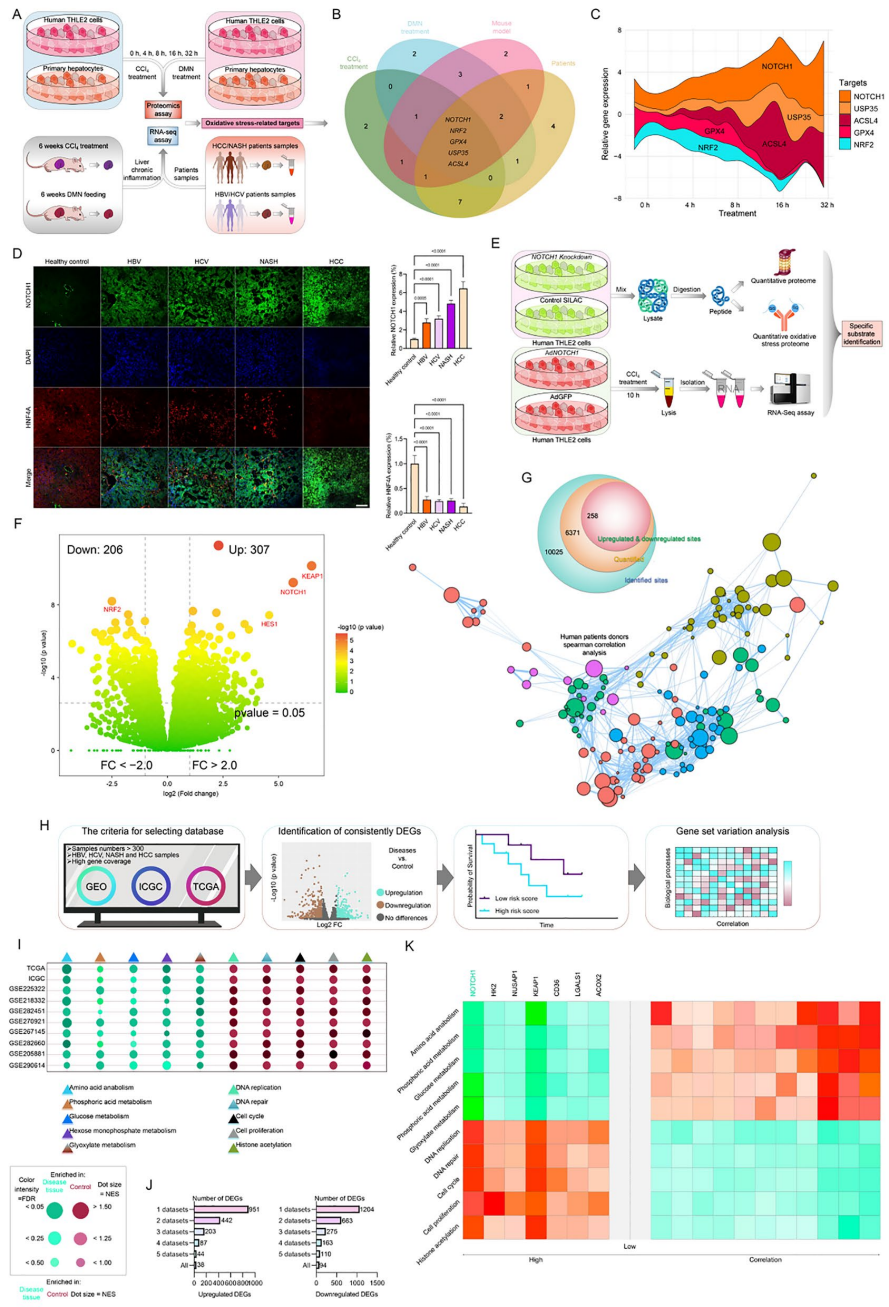
Identification of potential indicators involved in chronic liver inflammation and hepatocellular carcinoma (HCC)-related biological processes. (**A**) Experimental design showing the protocol of identifying oxidative stress-associated indicators in response to time-course of 1% CCl_4_-, 10 mM DMN-induced human THLE2 cells and mouse primary hepatocytes, or in 6-weeks of CCl_4_ (0.5 µL/g BW)- or DMN (0.5 mg/kg BW)-fed WT mice, or in liver samples from HCC, NASH, HBV, and HCV patients. (**B**) Venn diagram showing the Top 5 distinguishable expressed oxidative stress-associated candidates (i.e., NOTCH1, NRF2, GPX4, USP35 and ACSL4) in intersection of 4 separate samples data-set. (**C**) Waterfall streamgraph showing the relative mRNA expression profile of the 5 genes in the indicated groups. (**D**) Representative immunofluorescence images of NOTCH1 and HNF4A co-expression in lives of human samples with healthy control, HBV, HCV, NASH and HCC phenotype with fluorescence intensity measurement (magnification, 100×, *n* = 10 samples). (**E**) The flowchart showing the experimental procedure for the quantified proteome and protein interaction assay of NOTCH1. (**F**) Volcano plot indicating genes expression variation in human THLE2 cells after CCl_4_ treatment. (**G**) Number of identified oxidative stress-related upregulated & downregulated sites. (**H**) A screening protocol to highlight assumed gene candidates. (**I**) The physiopathology and biological processes associated with metabolism of HBV, HCV, NASH, and HCC samples were found to differ from those of controls in a number of databases, including TCGA, ICGC, and the NCBI Gene Expression Omnibus (GEO) datasets (GEO: GSE225322, GSE218332, GSE282451, GSE270921, GSE267145, GSE282660, GSE205881, and GSE290614). (**J**) The total number of differentially expressed genes that crossed over into different databases was tallied after gene differential expression analysis. (**K**) The molecular pathways influencing metabolism and the beginning of liver diseases are represented by seven upregulated DEGs. Data are presented as mean ± SEM. The associated experiments were performed independently at least three times. *P* < 0.05 indicates statistical significance

We examined the levels of candidates in vitro and in liver tissue taken from mice modelled by CCl_4_ or DMN, as well as human patients with HCC, NASH, HCV, and HBV pathological phenotypes, in order to examine their participation in the setting of liver injury. As shown in Figs. 1A and B, we discovered that the top 5 distinguishable expressed candidates, NOTC1, NRF2, GPX4, USP35, and ACSL4 were highlighted in the intersection of these experiment groups in response to the time-course of 1% CCl_4_-, 10 mM DMN-treated human THLE2 cells or primary hepatocytes, 6-weeks of CCl_4_ (0.5 µL/g BW)- or DMN (0.5 mg/kg BW)-fed WT mice, or in samples from HCC, NASH, HBV, and HCV patients. Next, the time-course analysis of the differential expression genes, or DEGs, in the two species of CCl_4_- and DMN-induced hepatocytes was conducted. Five indicators linked to oxidative stress, i.e., NOTCH1, NRF2, GPX4, USP35, and ACSL4 met the selection criteria for intersection screening, as anticipated. Of these, NOTCH1 showed a significant upregulation throughout the analysis, while NRF2 showed a marked downregulation in the dateset (Fig. 1C). NOTCH1 protein and related mRNA expression were found to be elevated in human liver samples based on expression patterns (Fig. 1D). Furthermore, we used the same liver tissue samples (patients and mouse) and mouse models. Hepatocytes and non-parenchymal liver cells, as well as the nuclear and cytoplasmic components of hepatocytes, were isolated respectively using Cytosolic and Nuclear Extraction Kit (Pierce) and Percoll (Sigma) (Supplementary Fig. 1A). As expected, we confirmed that the isolated hepatocytes by immunoblotting analysis showed that the expression abundance of NOTCH1 protein was significantly upregulated in liver samples of HBV, HCV, HCC, NAFLD and NASH compared with the control group. Further analysis of the cytoplasmic and nuclear separation components of the liver samples confirmed that the expression of NICD1 was mainly concentrated in the nucleus, and the expression of HNF4A was significantly inhibited in the nucleus (Supplementary Fig. 1B). This is consistent with our current research and previous reported studies. Also, we used the same experimental method to extract and isolate hepatocytes from liver samples of mouse models with liver injury induced by CCl_4_ and DMN. It was confirmed that under normal physiological conditions, the expression abundance of NOTCH1 in hepatocytes was very low, but when liver injury pathology occurred, its expression grade significantly increased (Supplementary Fig. 1C). To further determination, we extracted serum samples from patients with HCC, NASH, HCV and HBV, mixed them with fresh culture medium in a 1:1 ratio, and further cultured THLE2 cells for 10 h. The immunofluorescence detection results showed that compared with the control group (only cultured with medium), in the HCC, NASH, HCV and HBV groups, NICD1 was actively concentrated in the nucleus, while the expression of HNF4A in the nucleus was significantly reduced (Supplementary Fig. 1D). These results are consistent with the above western blotting results and our immunofluorescence results.

Given the strong association between NOTCH1 and the pathophysiology of liver injury as reported in earlier studies [14, 21, 22], we conducted a quantitative proteomic study of stable isotope-labeled amino acids in cell culture (SILAC) using mass spectrometry. Notably, we also demonstrated NOTCH1’s possible substrate and established that it was markedly elevated in vitro in response to the 10-hour CCl_4_ challenge (Fig. 1E-G). Then, by comparing the Ad*NOTCH1* and control groups in vitro, RNA-Seq analysis was carried out consistently to validate the role of NOTCH1 in the course of CCl_4_-induced hepatocyte damage. Among the identified differentially expressed genes (DEGs), which include *NOTCH1*, *HEY1*, and *HES1*, 62 genes may be linked to NOTCH1 in the mediation of fibrosis, oxidative stress, ferroptosis, liver injury complications, and the NRF2 pathway (Supplementary Fig. 1E-J). To further identify interesting genes that control the progression of liver diseases, however, we looked at the expression matrix of liver-associated diseases and nearby healthy tissues in public datasets that included high-coverage gene profiling data in the TCGA, ICGC, and NCBI Gene Expression Omnibus (GEO) databases (GEO: GSE225322, GSE218332, GSE282451, GSE270921, GSE267145, GSE282660, GSE205881, and GSE290614) (Fig. 1H). This was done in light of the changes of NOTCH1 expression in liver samples. The gathered samples showed a significant enrichment of tumor-related events and metabolic activities (Fig. 1I). We found 94 differentially expressed genes (DEGs) that were downregulated and 38 DEGs that were conservatively elevated throughout the ten datasets we chose (Fig. 1J). Notably, the top 7 elevated DEGs were found based on clinical data from the TCGA and ICGC databases because they had a strong relationship with survival rates (Fig. 1K).

**Fig. 2:**
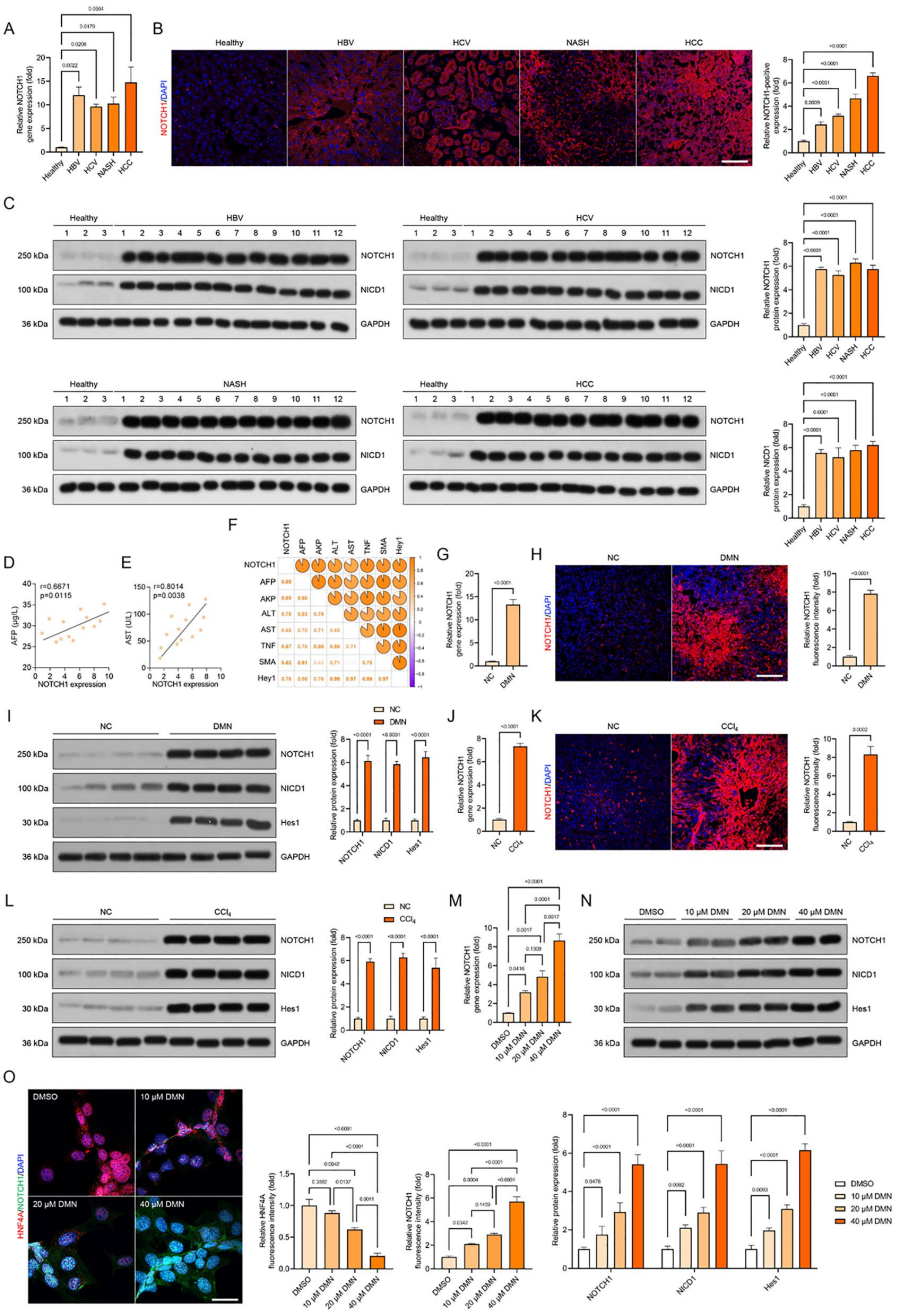
NOTCH1 (NICD1) signaling is enhanced in liver samples of chronic liver injury patients and mice. (**A**) Relative gene expression analysis of NOTCH1 in liver specimens from patients with HBV, HCV, NASH and HCC pathological phenotype (*n* = 12 samples). (**B**) Representative immunofluorescence images of NOTCH1 expression in liver samples of patients with HBV, HCV, NASH and HCC pathological phenotype (magnification, 100×, *n* = 10 samples). (**C**) Representative western blotting showing the NOTCH1 and NICD1 protein expression in liver samples isolated from patients with HBV, HCV, NASH and HCC pathological phenotype (*n* = 12 samples). (**D**,** E**) Pearson’s r correlation analysis of AFP levels and NOTCH1 levels, and AST contents and NOTCH1 levels in patients (*n* = 12 samples). (**F**) Multiple Pearson multiple correlation analysis for human subjects showing the comprehensive correlation between NOTCH1 protein expression levels and indicated parameter indexes (*n* = 12 indices each parameter). Utilizing DMN- and CCl_4_-induced mice, NOTCH1 expression alterations were investigated in mouse models with liver samples. (**G**) Relative gene expression assay of NOTCH1 in livers of control group (NC) and DMN-treated group (*n* = 10 samples). (**H**) Representative immunofluorescence images of NOTCH1 expression in liver samples of NC and DMN group (*n* = 10 samples). (**I**) Western blotting analysis showing the NOTCH1, NICD1 and Hes1 expression in liver tissue isolated from indicated groups (*n* = 4 samples). (**J**) Relative gene expression assay of NOTCH1 in livers of control group (NC) and CCl_4_-induced group (*n* = 10 samples). (**K**) Representative immunofluorescence images of NOTCH1 expression in liver samples of NC and CCl_4_ group (*n* = 10 samples). (**L**) Western blotting analysis showing the NOTCH1, NICD1 and Hes1 expression in liver tissue isolated from NC or CCl_4_ group (*n* = 4 samples). (**M**,** N**) A dose-dependent rise in NOTCH1 gene expression levels and protein expression was detected in human THLE2 cells following 10 h of DMN incubation (10 µM, 20 µM, and 40 µM) (*n* = 10 samples). (**O**) Representative immunofluorescence images of NOTCH1 and HNF4A co-expression in the indicated groups (magnification, 400×, *n* = 10 samples). Data are presented as mean ± SEM. The associated experiments were performed independently at least three times. *P* < 0.05 indicates statistical significance

Furthermore, to better understand how NOTCH1 signaling contributes to the development of liver injury, we first noticed that liver specimens from patients with HBV, HCV, NASH, and HCC had significantly higher NOTCH1 mRNA expression levels than those from healthy controls (Fig. 2A). The elevated NOTCH1 expression levels in these patients’ liver tissues, especially in the hepatocyte (Fig. 2B), were further revealed by immunofluorescence results and validated by western blotting analysis (Fig. 2C). These indicators of liver injury progression, including the cancer marker alpha-fetoprotein (AFP), the fibrosis marker alpha-smooth muscle actin (α-SMA), the liver injury markers alanine aminotransferase (ALT), alkaline phosphatase (AKP), and aspartate aminotransferase (AST), showed a positive correlation with changes in NOTCH1 protein expression, according to a Pearson analysis (Fig. 2D, E). Additionally, several Pearson results demonstrated that NOTCH1 expression levels were positively correlated with AFP, AKP, ALT, AST, α-SMA, Hey1, inflammatory marker tumor necrosis factor (TNF), and AKP (Fig. 2F). Then, utilizing DMN- and CCl_4_-induced mice, NOTCH1 expression alterations were investigated in mouse models with liver samples to pinpoint these findings. As anticipated, qPCR, immunofluorescence (IF), and western blot analysis revealed increased liver NOTCH1 mRNA and protein expression levels in the DMN- and CCl_4_-treated mice in comparison to the control (NC) group. Additionally, overexpression of NICD1 and Hes1 was seen (Fig. 2G-L). Meanwhile, we further verified that a dose-dependent rise in NOTCH1, NICD1, and/or Hes1 expression levels was seen in human THLE2 cells following 10 h of DMN incubation (10 µM, 20 µM, and 40 µM) (Fig. 2M-O). These findings suggest that the development of liver damage brought on by oxidative stress may be connected to the rise in hepatocyte NOTCH1.

### Hepatocyte-specific *NOTCH1* knockout ameliorates CCl_4_-mediated liver inflammation pathological phenotypes

**Fig. 3:**
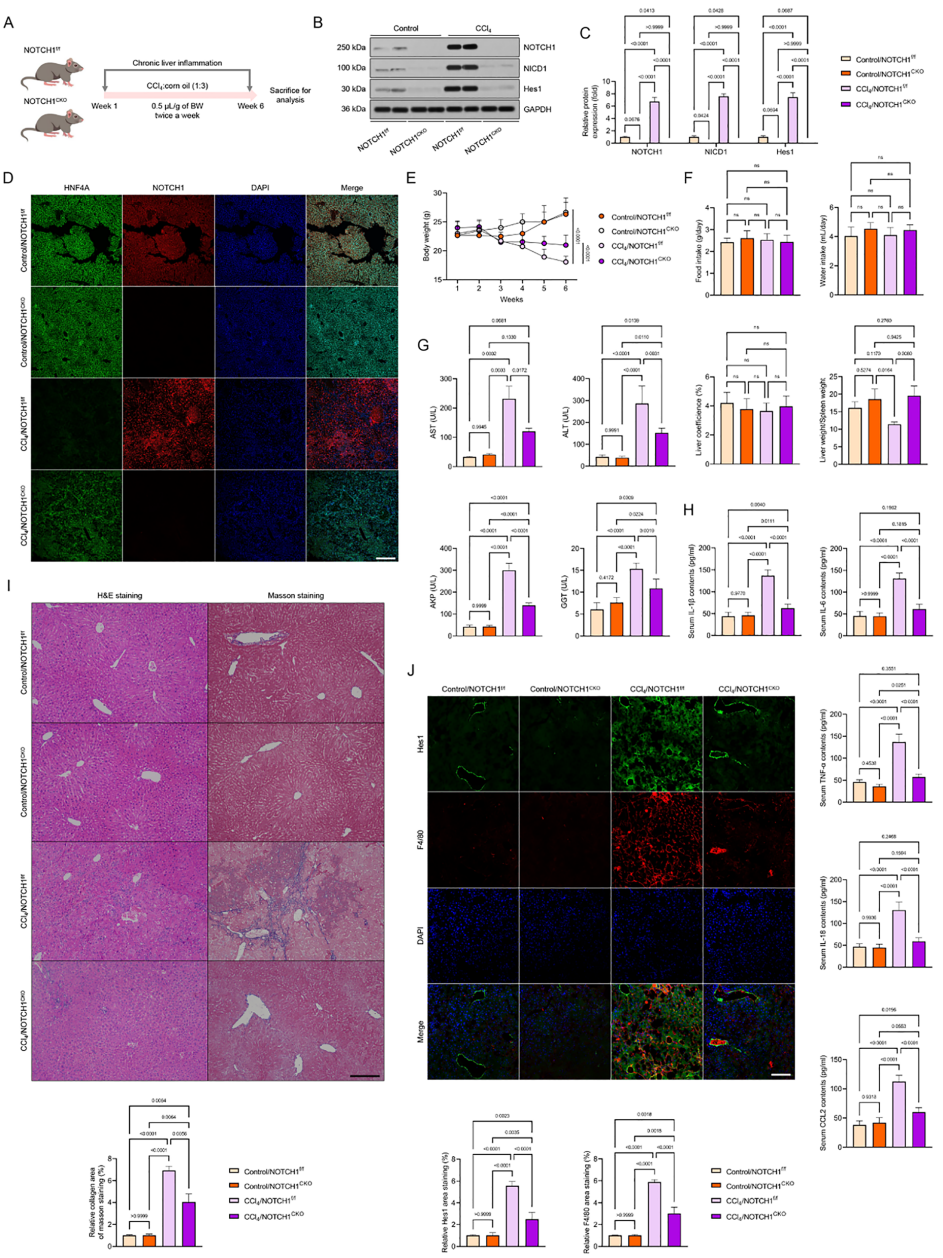
Hepatocyte-specific *NOTCH1* knockout ameliorates hepatocellular inflammation injury in CCl_4_-treated mice. (**A**) Schematic of experimental procedures examining the effects of hepatocyte-specific *NOTCH1* knockout (NOTCH1^CKO^) on CCl_4_-treated mice; NOTCH1^f/f^ group served as the control. (**B**,** C**) Western blot and quantification of NOTCH1, NICD1 and Hes1 expression in the isolated liver samples of control and CCl_4_-treated mice (*n* = 4 mice per group). (**D**) Representative immunofluorescence images of NOTCH1 and HNF4A expression in liver samples of indicated groups (magnification, 100×, *n* = 10 samples). Records of body weight (**E**), food intake, water intake, liver coefficience, liver-to-spleen ratio (**F**), serum AST, ALT, AKP & GGT contents (**G**), serum pro-inflammatory cytokines TNF-α, IL-6, IL-18, CCL2, and IL-1β levels (**H**) in Control/NOTCH1^f/f^, Control/NOTCH1^CKO^, CCl_4_/NOTCH1^f/f^, and CCl_4_/NOTCH1^CKO^ group (*n* = 10 mice per group). (**I**) Representative liver histological analysis detected by H&E staining and Masson staining showing the hepatic pathological injury in the indicated groups (magnification, 100×, *n* = 5 mice per group). (**J**) Representative immunofluorescence images showing the Hes1 and F4/80 co-expression in liver sections of the indicated groups (magnification, 400×, *n* = 5 mice per group). Data are presented as mean ± SEM. The associated experiments were performed independently at least three times. *P* < 0.05 indicates statistical significance

We created hepatocyte-specific *NOTCH1* mutant mice and exposed them to CCl_4_ challenge because of the significance of hepatocyte NOTCH1 and hepatocyte damage during the course of hepatic inflammation (Fig. 3A, Supplementary Fig. 2A-D). In the meantime, immunoblotting and immunofluorescence assays revealed nearly nonexistent and significantly reduced expression of NOTCH1, NICD1, and Hes1 in the hepatocyte of CCl_4_-mice with hepatocyte-specific *NOTCH1* deletion in comparison to the CCl_4_/NOTCH1^f/f^ group (Fig. 3B-D). Hepatocyte-specific *NOTCH1* deletion clearly decreased the immunofluorescence pictures of liver samples from CCl_4_-induced mice, which showed significant liver injury with a decrease in HNF4A expression (Fig. 3D). Six weeks later, we discovered that these CCl_4_/NOTCH1^f/f^ mice weighed less. On the other hand, the mice in the CCl_4_/NOTCH1^CKO^ group lost much less body weight than the mice in the CCl_4_/NOTCH1^f/f^ group, but the mice in the control group lost significantly more weight than the model group (Fig. 3E). The groups did not differ significantly in terms of food intake, water intake, or liver coefficients (Fig. 3F). In contrast to the CCl_4_/NOTCH1^CKO^ model mice, the CCl_4_/NOTCH1^f/f^ groups showed a significant decrease in the total liver-to-spleen ratio (Fig. 3F). Furthermore, compared to the CCl_4_/NOTCH1^f/f^ group of mice, the NOTCH1^CKO^ mice showed reduced levels of pro-inflammatory cytokines such TNF-α, IL-6, IL-18, CCL2, and IL-1β, as well as decreased levels of AST, ALT, AKP, and GGT, suggesting meliorated inflammatory liver injury (Fig. 3G, H). Hematoxylin and eosin (H&E) and Masson’s trichrome staining were used to diagnose and confirm the pathological features of liver samples. The results showed that the CCl_4_-induced mice with hepatocyte-specific *NOTCH1* deletion had significantly lower levels of liver injury and collagen deposition than the NOTCH1^f/f^ group (Fig. 3I). Additionally, hepatic F4/80 and Hes1 levels were significantly lower in liver samples from CCl_4_/NOTCH1^CKO^ mice than in matching CCl_4_/NOTCH1^f/f^ mice, per immunofluorescence staining analysis (Fig. 3J). Next, to further confirm the expression changes of downstream NOTCH1 (NICD1) signals HES1, F4/80, α-SMA, Collagen-1, 4-HNE and one of the ferroptosis markers GPX4 when NOTCH1 is specifically absent in hepatocytes, based on the above results of hepatocytes isolation and cytoplasmic and nuclear separation components isolation, western blotting analysis confirmed that after NOTCH1 knockout in hepatocytes, the expression of HES1, a downstream expression factor of NOTCH1 (NICD1), in the nucleus was significantly reduced (Supplementary Fig. 3A). Furthermore, previous reports have confirmed that an increase in NOTCH1 expression in hepatocytes promotes inflammatory activation of Kupffer cells [20]. Therefore, we further examined the changes in the expression level of F4/80 in isolated non-parenchymal cells (containing Kupffer cells). The results showed that NOTCH1 knockout in hepatocytes could indeed significantly reduce the expression level of F4/80, leading to reduction of the inflammatory level of the liver (Supplementary Fig. 3A). Together, hepatocyte-specific NOTCH1 deficiency may slow the progression of inflammatory liver damage in mice.

### Hepatocyte-specific *NOTCH1* deficiency mitigates CCl_4_-mediated liver oxidative injury and ferroptosis pathological phenotypes

**Fig. 4:**
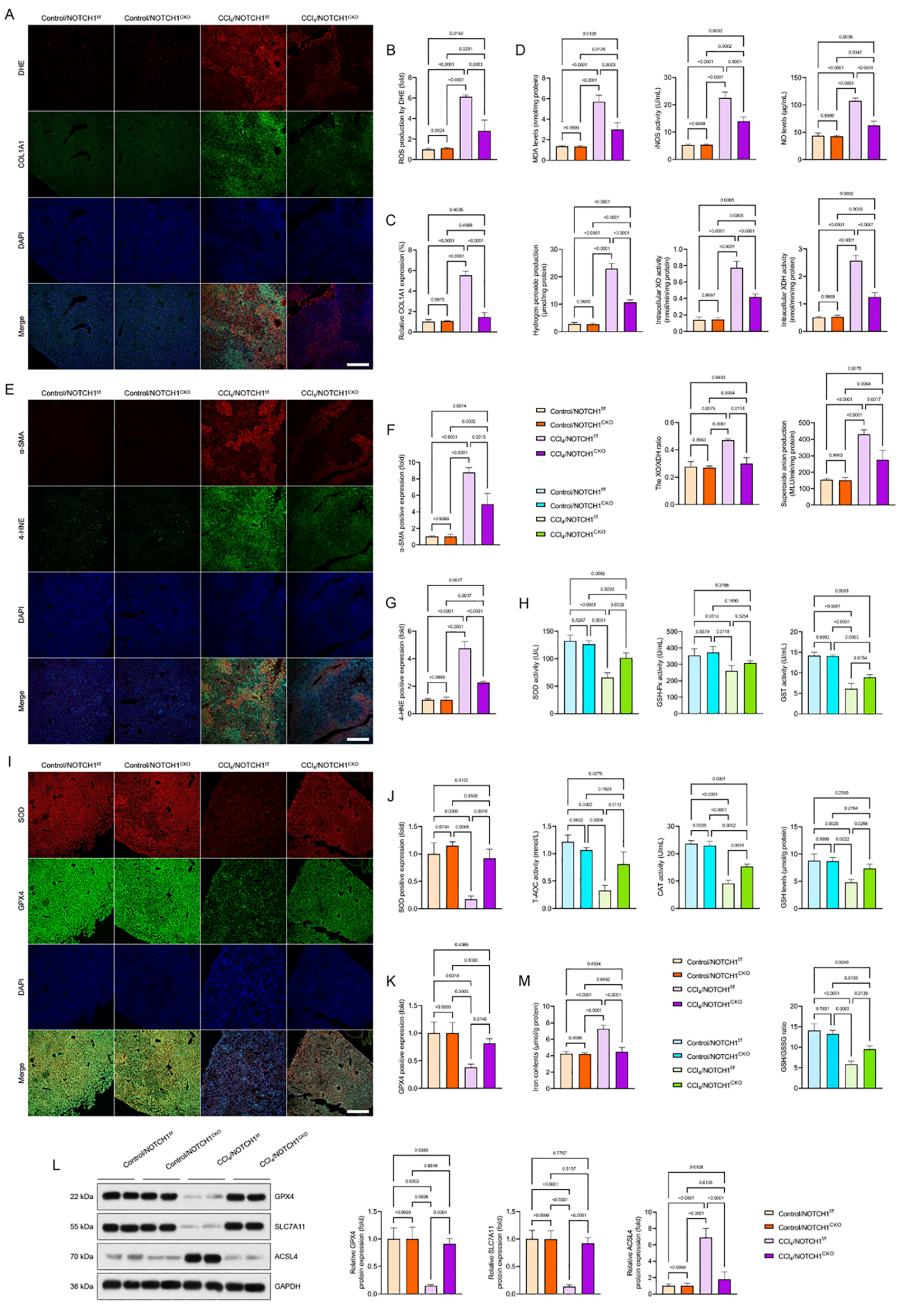
Hepatocyte-specific *NOTCH1* knockout ameliorates oxidative injury and ferroptosis in CCl_4_-treated mice. (**A-C**) Representative immunofluorescence images showing the DHE and COL1A1 levels and their quantitative analysis in liver sections of the indicated groups (magnification, 100×, *n* = 5 mice per group). (**D**) Records of hepatic MDA, iNOS, H_2_O_2_, NO, XO activity, XDH activity, XO/XDH ratio, and O_2_^−^ levels in the indicated groups (*n* = 10 mice per group). (**E-G**) Representative immunofluorescence images showing the 4-HNE and α-SMA levels and their quantitative analysis in liver sections of the indicated groups (magnification, 100×, *n* = 5 mice per group). (**H**) Records of hepatic SOD, GSH-Px, GST, T-AOC, CAT, GSH, and GSH/GSSG ration levels in the indicated groups (*n* = 10 mice per group). (**I-K**) Representative immunofluorescence images showing the SOD and GPX4 levels and their quantitative analysis in liver sections of the indicated groups (magnification, 100×, *n* = 5 mice per group). (**L**) Western blotting analysis showing the GPX4, SLC7A11 and ACSL4 protein expression in the indicated groups (*n* = 10 mice per group). (**M**) Records of iron contents in liver samples from experimental groups (*n* = 10 mice per group). Data are presented as mean ± SEM. The associated experiments were performed independently at least three times. *P* < 0.05 indicates statistical significance

Since the downregulated expression of F4/80 by IF assay (Fig. 3J) demonstrated that the inflammatory cell infiltration was significantly reduced in those CCl_4_-induced mice with *NOTCH1* deletion in hepatocytes, we are also concerned that NOTCH1-connected genes during the progression of liver injury may be closely linked to ferroptosis and oxidative stress (Supplementary Fig. 1C-F). The generation of reactive oxygen species (ROS) was then investigated in light of the potential link between NOTCH1 signaling and these biological occurrences. DHE staining of liver slides revealed that the CCl_4_-induced mice produced more ROS than the control group; however, hepatocyte-specific *NOTCH1* deletion significantly decreased the production of hepatic ROS compared to CCl_4_/NOTCH1^f/f^ mice (Fig. 4A, B). This was accompanied by a decrease in hydrogen peroxide (H_2_O_2_), nitric oxide (NO), malondialdehyde (MDA), inducible nitric oxide synthase (iNOS), XO, XDH, and superoxide anion (O_2_^−^) levels (Fig. 4A-D). The significantly reduced expression of 4-hydroxy-2-nonenal (4-HNE) and the increased levels of superoxide dismutase (SOD), glutathione-S-transferase (GST), glutathione peroxidase (GSH-Px), total antioxidant capacity (T-AOC), catalase (CAT), and glutathione (GSH) activities in comparison to the mice from the CCl_4_/NOTCH1^f/f^ group further supported the prohibitive effects of NOTCH1^CKO^ on oxidative stress in the liver (Fig. 4E-H). Notably, the livers of the CCl_4_/NOTCH1^CKO^ group of mice showed a considerable downregulation of collagen 1A1 (COL1A1) (Fig. 4A) and α-SMA (Fig. 4E) in comparison to the CCl_4_/NOTCH1^f/f^ mice. In the animals given CCl_4_, *NOTCH1* ablation resulted in a marked reduction in the expression of genes linked to fibrosis and the inflammatory response (Supplementary Fig. 3B). Furthermore, it was discovered that in CCl_4_-induced mice, the expression levels of ferroptosis hallmarks glutathione peroxidase 4 (GPX4), SOD, and solute carrier family 7-member 11 (SLC7A11) were significantly restored when *NOTCH1* was absent, while Acyl-CoA synthetase long-chain family member 4 (ACSL4) was diminished (Fig. 4I-L). Additionally, the liver of CCl_4_-induced mice had lower GSH levels and a lower GSH/oxidized GSH (GSSG) ratio than the control group, but it was greatly restored in NOTCH1^CKO^ group, and the iron concentrations decreased (Fig. 4M). Also, after NOTCH1 knockout, the abundance of α-SMA, Collagen-1 and 4-HNE expression in hepatocytes significantly decreased, while the level of GPX4 significantly increased (Supplementary Fig. 3A). These results are consistent with our immunofluorescence results. This further confirms that the specific deletion of NOTCH1 in hepatocytes can indeed positively affect the related pathological phenotypic changes.

These NOTCH1^CKO^ mice showed increased expression of ferroptosis inhibitors GPX4, SLC7A11, and solute carrier 3A2 (SLC3A2), as well as antioxidants like HO-1, SOD1, NAD(P)H: quinine oxidoreductase 1 (NQO1), and glutamate cysteine ligase catalytic subunit (GCLC) in their livers by qPCR analysis when compared to the CCl_4_/NOTCH1^f/f^ group (Supplementary Fig. 3B). We next used the isolated liver samples from CCl_4_/NOTCH1^CKO^ mice in comparison to the CCl_4_/NOTCH1^f/f^ group to do RNA-Seq in order to fully investigate the functional impacts of NOTCH1 in the context of inflammatory liver injury in vivo. 161 DEGs with 83 upregulated genes, such as NFE2L2 (NRF2), GPX4, GCLC, and SOD1, and other downregulated genes, such as HES1, HEY1, CCN2, CD36, and STAT3, were displayed in the volcano plot (Supplementary Fig. 3C). Gene Ontology (GO), Kyoto Encyclopedia of Genes and Genomes (KEGG), and gene set enrichment analysis (GSEA) enrichment analysis all supported the paired lollipop chart based on DEGs, which showed that NOTCH1^CKO^ downregulated the genes involved in inflammation and profibrosis (Supplementary Fig. 3D-F). These findings imply that NOTCH1 may favorably mediate hepatocyte injury, fibrosis, and inflammation.

To further evaluate the molecular role of NOTCH1 in controlling important indicators of oxidative stress and ferroptosis with or without 1% CCl_4_ challenge, we then developed an in vitro experimental model using adenovirus (Ad)-shRNA-induced NOTCH1 knockdown (Adsh*NOTCH1*) constructs in human THLE2 cells, with AdshRNA acting as a control. In THLE2 cells transfected with Adsh*NOTCH1*, CCl_4_-induced increased expression levels of NICD1 and KEAP1 were significantly reduced, whereas NRF2 expression levels were noticeably elevated (Supplementary Fig. 4A). As anticipated, 10 h of CCl_4_ incubation resulted in a marked decrease in cell viability, which was notably suppressed by NOTCH1 knockdown in THLE2 cells (Supplementary Fig. 4B). GSH levels, the GSH/GSSG ratio index, and adenosine triphosphate (ATP) levels were increased in THLE2 cells infected with Adsh*NOTCH1* under CCl_4_ conditions, but MDA levels, Fe^2+^ contents, and relative ferric ions were significantly downregulated (Supplementary Fig. 4C-H). When compared to the control group, CCl_4_ significantly increased cellular and mitochondrial ROS generation and α-SMA expression; however, NOTCH1 knockdown resulted in a decrease in both processes (Supplementary Fig. 4I, J). Furthermore, following CCl_4_ challenge, Mito-Tracker staining demonstrated the enhanced mitochondrial structure of THLE2 cells lacking NOTCH1 (Supplementary Fig. 4K). When NOTCH1 was knocked down in THLE2 cells, BODIPY-C11 labeling demonstrated that the lipid peroxidation induced by CCl_4_ was significantly eliminated (Supplementary Fig. 4L). Lastly, the effects of Adsh*NOTCH1* on the enhancement of GPX4 and SLC7A11 expression levels in CCl_4_-treated THLE2 cells were validated by western blotting results (Supplementary Fig. 4M). Thus, these findings show how NOTCH1 is linked to ferroptosis and oxidative stress in response to CCl_4_ challenge. By preventing oxidative damage and ferroptotic hepatocyte cell death, NOTCH1 suppression may help treat liver injury.

### Hepatocyte-specific *NOTCH1* overexpression facilitates CCl_4_-mediated liver inflammation, oxidative injury and ferroptosis pathological phenotypes

**Fig. 5:**
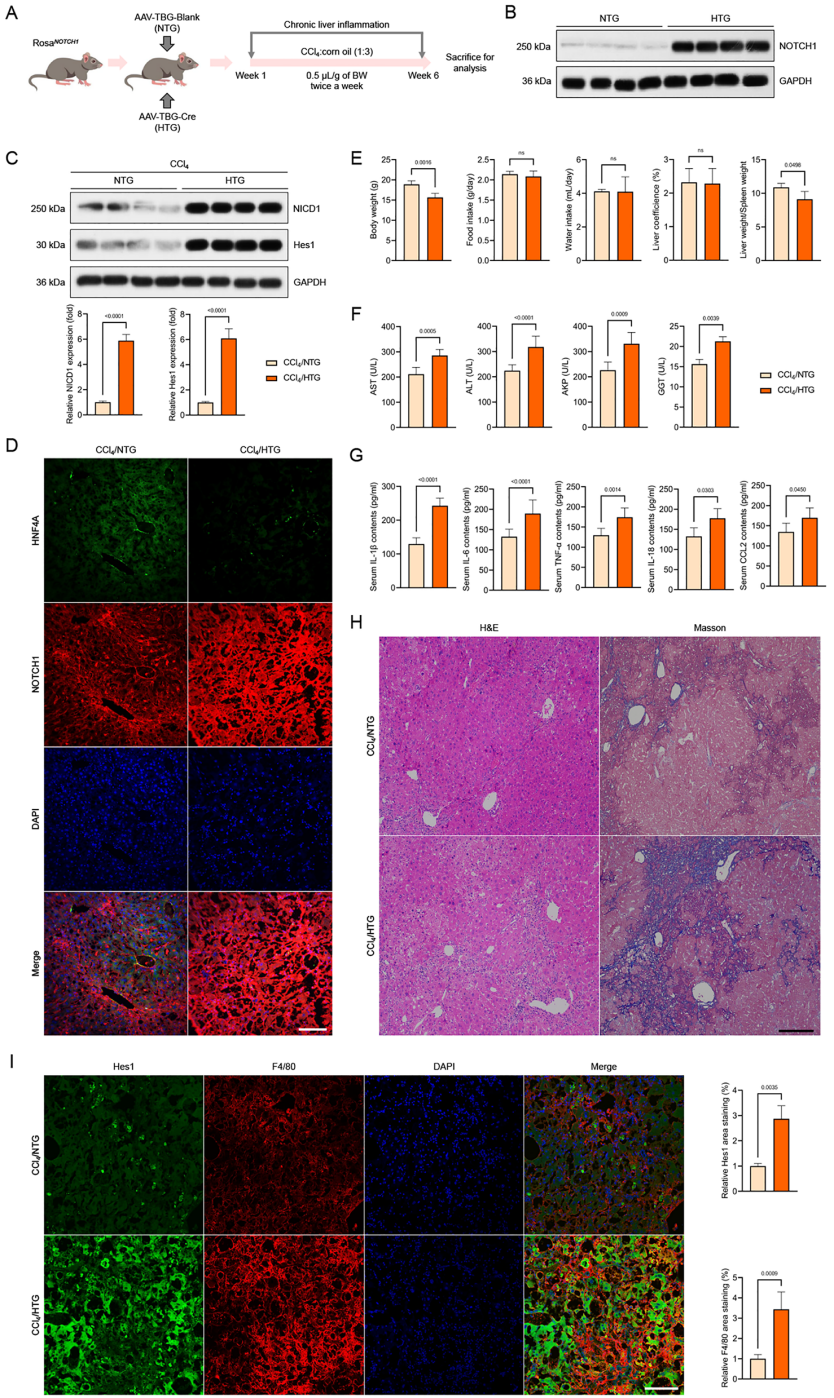
Hepatocyte-specific *NOTCH1* overexpression promotes hepatocellular inflammation injury in CCl_4_-treated mice. (**A**,** B**) Schematic of experimental procedures and quantitative analysis examining the effects of hepatocyte-specific *NOTCH1* overexpression (HTG) using AAV-TBG-Cre-injected Rosa^*NOTCH1*^ mice on CCl_4_-treated mice; AAV-TBG-Blank-injected Rosa^*NOTCH1*^ mice (NTG) served as the control (*n* = 4 mice per group). (**C**) Western blotting analysis showing the NICD1 and Hes1 expression in CCl_4_/NTG and CCl_4_/HTG groups (*n* = 4 mice per group). (**D**) Representative immunofluorescence images of NOTCH1 and HNF4A expression in liver samples of indicated groups (magnification, 100×, *n* = 10 samples). Records of body weight, food intake, water intake, liver coefficience, liver-to-spleen ratio (**E**), serum AST, ALT, AKP & GGT contents (**F**), serum pro-inflammatory cytokines TNF-α, IL-6, IL-18, CCL2, and IL-1β levels (**G**) in CCl_4_/NTG and CCl_4_/HTG groups (*n* = 10 mice per group). (**H**) Representative liver histological analysis detected by H&E staining and Masson staining showing the hepatic pathological injury in the indicated groups (magnification, 100×, *n* = 5 mice per group). (**I**) Representative immunofluorescence images of Hes1 and F4/80 expression in liver samples of indicated groups (magnification, 100×, *n* = 10 samples). Data are presented as mean ± SEM. The associated experiments were performed independently at least three times. *P* < 0.05 indicates statistical significance

**Fig. 6:**
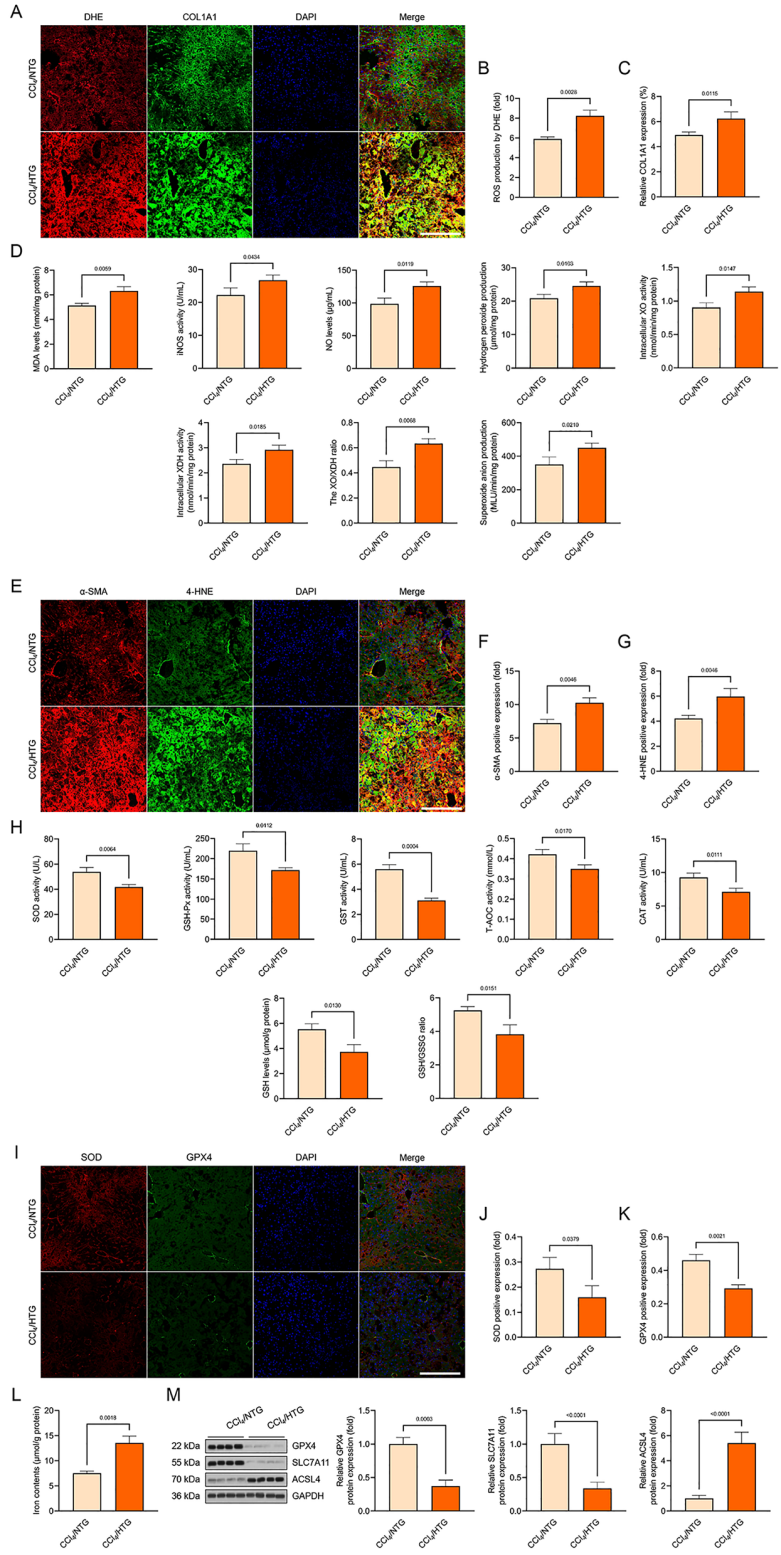
Hepatocyte-specific *NOTCH1* overexpression facilitates oxidative injury and ferroptosis in CCl_4_-treated mice. (**A-C**) Representative immunofluorescence images showing the DHE and COL1A1 levels and their quantitative analysis in liver sections of the indicated groups (magnification, 100×, *n* = 5 mice per group). (**D**) Records of hepatic MDA, iNOS, H_2_O_2_, NO, XO activity, XDH activity, XO/XDH ratio, and O_2_^−^ levels in the indicated groups (*n* = 10 mice per group). (**E-G**) Representative immunofluorescence images showing the 4-HNE and α-SMA levels and their quantitative analysis in liver sections of the indicated groups (magnification, 100×, *n* = 5 mice per group). (**H**) Records of hepatic SOD, GSH-Px, GST, T-AOC, CAT, GSH, and GSH/GSSG ration levels in the indicated groups (*n* = 10 mice per group). (**I-K**) Representative immunofluorescence images showing the SOD and GPX4 levels and their quantitative analysis in liver sections of the indicated groups (magnification, 100×, *n* = 5 mice per group). (**L**) Records of iron contents in liver samples from experimental groups (*n* = 10 mice per group). (**M**) Western blotting analysis showing the GPX4, SLC7A11 and ACSL4 protein expression in the indicated groups (*n* = 10 mice per group). Data are presented as mean ± SEM. The associated experiments were performed independently at least three times. *P* < 0.05 indicates statistical significance

We further confirmed whether overexpression of NOTCH1 exacerbated inflammatory liver injury and oxidative stress using AAV8-TBG-Cre-injected Rosa^*NOTCH1*^ mice (HTG) during CCl_4_ challenge, as NOTCH1 deficiency can dramatically reduce hepatocyte inflammation and oxidative damage (Fig. 5A-D). As anticipated, the HTG mice’s body weight loss in response to CCl_4_ administration was much more than that of the NTG mice, in contrast to the CCl_4_/NOTCH1^CKO^ animals and CCl_4_-treated AAV8-TBG-Blank (NTG)-injected mice (Fig. 5E). There was no discernible difference in the two groups’ liver coefficients, food consumption, or water intake. In contrast to the CCl_4_/HTG model mice, the CCl_4_/NTG groups showed a significant increase in the total liver-to-spleen ratio (Fig. 5E). Additionally, compared to the CCl_4_/NTG group of mice, the CCl_4_/HTG mice showed higher levels of pro-inflammatory cytokines like TNF-α, IL-6, IL-18, CCL2, and IL-1β, as well as higher levels of AST, ALT, AKP, and GGT, suggesting accelerated inflammatory liver injury (Fig. 5F, G). By using H&E and Masson’s trichrome staining to diagnose and validate the pathological characteristics of liver tissues, it was found that CCl_4_-induced mice with hepatocyte-specific *NOTCH1* overexpression had significantly higher levels of liver injury and collagen deposition than the NTG group (Fig. 5H). Additionally, hepatic F4/80 and Hes1 levels were significantly higher in liver samples from CCl_4_/HTG mice than in matching CCl_4_/NTG mice, per immunofluorescence staining analysis (Fig. 5I). Furthermore, compared to the NTG group, the CCl_4_-induced HTG mice produced more ROS and had greater COL1A1 contents in their liver samples (Fig. 6A-C). They also had higher levels of MDA, iNOS, NO, H_2_O_2_, XO, XDH, and O_2_^−^ (Fig. 6D). As demonstrated by the markedly increased expression of 4-HNE and α-SMA and the downregulated SOD, GST, GSH-Px, T-AOC, CAT, and GSH activities in comparison to those mice from the CCl_4_/NTG group, immunofluorescence analysis further supported the facilitated effects of NOTCH1 overexpression on oxidative stress and liver profibrosis (Fig. 6E-H). Meanwhile, we further verified that when NOTCH1 was specifically overexpressed in hepatocytes, the changes in the above indicators were consistent with our immunofluorescence results. The specific overexpression of NOTCH1 in hepatocytes could significantly increase the expression abundance of Hes1 in the hepatocyte nucleus, and enhance the expression level of F4/80 in isolated nonparenchymal cells. The abundance of a-SMA, Collagen 1, and 4-HNE was increased, and the expression level of GPX4 was decreased (Supplementary Fig. 5A).

However, to further evaluate the molecular role of NOTCH1 in controlling important indicators of oxidative stress and ferroptosis with 1% CCl_4_ challenge, we later developed an in vitro experimental model using adenovirus (Ad)-induced NOTCH1 overexpression (Ad*NOTCH1*) constructs in THLE2 cells, with the AdControl acting as control. In THLE2 cells transfected with Ad*NOTCH1*, CCl_4_-induced expression levels of NICD1 and KEAP1 were significantly increased, while NRF2, GPX4, and SLC7A11 expression levels were significantly downregulated (Supplementary Fig. 5B). As anticipated, NRF2 activity was significantly reduced after 10 h of CCl_4_ treatment, primarily due to NOTCH1 overexpression in THLE2 cells (Supplementary Fig. 5C). When compared to the control group, CCl_4_ significantly increased the formation of ROS in cells and mitochondria as well as the expression of Mito-SOX (Supplementary Fig. 5D, E). When NOTCH1 was overexpressed in THLE2 cells, BODIPY-C11 staining verified that the lipid peroxidation induced by CCl_4_ was significantly amplified (Supplementary Fig. 5F). Additionally, in THLE2 cells infected with Ad*NOTCH1*, GSH levels, the GSH/GSSG ratio index, and ATP levels were downregulated, but MDA levels and Fe^2+^ contents were significantly increased (Supplementary Fig. 5G). The CCl_4_/Ad*NOTCH1* animals showed higher levels of genes linked to inflammation but lower levels of genes related to ferroptosis inhibitors and antioxidants when compared to the CCl_4_/AdControl group (Supplementary Fig. 5H). These results showed a correlation between the degree and progression of liver injury and aberrantly elevated NOTCH1 levels.

### NOTCH1 recruits KEAP1 to facilitate ROS generation and ferroptosis by promoting NRF2 degradation

**Fig. 7:**
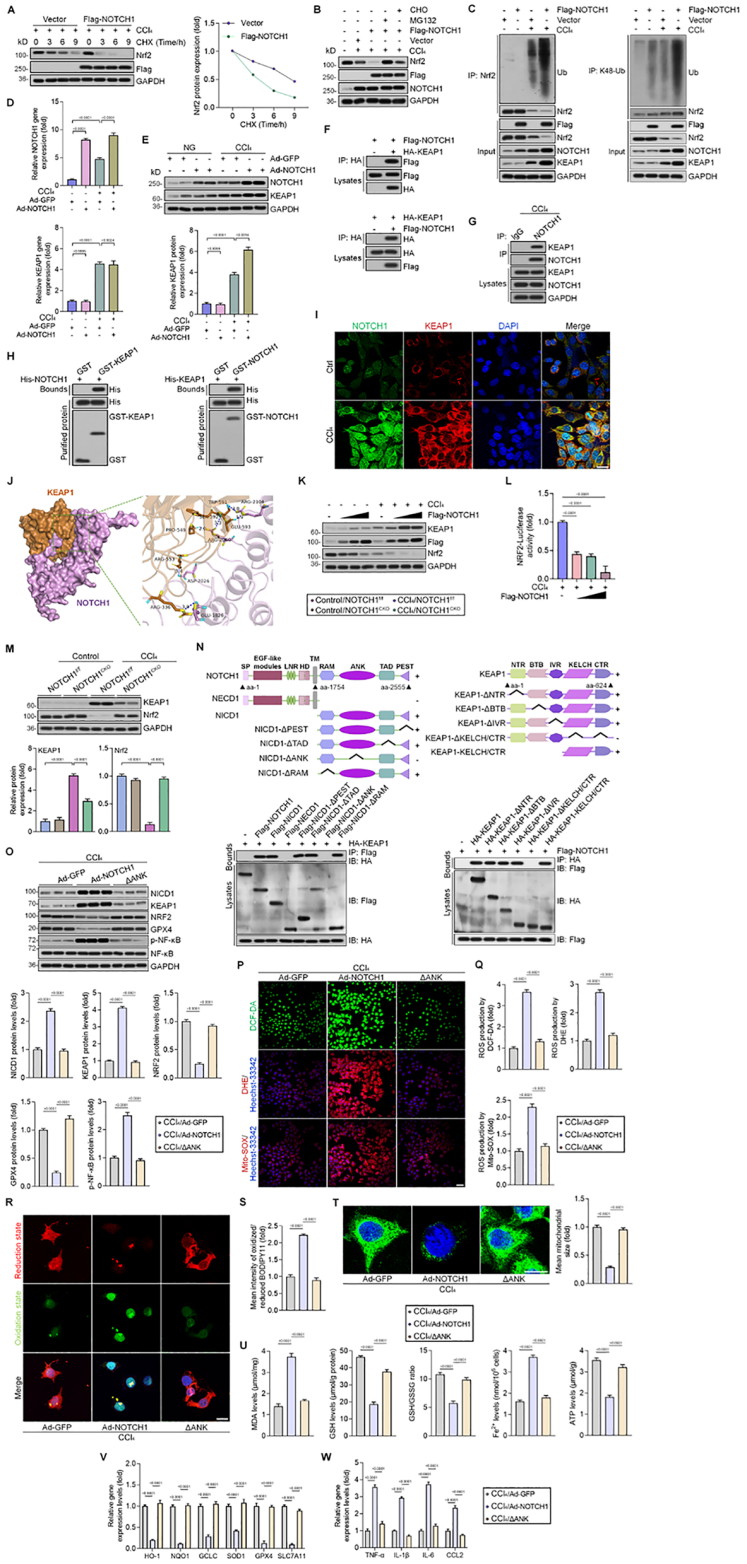
NOTCH1 interacts with and recruits KEAP1 in hepatocytes, leading to NRF2 polyubiquitination degradation under CCl_4_ challenge. (**A**) THLE2 cells after transfection with Flag-NOTCH1 or the empty Vector were incubated with CCl_4_ for 24 h simultaneously in the presence of the protein synthesis inhibitor cycloheximide (CHX; 50 µg/ml) for the indicated times (0 h, 3 h, 6 h, 9 h). Relative protein expression levels for NRF2 in transfected THLE2 cells after time-course treatment were quantified (*n* = 4 per group). (**B**) Immunoblotting detection of Flag-NOTCH1 transfected THLE2 cells with/without CCl_4_ (24 h), MG132 (20 µM, 12 h) and CHO (20 µM, 12 h) treatment (*n* = 4 per group). (**C**) Left, the human THLE2 cells were transfected with the indicated plasmids. Anti-Nrf2 immunoprecipitates were analyzed by immunoblotting with anti-Ub antibody for the examination of ubiquitin-conjugated Nrf2. Right, the human THLE2 cells were transfected with the indicated plasmids. Anti-K48-Ub immunoprecipitates were analyzed by immunoblotting with anti-Ub antibody for the examination of ubiquitin-conjugated Nrf2. (**D**) The human THLE2 cells transfected with Ad*NOTCH1* or AdGFP were incubated with CCl_4_ for 24 h, and were then collected for qPCR analysis of NOTCH1 and KEAP1 (*n* = 5 per group). (**E**) Western blot analysis for NOTCH1 and KEAP1 protein expression levels in 24 h of CCl_4_-treated THLE2 cells with or without Ad*NOTCH1* transfection (*n* = 4 per group). (**F**) Immunoprecipitation and western blot analysis indicating the binding of KEAP1 to NOTCH1 in human THLE2 cells transfected with Flag-NOTCH1 and HA-tagged KEAP1 (HA-KEAP1) under CCl_4_ exposure. (**G**) Immunoprecipitation and immunoblotting assay showing the binding of KEAP1 to NOTCH1 in the liver samples of CCl_4_-treated mice; the IgG was served as a control. (**H**) Representative immunoblotting bands for GST precipitation showing NOTCH1-KEAP1 direct binding by treating purified NOTCH1-His with purified KEAP1-HA-GST or by treating KEAP1-His with purified NOTCH1-HA-GST in THLE2 cells. (**I**) Representative IF images showing NOTCH1 (green) and KEAP1 (red) in THLE2 cells challenged with/without CCl_4_ for 24 h (*n* = 5 independent biological replicates with 8 images per group). (**J**) Molecular docking analysis between NOTCH1 and KEAP1 protein. (**K**) Representative western blot of KEAP1 and NRF2 in THLE2 cells transfected with varying amounts of Flag-NOTCH1 with or without CCl_4_ incubation for 24 h. (**L**) Luciferase assay of the fluorescence intensity of THLE2 cells transfected with increasing counts of Flag-NOTCH1 plasmids in response to HG treatment for 24 h (*n* = 6 per group). (**M**) Western blot results for KEAP1 and NRF2 in the isolated hepatocytes from the shown groups (*n* = 4 per group). (**N**) Schematic indicating full-length and truncated NOTCH1 (upper, left) and KEAP1 (upper, right) with representative Co-IP results (bottom) for the mapping analysis of the domains responsible for the NOTCH1-KEAP1 interaction in human THLE2 cells. (**O**) Western blots of NICD1, KEAP1, NRF2, GPX4, and p-NF-κB in human THLE2 cells transfected with AdGFP, Ad*NOTCH1* (WT) or the ∆ANK NOTCH1/NICD1 variant at 24 h after CCl_4_ treatment (*n* = 3 per group). (**P**) DCF-DA staining, DHE staining, and Mito-SOX staining, and (**Q**) quantification for ROS production by respective staining were performed in human THLE2 cells transfected with AdGFP, Ad*NOTCH1* (WT) or the ∆ANK NOTCH1/NICD1 variant in response to CCl_4_ treatment for 24 h. (**R**) Lipid peroxidation was examined by C11-BODIPY in human THLE2 cells with AdGFP, Ad*NOTCH1* (WT) or the ∆ANK NOTCH1/NICD1 variant under CCl_4_ treatment for 24 h; red fluorescence represents the reduction form, while green fluorescence represents the oxidized form. (**S**) Mean intensity for the ratio of the oxidized form to reduced form was quantified related to C11-BODIPY staining (*n* = 5 per group). (**T**) Mito-Tracker was used to examine the mitochondrial structure changes of THLE2 cells transfected with AdGFP, Ad*NOTCH1* (WT) or the ∆ANK NOTCH1/NICD1 variant under CCl_4_ treatment for 24 h, and the mean mitochondrial size was then quantified (*n* = 6 per group). (**U**) Measurements for MDA levels, GSH contents, GSH/GSSG ratio, Fe^2+^ levels and ATP levels in 24 h of CCl_4_-treated THLE2 cells with AdGFP, Ad*NOTCH1* (WT) or the ∆ANK NOTCH1/NICD1 variant transfection (*n* = 6 per group). (**V**) qPCR analysis for the mRNA expression levels of genes involved in oxidative stress and ferroptosis as shown in THLE2 cells transfected with AdGFP, Ad*NOTCH1* (WT) or the ∆ANK NOTCH1/NICD1 variant after CCl_4_ incubation for 24 h (*n* = 4 in each group). (**W**) The mRNA expression levels of inflammatory genes were evaluated by qPCR in 24 h CCl_4_-incubated THLE2 cells transfected with AdGFP, Ad*NOTCH1* (WT) or the ∆ANK NOTCH1/NICD1 variant (*n* = 4 in each group). Data are presented as mean ± SEM. *P* < 0.05 indicates statistical significance. Two-tailed unpaired *t*-test was used to determine the *p*-values in (**A**), (**E**) and (**F**); Statistical comparisons in (**L**), (**M**), (**O**), (**Q**), (**S**) to (**W**) were performed using one-way ANOVA with a Tukey post-hoc analysis; the results in (**C**), (**G**) to (**I**), (**K**), and (**N**) were obtained from three independent experiments

Considering the possible regulatory effects of NOTCH1 on oxidative stress and ferroptosis in the progression of liver injury, mechanistical studies were then performed to gain a deeper understanding. As the expression and stability of NRF2 play a key role in defending against oxidative damage, its protein expression and degradation were investigated by adding cycloheximide (CHX) in CCl_4_-stimulated human THLE2 cells transfected with Flag-tagged NOTCH1 plasmids, and the empty vector served as control. Suppression of protein synthesis by CHX in transfected hepatocytes confirmed that NRF2 was less stable when expressed in the presence of Flag-NOTCH1 (Fig. 7A). Given to the post-transcriptional regulation of NOTCH1 on NRF2 under CCl_4_ challenge, we then treated THLE2 cells under CCl_4_ with the proteasome inhibitor MG132 or the lysosome inhibitor chloroquine (CHO). We found that MG132, but not CHO, blocked the decrease of NRF2 protein in response to CCl_4_ treatment (Fig. 7B), indicating that NRF2 might be degraded via a ubiquitin-proteasome pathway. Indeed, we observed hyperubiquitination of NRF2 in CCl_4_-induced THLE2 cells, particularly in those THLE2 cells with Flag-NOTCH1 transfection (Fig. 7C). However, NOTCH1 itself has no ubiquitination enzyme activity. Herein, we hypothesized that NOTCH1 may interact with other protein to induce NRF2 ubiquitination and degradation under stresses. KEAP1 is an important cellular protein that serves primarily as a substrate recognition subunit of E3 ubiquitin ligase with ubiquitination enzyme activity, and is able to recognize a variety of substrate proteins, such as NRF2, which plays an important role in the process of cellular antioxidant response and metabolic regulation. To detect whether KEAP1 was involved in the process of oxidative stress and ferroptosis mediated by NOTCH1, we generated THLE2 cells with adenovirus-mediated NOTCH1 overexpression (Ad*NOTCH1*) and subjected them to CCl_4_ administration. We noted that promoting NOTCH1 further enhanced the protein expression levels of KEAP1 under CCl_4_ treatment, while showed no significant influences on the transcriptional expression levels of KEAP1 (Fig. 7D, E). A co-immunoprecipitation (Co-IP) analysis showed an interaction between NOTCH1 and KEAP1 (Fig. 7F). The interaction between NOTCH1 and KEAP1 was further identified in the livers of CCl_4_-induced mice (Fig. 7G). The glutathione *S*-transferase (GST) precipitation analysis revealed a direct interaction between NOTCH1 and KEAP1 (Fig. 7H). Double IF staining showed that NOTCH1 was obviously colocalized with KEAP1 in THLE2 cells particularly in response to CCl_4_ treatment (Fig. 7I). Molecular docking assay further indicated that GLU-1826, ASP-2026, ARG-2060, and ARG-2104 of NOTCH1 formed hydrogen bonds with ARG-336, ARG-553, PRO-549, SER-592, GLU-593, and TRP-591 of KEAP1, respectively. The length of hydrogen bond was 3.0Å, 3.4Å, 2.0Å, 3.3Å, 1.9Å, 3.0Å, respectively. The binding energy was − 264.33 kcal/mol, indicating a very strong interaction between the two proteins (Fig. 7J). Immunoblotting analysis showed that the induced action of NOTCH1 on KEAP1 was dose-dependent under CCl_4_ treatment, accompanied with downregulated NRF2 protein expression levels (Fig. 7K). Consistently, the NRF2 luciferase activity was dose-dependently decreased by Flag-NOTCH1 in CCl_4_-treated THLE2 cells (Fig. 7L). As expected, CCl_4_-induced mice exerted higher protein expression levels of KEAP1 in the isolated hepatocytes, but were markedly reduced upon NOTCH1 deficiency; however, NRF2 showed the opposite expression trends (Fig. 7M). Calculation of human samples also indicated stronger expression of KEAP1 in liver specimens of NASH-HCC patients than that of the control group (Supplementary Fig. 6A and B). Of note, a positive correlation between NICD1 and KEAP1 protein expression levels were detected in NASH-HCC patients, accompanied by a negative correlation between NRF2 with KEAP1 and NICD1 (Supplementary Fig. 6C). Immunofluorescence staining also showed a colocalization of NOTCH1 and KEAP1 in liver samples of NASH-HCC and HBV patients (Supplementary Fig. 6D).

In addition, a molecular mapping assay subsequently revealed that the NICD1-ANK binding domain of NOTCH1 was responsible for its direct interaction with the KELCH-CTR domain of KEAP1 (Fig. 7N). We then found that overexpression of human NOTCH1 drastically inhibited NRF2, GPX4 and SLC7A11 expression levels, but enhanced KEAP1 and phosphorylated nuclear factor-κB (p-NF-κB) in CCl_4_-stimulated THLE2 cells, whereas this effect was completely absent in cells expressing the NOTCH1/ΔANK mutant (Fig. 7O). Hepatocytes with the ΔANK mutation consistently showed reduced levels of excessive cellular and mitochondrial ROS production brought on by CCl_4_ (Fig. 7P, Q). Lipid peroxidation was accelerated in Ad*NOTCH1* THLE2 cells in response to CCl_4_ treatment, but was suppressed when ΔANK was mutant by the BODIPY-C11 staining (Fig. 7R, S). Mitochondrial integrity whittled by CCl_4_ was facilitated upon NOTCH1 overexpression, whereas being rescued in ΔANK-mutant cells (Fig. 7T). These effects mediated by ΔANK mutation were accompanied with decreased MDA and Fe^2+^ levels, but increased GSH contents, GSH/GSSG ratio and ATP levels (Fig. 7U). As expected, ΔANK mutation strongly restored the expression levels of genes responsible for anti-oxidative stress and -ferroptosis, and lessened the mRNA levels of inflammatory factors (Fig. 7V, W). To confirm the potential of ANK domain in the mediation of KEAP1/NRF2 signaling under CCl_4_ challenge to regulate ROS production and ferroptosis, we then overexpressed ANK by infecting with the constructed AdANK in THLE2 cells. Comparing with the Adsh*NOTCH1* group, promoting ANK domain facilitated KEAP1 protein expression levels, but inhibited NRF2, GPX4 and SLC7A11 in CCl_4_-incubated THLE2 cells, along with weakened NRF2 luciferase activity (Supplementary Fig. 7A and B). ROS production, lipid peroxidation and mitochondrial structure improved by Adsh*NOTCH1* were completely diminished in CCl_4_-treated THLE2 cells transfected with AdANK (Supplementary Fig. 7C-E). Additionally, AdANK dramatically recovered MDA and Fe^2+^ levels in CCl_4_-stimulated THLE2 cells, while cut down GSH, GSH/GSSG ratio and ATP contents (Supplementary Fig. 7F). The transcription levels of anti-oxidant and -ferroptotic genes were also abolished by AdANK, but the proinflammatory factors were powerfully enhanced (Supplementary Fig. 7G and H). In short, these data suggest that NOTCH1 facilitates NRF2 degradation by recruiting and interacting with KEAP1 to promote ROS generation and ferroptosis in hepatocytes.

### KEAP1 is required for NOTCH1 suppression-mediated mitigation of liver injury

**Fig. 8:**
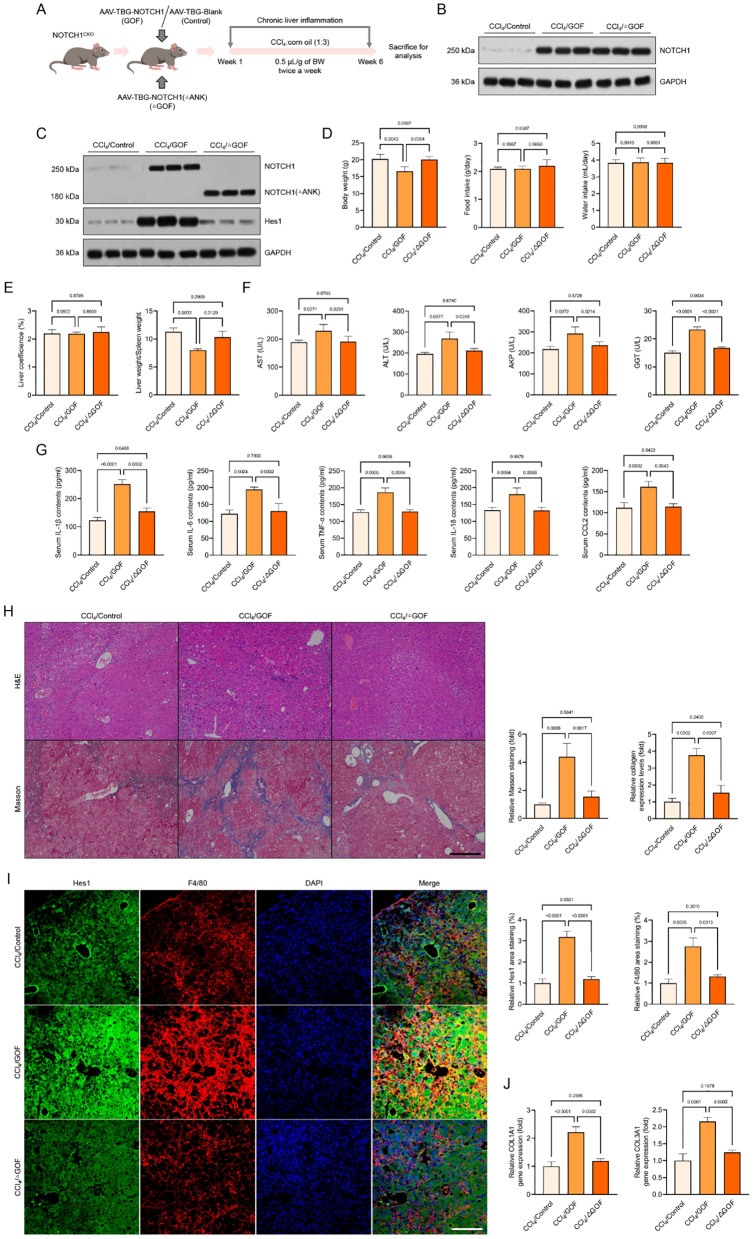
Mutational *NOTCH1* (△ANK) cannot effectively promotes CCl_4_-induced liver injury. (**A**) Schematic diagram of adeno-associated virus (serotype 8)-TBG-Cre (AAV-TBG-Cre)-mediated *NOTCH1* restoration (GOF) or *NOTCH1* (△ANK) restoration (△GOF) in liver of CCl_4_-fed NOTCH1^CKO^ mice. The AAV-TBG-Blank was used as control (Control). (**B**,** C**) Western blotting analysis showing the NOTCH1, NOTCH1(△ANK), and Hes1 expression in the indicated mice groups (*n* = 5 mice per group). (**D-G**) Records of body weight, food intake, water intake, liver coefficience, liver-to-spleen ratio, serum AST, ALT, AKP, GGT, pro-inflammatory cytokines TNF-α, IL-6, IL-18, CCL2, and IL-1β levels in CCl_4_/Control, CCl_4_/GOF and CCl_4_/△GOF groups (*n* = 10 mice per group). (**H**) Representative liver histological analysis detected by H&E staining and Masson staining showing the hepatic pathological injury in the indicated groups (magnification, 100×, *n* = 5 mice per group). (**I**) Representative immunofluorescence images of Hes1 and F4/80 expression in liver samples of indicated groups (magnification, 100×, *n* = 10 samples). Data are presented as mean ± SEM. The associated experiments were performed independently at least three times. *P* < 0.05 indicates statistical significance

Having the potent promoted effects of NOTCH1-KEAP1 signaling on liver injury progression and its associated pathological processes, the above results forced us to study the molecular mechanisms of NOTCH1 and its inherent function. To further confirm whether NOTCH1 with ANK domain mutants are required for the functional role of NOTCH1-KEAP1 promote NASH progression, we then subjected AAV-TBG-NOTCH1(GOF), AAV-TBG-NOTCH1 (△ANK)(△GOF), or AAV-TBG-Blank(Control) to 6-weeks CCl_4_-treated NOTCH1^CKO^ mice in vivo (Fig. 8A-C). Consistent with results in vitro in Supplementary Fig. 7., unsurprisingly, except for the mice injected with AAV-TBG-NOTCH1, groups injected with AAV-TBG-NOTCH1 (△ANK), ANK mutants, were not capable of promoting liver inflammation response, fibrosis development and hepatocellular injury after CCl_4_ challenge compared to those of corresponding controls (Fig. 8D-J).

Given the potent accelerated effects of NOTCH1-KEAP1 axis on liver injury development, on the basis of confirmation of KEAP1 as a potential target and substrate of NOTCH1, we next established hepatocyte-specific NOTCH1 and KEAP1 dual deficiency mice (*NOTCH1*-*KEAP1*^DKO^), as described in Methods section. Next, the primary hepatocytes with NOTCH1 and KEAP1 dual knockout were isolated and then transfected with Ad*NOTCH1*, Ad*KEAP1* or Ad*NOTCH1* + Ad*KEAP1* vectors, respectively, followed by CCl_4_ challenge for 10 h (Supplementary Fig. 8A, B). As expected, compared with Ad*NOTCH1* transfection alone, the absence of KEAP1 greatly hindered the CCL_4_-induced liver injury phenotype. In contrast, when Ad*NOTCH1* and Ad*KEAP1* were transfected simultaneously, this blocking effect was significantly weakened. Importantly, all the inflammatory liver damage pathological symptoms that were promoted by NOTCH1-KEAP1 restoration including increased oxidative stress, mitochondria damage, inflammatory response, and collagen production were also significantly observed in Ad*NOTCH1* + Ad*KEAP1*-transfected primary hepatocytes, compared with Ad*NOTCH1* transfected hepatocytes alone (Supplementary Fig. 8C-M). Thus, the effects of NOTCH1 on exacerbation of liver injury is largely rely on its ability to positively regulate KEAP1.

### Forced activation of NOTCH1-KEAP1 accelerates hepatocellular carcinoma (HCC) progression

Given that NOTCH1 is the cause of hepatic carcinoma and that virus hepatitis (HBV, HCV) and NASH are important risk factors for cirrhosis and hepatocellular carcinoma (HCC) in clinical manifestation, we investigated whether NOTCH1 intervention could increase the incidence of HCC by regulating NRF2 signaling and targeting KEAP1 signaling. Based on paired and non-paired samples from the TCGA and ICGC databases, the pan-caner analysis inevitably revealed elevated NOTCH1 and KEAP1 expression in the progression of liver cancer (LIHC) (Supplementary Fig. 9A-C). Additionally, we evaluated NOTCH1 and KEAP1’s predictive relevance in these liver cancer tissue microarray (TMA) datasets. Notably, in the TCGA LIHC and ICGC LIHC databases, patients with high levels of NOTCH1 and KEAP1 had a much lower overall survival than patients with low levels of NOTCH1 and KEAP1 (Supplementary Fig. 9D). Significant LIHC immune infiltration brought on by aberrant NOTCH1 and KEAP1 expression further supported these findings (Supplementary Fig. 9E, F).

**Fig. 9:**
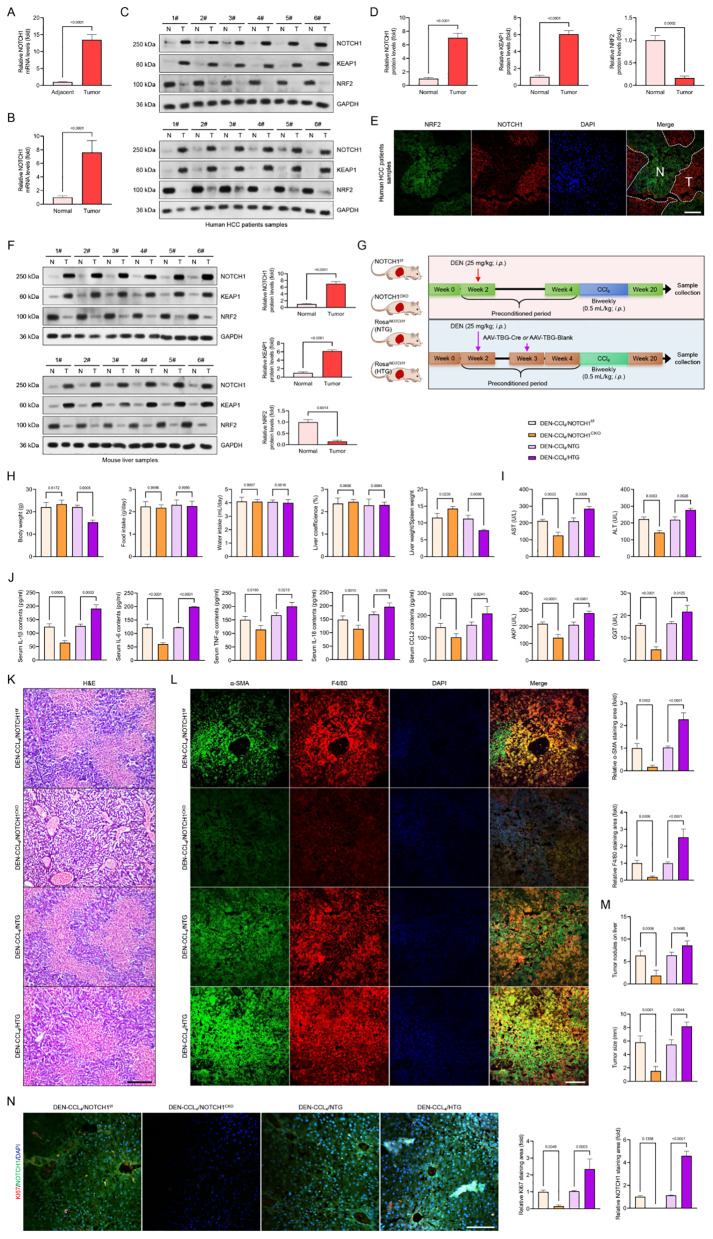
NOTCH1 promotes progression of liver inflammation-associated HCC. (**A**) Results for NOTCH1 mRNA expression levels of adjacent tissue and 12 individual paired HCC human patients tumor samples (*n* = 12 samples per group). Relative mRNA expression (**B**) and western blotting analysis (**C**,** D**) of NOTCH1 in 12 individual paired HCC human patients tumor samples and normal samples (*n* = 12 samples per group). (**E**) Representative immunofluorescence images showing the NRF2 and NOTCH1 co-expression in liver samples of human HCC patients (*n* = 6 samples per group; N, normal; T, tumor). (**F**) Western blotting analysis showing the NOTCH1, KEAP1, and NRF2 protein expression in livers of HCC mouse models (*n* = 12 samples per group). (**G**) Scheme for the experimental design on DEN-injected and CCl_4_-administrated HCC mouse models with hepatocyte-specific *NOTCH1* knockout or hepatocyte-specific *NOTCH1* overexpression. At the age of 7 days, NOTCH1^f/f^, NOTCH1^CKO^, and Rosa^*NOTCH1*^ mice were injected with a single dose of DEN. Starting at 4 weeks of age, mice were treated with CCl_4_ for an additional 16 weeks. (**H-J**) Records of body weight, food intake, water intake, liver coefficience, liver-to-spleen ratio, serum AST, ALT, AKP, GGT, pro-inflammatory cytokines TNF-α, IL-6, IL-18, CCL2, and IL-1β levels in DEN-CCl_4_/NOTCH1^f/f^, DEN-CCl_4_/NOTCH1^CKO^, DEN-CCl_4_/NTG and DEN-CCl_4_/HTG groups (*n* = 10 mice per group). (**K**) Representative liver histological analysis detected by H&E staining showing the hepatic pathological injury in the indicated groups (magnification, 100×, *n* = 5 mice per group). (**L**) Representative immunofluorescence images of α-SMA and F4/80 co-expression in liver samples of indicated groups (magnification, 100×, *n* = 10 samples). (**M**) Measurement of tumor nodules on liver and corresponding liver tumor size (*n* = 10 samples). (**N**) Representative immunofluorescence images of KI67 and NOTCH1 co-expression in liver samples of indicated groups (magnification, 100×, *n* = 10 samples). Data are presented as mean ± SEM. The associated experiments were performed independently at least three times. *P* < 0.05 indicates statistical significance

However, the mRNA expression levels of NOTCH1 and KEAP1 in adjacent tissue and 12 individual paired HCC patient tumor samples were assessed in accordance with the potential involvement of activated NOTCH1-KEAP1 pathway in the development of HCC pathogenesis, which has also been confirmed in our current work (Fig. 9A). Additionally, six distinct matched tumor specimens and normal samples from HCC patients were analyzed for comparable NOTCH1 and KEAP1 expression changes (Fig. 9B-D). In fact, HCC samples showed significantly higher NOTCH1 expression but significantly lower NRF2 expression as compared to the neighboring or normal controls (Fig. 9E). Next, we looked at the expression of NOTCH1, KEAP2, and NRF2 in a mouse model of HCC induced by diethylnitrosamine (DEN) + CCl_4_. It should come as no surprise that all HCC tumors produced from HCC pathological phenotypes showed considerably higher NOTCH1 and KEAP1 expression and lower NRF2 abundance (Fig. 9F). Furthermore, the function of NOTCH1-KEAP1 on the pathogenesis of HCC was further investigated in NOTCH1^CKO^ and Rosa^*NOTCH1*^(HTG) mice preconditioned with DEN and an additional 16 weeks of CCl_4_ challenge, with the Flox and Rosa^*NOTCH1*^(NTG) mice serving as controls, respectively, due to the in vitro data in liver cancer cell lines (Fig. 9G). Following DEN + CCl_4_ injection, we observed a substantial drop in body weight and liver-to-spleen ratio in HTG mice compared to the NTG group, but no statistically significant change in food, water, or liver coefficience (Fig. 9H). Interestingly, compared to the corresponding control groups, DEN-CCl_4_-treated HTG mice displayed lower levels of liver enzymes and an inflammatory response, while NOTCH1^CKO^ mice displayed higher levels of AST, ALT, AKP, and GGT as well as pro-inflammatory cytokines (e.g., IL-1β, IL-6, CCL2, IL-18, TNF-α), hepatocellular injury, inflammatory infiltration, and fibrosis (Fig. 9I-L). Additionally, following the DEN-CCl_4_ exposure, Rosa^*NOTCH1*^(HTG) mice showed more tumors, bigger tumors, and positive KI67 expression on the liver surface than NTG mice (Fig. 9M, N). Furthermore, as in the chronic liver inflammation model, we must ascertain whether KEAP1 is necessary for the enhancement of NOTCH1 in the development of HCC. The *NOTCH1-KEAP1* DKO mice were transfected with AdshRNA, Ad*KEAP1*, Ad*NOTCH1*, or Ad*NOTCH1* + Ad*KEAP1* to restore the relevant protein expression, as shown in Supplementary Fig. 10A. The HCC model was then constructed using DEN + CCl_4_ challenge. According to in vitro studies, body weight loss and the liver-to-spleen ratio were more significant following Ad*NOTCH1* + Ad*KEAP1* co-restoration than following AdN*OTCH1* restoration alone (Supplementary Fig. 10B, C). However, when compared to the Ad*NOTCH1* restoration alone group, the Ad*NOTCH1* + Ad*KEAP1* co-restoration mice showed significantly higher levels of pro-inflammatory cytokines (IL-1β, IL-6, IL-18, CCL2, CX3CL1, and TNF-α), liver enzymes (GGT, ALT, and AKP), F4/80-associated inflammatory infiltration, collagen production, tumor nodules, and tumor size in the liver (Supplementary Fig. 10D-I). In order to precisely ascertain the role of the NOTCH1-KEAP1 axis in HCC cell lines, we also carried out extra studies. A noteworthy increase in cell viability and EdU staining intensity was seen in NOTCH1 and KEAP1 doubly deficient-SNU-398, Hep3B, SNU-182, and MHCC97H cells transfected with Ad*NOTCH1* + Ad*KEAP1*. On the other hand, albeit not as much as cells with Ad*NOTCH1* + Ad*KEAP1* co-restoration, cells transfected with Ad*NOTCH1* or Ad*KEAP1* alone, respectively, also showed a rise in cell viability and EdU intensity. These results imply that NOTCH1 stimulates cell division in vitro, contingent on KEAP1 activity (Supplementary Fig. 11A, B). Furthermore, our work lends credence to the idea that the NOTCH1-KEAP1 pathway contributes to the etiology of HCC. The transwell investigation, which revealed a marked increase in tumor cell invasion and migration when Ad*NOTCH1* + Ad*KEAP1* was co-transfected as opposed to Ad*KEAP1* or Ad*NOTCH1* transfection, supports our findings (Supplementary Fig. 11C). As anticipated, transfected cells exhibited markedly elevated levels of epithelial-mesenchymal transition (EMT)-related markers, including vimentin, N-cadherin, fibronectin, TGF-β1, and MMP13. Also, Ad*NOTCH1* + Ad*KEAP1* co-restoration significantly reduced the expression of E-cadherin (Supplementary Fig. 11D). All of these findings point to NOTCH1 recruiting and interacting with KEAP1 both in vivo and in vitro to promote the progression of HCC.

## Discussion

Oxidative stress, mitochondrial dysfunction, inflammatory response, collagen deposition, and cell death are the primary mediators in the pathogenesis of inflammatory liver injury and HCC, according to mounting evidence. These factors are all closely related to hepatocellular injury [3, 23]. NOTCH signaling is a developmental route that is involved in numerous cellular processes and encourages damage to the liver, kidney, heart, and skin. Additionally, research has shown that hepatocytes from a variety of stress-induced liver conditions, including hepatitis and HCC, have higher NOTCH1 activity. Furthermore, by blocking the inflammatory response mediated by the TLR4 pathway and osteopontin, defective NOTCH signaling can decrease the evolution of fatty liver [20, 22]. However, there are few studies that link ferroptosis in liver damage to hepatocyte NOTCH1, and the underlying mechanisms are still mostly unknown. Targeting NOTCH1 may therefore be a promising therapeutic strategy for the management of liver injury.

**Fig. 10:**
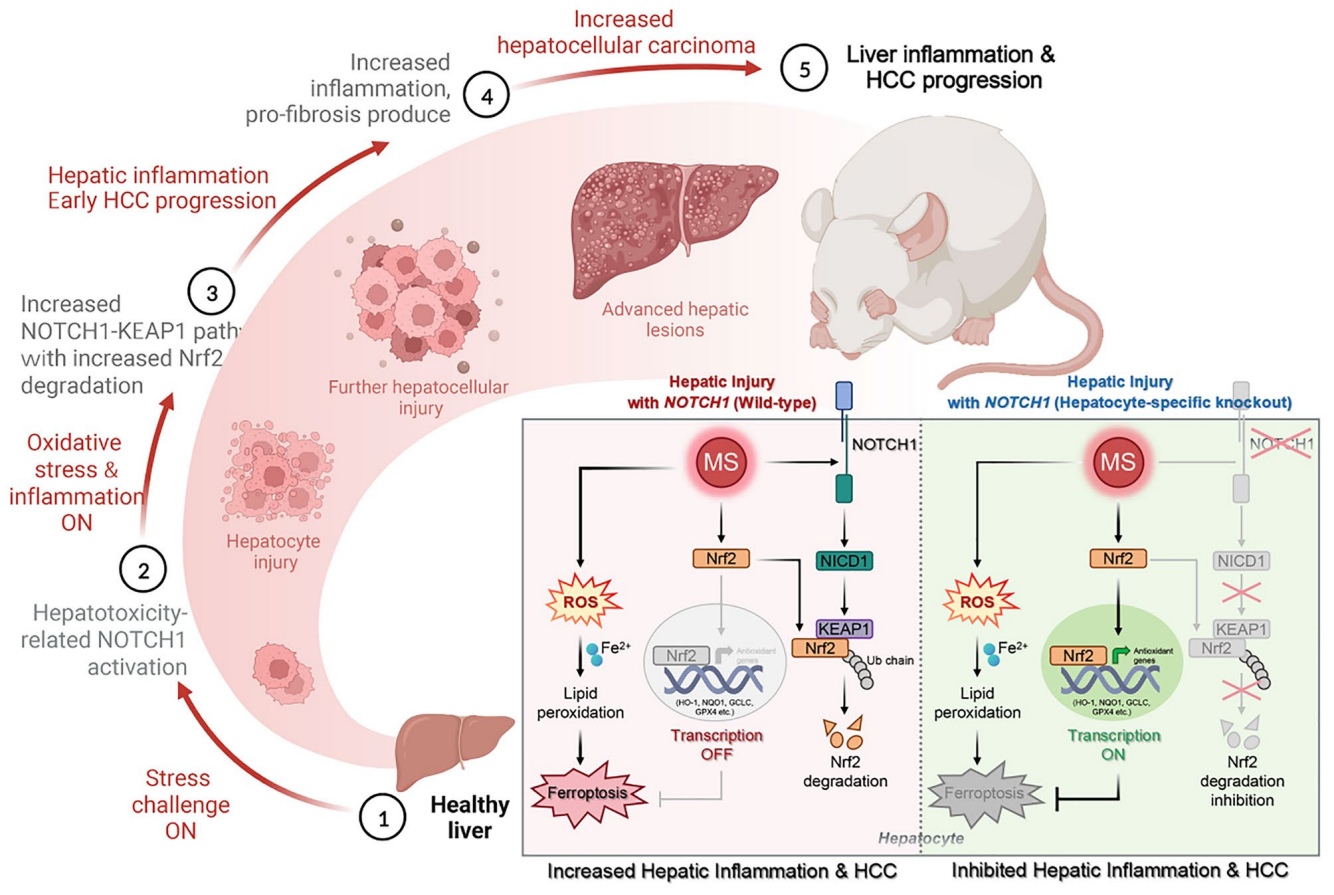
Schematic diagram of NOTCH1 regulating KEAP1/NRF2 signaling in chronic liver injury and HCC progression. Stress challenges (such as TAA, CCl4, DMN, and DEN) cause NOTCH1(NICD1) signaling to be abnormally overexpressed. By directly interacting with and recruiting KEAP1, NICD1 promotes hepatocyte KEAP1 expression levels. Elevated KEAP1 causes NRF2 ubiquitination and facilitates NRF2 degradation, which lowers the transcription of protective genes such GCLC, HO-1, NQO1, and GPX4. Hepatocyte ferroptosis and excessive ROS generation are caused by this biological process, which ultimately results in the pathological development of hepatocellular damage. Notably, gene therapy that targets NOTCH1, specifically its NICD1 deletion, can disrupt its ability to bind and recruit KEAP1, improving the stability and expression of the NRF2 protein and its downstream protective signaling pathway, thereby suppressing chronic liver injury and the progression of HCC that is linked to it

Here, we treated chronic liver inflammation and HCC in mice by selectively disrupting the NOTCH1-KEAP1 link using genetic therapy. Our research revealed a strong relationship between NOTCH1 (NICD1) in hepatocytes, a crucial regulator of liver injury, and the extent of chronic inflammatory injury in hepatitis and HCC. Hepatocyte-specific *NOTCH1* deletion decreased hepatocellular damage and hepatic inflammatory pathology in rats induced with CCl_4_ or DMN, but its overexpression markedly exacerbated symptoms. Mechanically, when CCl_4_ is administered to hepatocytes, the ANK domain of NICD1 directly interacts with and recruits KEAP1, which in turn causes NRF2 to become ubiquitinated and lose stability. The subsequent breakdown of NRF2 resulted in ferroptosis, oxidative stress, and mitochondrial impairment, all of which contributed to hepatocellular damage. Chronic liver inflammation then developed, and HCC progressed (Fig. 10). Thus, our study expanded our understanding of the mechanism underlying the pathophysiology of liver injury by presenting fresh evidence linking NOTCH1 to the control of KEAP1 in hepatocyte injury. As a result, the NOTCH1-KEAP1 axis might be a novel target for liver disease treatment.

Ferroptosis, a type of cell death marked by iron-dependent lipid peroxidation, has also been extensively studied in addition to NOTCH1’s functional significance in liver injury [24, 25]. It differs from necrosis and apoptosis in that it is mostly caused by iron buildup and lipid peroxidation inside cells. Preventing excessive oxidative stress and preserving cellular integrity depend on this process. Oxidative stress and iron metabolism dysregulation have been connected to ferroptosis’s function in liver damage [26, 27]. Elevated glucose levels can cause increased ROS production and iron accumulation in fatty liver development, both of which aid in the induction of ferroptosis [26, 28]. However, in a mouse model created by a high-fat diet, ferroptosis inhibition using a GPX4 activator may offer protection against metabolic-associated fatty liver disease [29]. Notably, ferroptotic cell death is also connected to fibrosis and the inflammatory response. As a result, ferroptosis influences several signaling pathways linked to inflammation, such as NF-κB, NLRP3, and MAPKs, which results in the generation of cytokines and chemokines that promote inflammation [30, 31]. In turn, this inflammatory environment worsens liver damage and accelerates the development of hepatitis. Furthermore, ferroptosis causes TGF-β signaling to become activated, which results in an excess of extracellular matrix components and ultimately aids in the development of hepatic fibrosis [32]. Targeting ferroptotic cell death may therefore reduce hepatocyte fibrosis and inflammation, reducing or delaying the development of liver damage [33]. Consistent with earlier findings, we verified the abnormal expression of NOTCH1(NICD1) in hepatocytes treated with CCL_4_ and DMN in our current investigation. Notably, by lowering inflammation and fibrosis and decreasing ROS production in the liver, hepatocyte-specific deletion of *NOTCH1* had a hepatoprotective impact against chronic hepatic inflammation and the formation of HCC. Conversely, other research indicates that boosting NOTCH1 activation upregulates MnSOD and reduces ROS, hence preventing cellular damage, whereas decreasing NOTCH1 signaling increases intracellular ROS and decreases GSH, speeding up oxidative stress [34, 35]. We hypothesized that the various cell types and pathogenic stimuli could be linked to this discrepancy. Through transcriptomic and molecular investigations, we demonstrated a previously under-recognized function of NOTCH1 in inducing hepatocyte ferroptosis. As evidenced by elevated GSH/GSSG ratio, elevated GPX4 and SLC7A11 expression, decreased iron concentrations, and decreased ACSL4 expression levels-all of which are indicators of ferroptosis-hepatocyte-specific *NOTCH1* deletion markedly reduced ferroptotic cell death in the hepatocytes of CCL_4_-traeted animals. It has been consistently observed that NOTCH1 knockdown reduces ferroptosis in human hepatocytes following CCL_4_ stress with enhanced mitochondrial functioning. Recent research has also connected ferroptosis under various stressors to NOTCH1 signaling activity [36, 37]. Significant NOTCH1 downregulation promotes ferroptosis in lens epithelial cells (LECs) by impairing the NRF2/GPX4 antioxidant pathway, subsequently contributing to age-related cataracts (ARCs) development [38]. NOTCH1 is also critical for protecting against ferroptosis in mouse embryo becomes fibroblasts, which participates in neuronal dysfunction and death by ferroptosis [39]. By inhibiting GPX4-mediated ferroptosis in rat renal proximal tubular epithelial cells, NOTCH1 activation can primarily counteract the emodin-induced nephrotoxicity [40]. The function of NOTCH1 in hepatocytes under CCL_4_ stimuli during the progression of liver injury is an undesirable factor in our current data, in contrast to its regulatory role on ferroptosis in pathological LECs and renal proximal tubular epithelial cells. This could be because different cell types and tissues have different biological behaviors and distinct pathogenic stresses. Therefore, NOTCH1 is a promising molecular target for treatments of chronic liver injury and offers a way to modify ferroptosis.

Of note, KEAP1 and NRF2 are key regulators of the cellular antioxidant response, playing a crucial role in protecting cells from oxidative stress. In fatty liver development, the KEAP1-NRF2 signaling dysregulation has been implicated in the pathogenesis of the disease [41, 42]. Under normal physiological conditions, NRF2 is primarily sequestered in the cytoplasm by KEAP1, preventing its nuclear translocation and subsequent activation of antioxidant genes. KEAP1 functions as an adapter of Cullin3-Rbx1 E3 ligase, resulting in NRF2 ubiquitination and proteasomal degradation [43]. However, under oxidative stress, the cysteine residues of KEAP1 are covalent modified, resulting in impaired ubiquitination and KEAP1-regulated NRF2 degradation. Subsequently, NRF2 translocates into the nucleus and binds to small Maf proteins, activating the transcription of antioxidant and phase II detoxification enzymes, thereby reducing oxidative injury and promoting cellular defense mechanisms. Several studies have demonstrated that the NRF2/KEAP1 pathway is impaired, resulting in reduced antioxidant defenses and increased oxidative stress [44, 45]. High glucose levels can cause KEAP1 overexpression, which in turn induces NRF2 degradation, thereby inhibiting NRF2 activity and reducing the expression of antioxidant genes. This increase in oxidative stress contributes to the development and progression of hepatocyte damage in hepatic fibrosis or steatohepatitis. Additionally, growing studies have identified the role of KEAP1-NRF2 in regulating ferroptotic cell death [46, 47]. Ferroptosis is triggered by the dysregulation of various cellular processes, including lipid metabolism, iron homeostasis, and the antioxidant defense system [48]. In the context of steatohepatitis, the dysregulation of the KEAP1-NRF2 pathway can lead to an imbalance between pro-oxidant and antioxidant pathways, resulting in the accumulation of lipid peroxides and the induction of ferroptosis in hepatocyte and hepatic stellate cells [24, 28]. It has been reported that the KEAP1-NRF2 signaling pathway and NOTCH1 signaling can be regulated by reciprocal transcriptional mechanisms. The crosstalk between NRF2 and NOTCH1 influences the expression of defense systems against both endogenous and exogenous stressors, thereby contributing to cellular protection and enhancing the maintenance of cellular homeostasis. Recently, KEAP1-NRF2 signaling is reported to be activated by NOTCH1, thereby providing protection to the heart against ischemia/reperfusion (I/R) injury through the inhibition of mitochondrial ROS generation and enhances mitochondrial bioenergetics [49, 50]. Interestingly, it has been reported that the NFE2L2 gene, which encodes the NRF2 protein, is a downstream target modulated by NOTCH signaling [51]. Herein, NRF2 and NOTCH signaling may mutually regulate each other. In our study, we newly found that NOTCH1 activation had a positive correlation with KEAP1 during liver injury progression. Hepatocyte-specific deletion of NOTCH1 significantly restrained KEAP1 expression levels, accompanied with higher NRF2 protein expression in hepatocyte of CCl_4_-treated mice, contributing to the suppression of ROS production and ferroptosis, which exerted hepatoprotective effects against chronic liver inflammation and HCC progression. Also, NOTCH1 also strongly relies on KEAP1 to promote oxidative stress and NRF2 degradation, and hepatocyte-specific KEAP1 loss greatly impeds the NOTCH1-mediated hepatic injury process.

Of note, the ANK domain plays a critical role in the recruitment of activators and transactivation for NOTCH1 signaling. This particular ANK domain typically functions as a site for protein-protein interactions, which is essential for the association of NICD1 with downstream molecules [52]. In acute T-lymphoblastic leukemia (T-ALL), the ANK region of NOTCH1 participates in the interaction with other signaling molecules during this process, thereby affecting cell proliferation and differentiation [53]. Our study similarly showed that NOTCH1 could directly interact with KEAP1 for its recruitment in hepatocyte under CCl_4_ challenge, leading to NRF2 ubiquitination and degradation, which contributed to oxidative injury and ferroptosis. Intriguingly, the mutation of NOTCH1(ΔANK) fail to facilitate KEAP1 expression levels in vitro, suggesting that ANK domains determined the KEAP1 interaction and its downstream NRF2 signaling activity in the progression of liver injury. In contrast, Ad*NOTCH1*-induced KEAP1 downregulation and NRF2 upregulation were considerably recovered upon the overexpression of ANK domain restoration, leading to accumulation of ROS and ferroptosis. Thus, these findings further indicated that the core function of ANK domain of NOTCH1 may be a strict target for intercepting metabolic insults-driven ROS generation, ferroptotic cell death and associated metabolic dysregulation involved in liver injury or HCC onsetting via regulating KEAP1-NRF2 signaling pathway (Fig. 10).

## Conclusions

In this study, we first identified the hepatocyte NOTCH1 as a accelerated liver injury checkpoint that directly interacts with KEAP1 and specifically recruits KEAP1 in a KEAP1-dependent formation to inhibit NRF2 activation, leading to oxidative damage and ferroptotic cell death, promoting liver injury development and HCC progression.

## Supplementary Information

Below is the link to the electronic supplementary material.

Supplementary Material 1

Supplementary Material 2

## Data Availability

No datasets were generated or analysed during the current study.

## Abbreviations

HCC: Hepatocellular carcinoma
AAV: Adeno-associated virus
KEAP1: Kelch-like ECH-associated protein 1
NICD1: NOTCH1 intracellular domain
NRF2: NF-E2-related factor
EMT: Epithelial-mesenchymal transition
AGEs: Advanced glycation end-products, AGEs
AKP: Alkline phosphatase
AST: Aspartate aminotransferase
ALT: Alanine transaminase
GST: Glutathione S-transferase
WT: Wild-type
TG: Triglyceride
HBV: Hepatitis B virus
HCV: Hepatitis C virus
NASH: Non-alcohol steatohepatitis
