# Supplementary material for: Targeting NOTCH1-KEAP1 axis retards chronic liver injury and liver cancer progression via regulating stabilization of NRF2
Source: J Exp Clin Cancer Res. 2025 Aug 9;44:232. doi: 10.1186/s13046-025-03488-3 (PMC12335071; doi:10.1186/s13046-025-03488-3)
Supplement: Supplementary file 1 — Supplementary Material 1 [file 13046_2025_3488_MOESM1_ESM.docx]

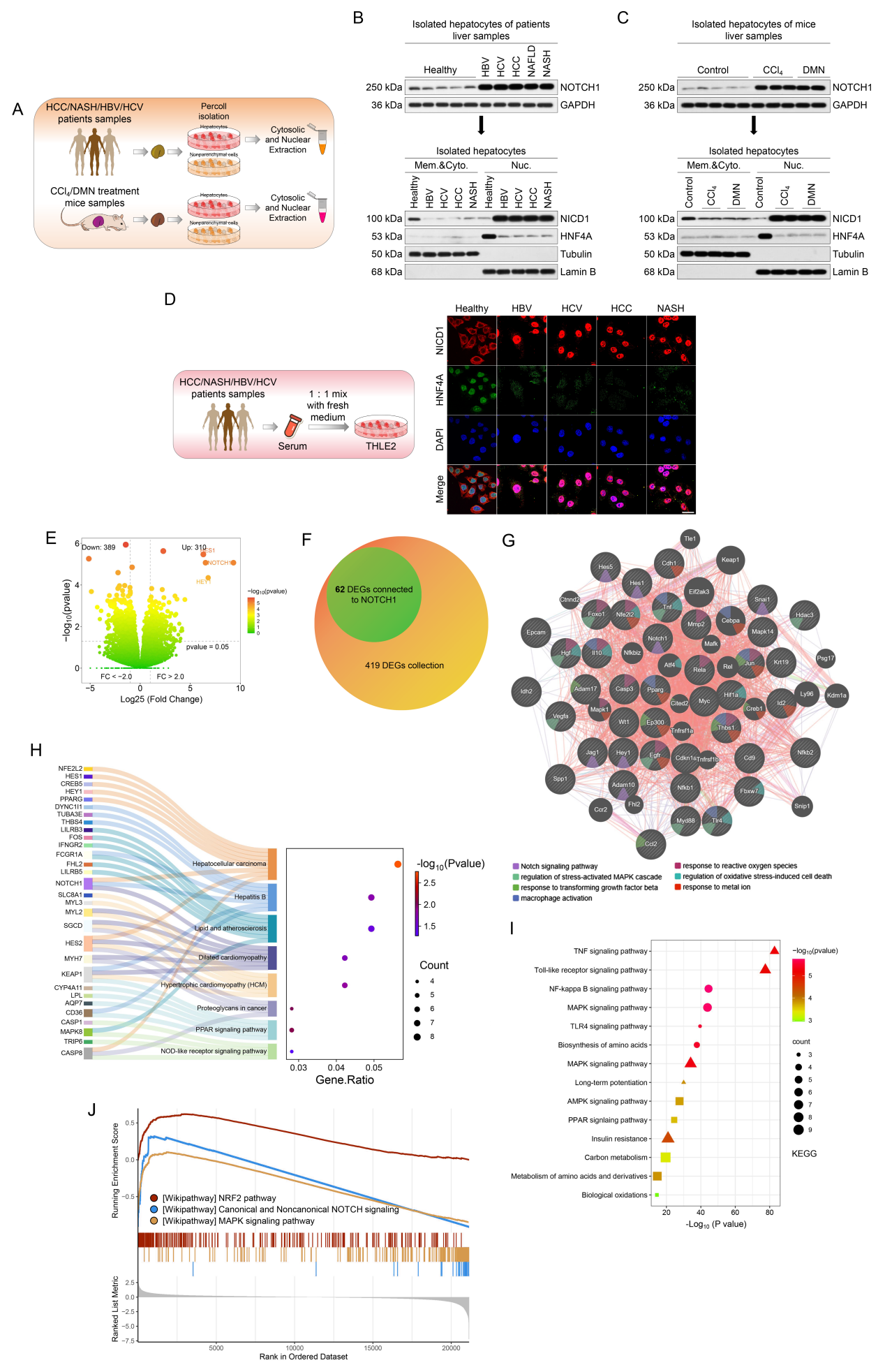


**Supplementary figure 1. High NOTCH1 expression is involved in chronic liver inflammation progression.** (**A**) Experimental design showing the protocol of hepatocytes isolation, and cytosolic and nuclear extraction. (**B, C**) Western blotting analysis showing the NOTCH1 expression in isolated hepatocytes of patients (B) and mouse models (B) (*n*=5 samples). (**D**) Experimental design showing the protocol of medium containing patients serum mixture-cultured THLE2 in vitro. Then, immunofluorescence analysis showing the NICD1 and HNF4A expression in cultured THLE2 cells (magnification, 200×, *n*=5 samples). (**E**) Volcano plots showing genes that are differentially expressed in the isolated liver samples from CCl_4_-treated mice versus the control group by RNA-Seq analysis (|LogFC|>1.5 & p.adj<0.05; *n*=10 per group). (**F**) A total of 419 DEGs was identified and 62 of these genes were associated with the NOTCH1 signal. (**G**) Circle diagram analyzed by GeneMANIA further showing NOTCH1 protein interaction network with potential connected signals in Mus musculus. (**H**) Sankey diagram enrichment and (**I**) enriched KEGG terms related to inflammation, Notch1 signaling, TGF-beta signaling, and ferroptosis based on the RNA-Seq of liver tissue of CCl_4_-induced mice. (**J**) GSEA showing the proinflammatory and profibrotic mediators, Notch signaling and Nrf2 pathway enrichment score in liver tissue of CCl_4_-induced mice (*n*=10 per parameter).

**
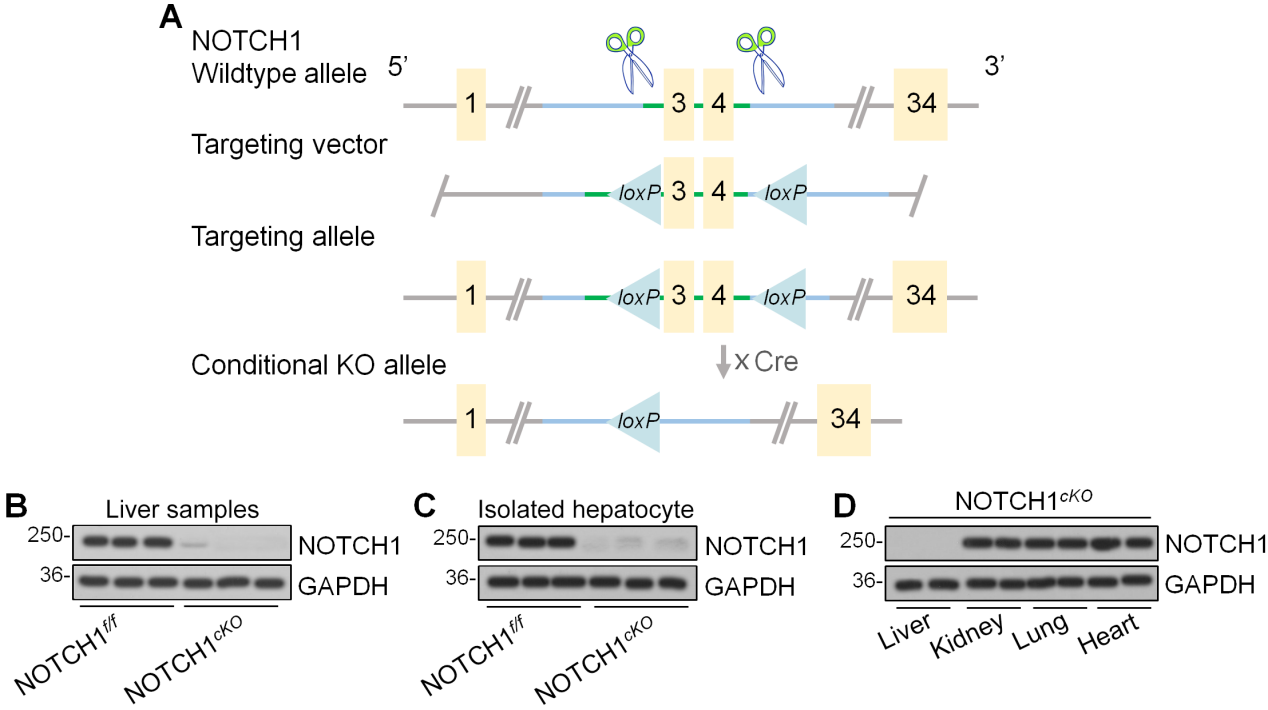
**

**Supplementary figure 2. Hepatocyte-specific** ***NOTCH1* deficiency mice construction.** (**A**) Schematic workflow showing the establishment of the hepatocyte-specific *NOTCH1* knockout (NOTCH1^CKO^) mouse strain. (**B**) Western blot showing NOTCH1 expression in the isolated liver samples from NOTCH1^f/f^ and NOTCH1^CKO^ mice (*n*=4 per group). (**C**) Western blot showing NOTCH1 expression in the isolated primary hepatocytes of mice from the indicated groups (*n*=4 per group). (**D**) Western blot showing NOTCH1 expression in liver, kidney, lung and heart of indicated mice (*n*=4 per group).


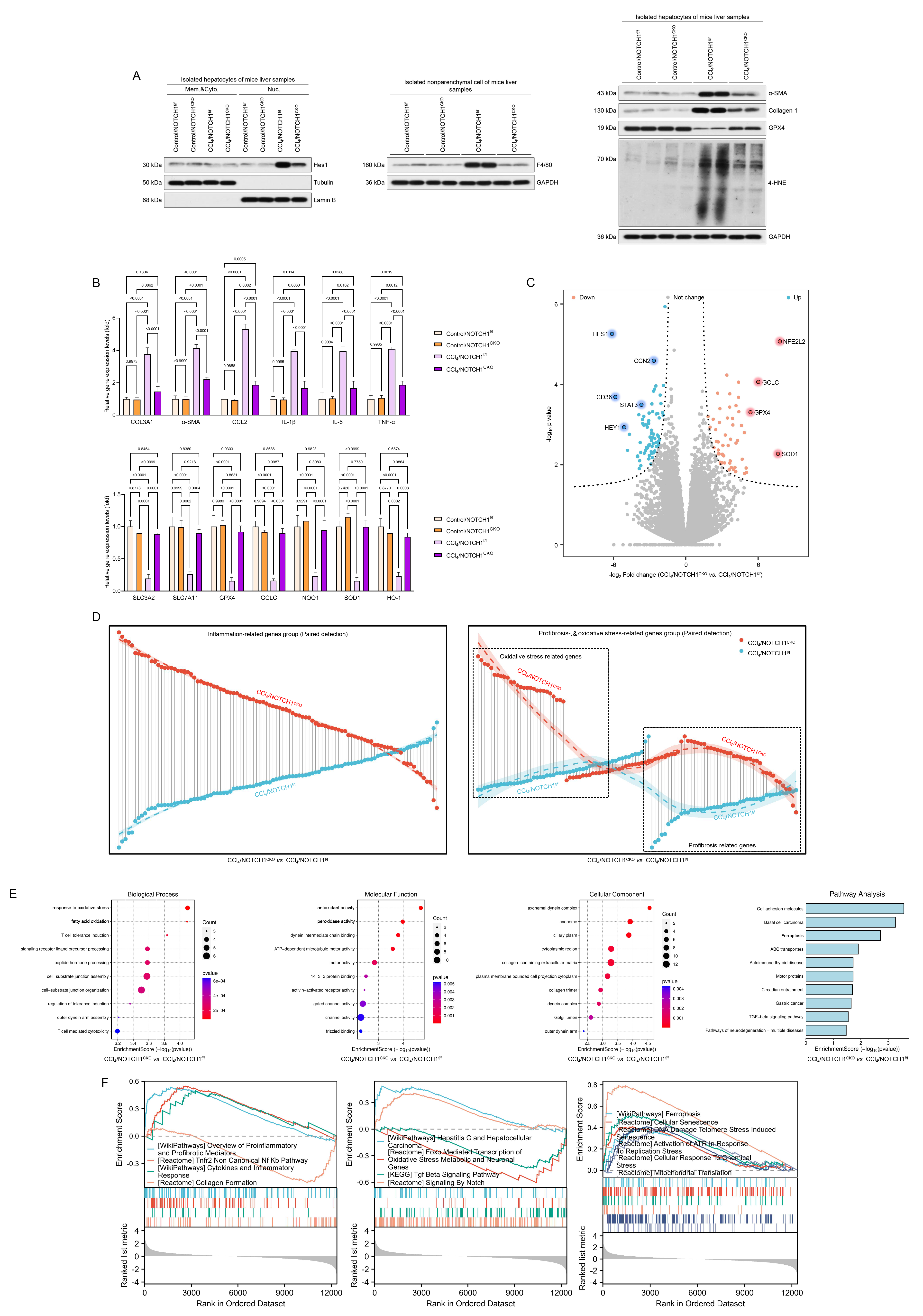


**Supplementary figure 3. Hepatocyte-specific *NOTCH1* deficiency mitigates inflammation, oxidative stress, mitochondrial dysfunction and fibrosis in CCl_4_-induced mice.** (**A**) Western blotting analysis showing the Hes1 protein expression in isolated hepatocytes nucleus (left), F4/80 protein expression in non-parenchymal cells (middle), and α-SMA, Collagen-1, GPX4 and 4-HNE levels (right) in the indicated groups (*n*=5 per group). (**B**) The mRNA fold changes of genes related to inflammatory response and fibrosis in the isolated liver samples of mice from the shown groups of mice by qPCR (*n*=5 per group). qPCR results for the expression of genes related to ferroptosis and oxidative stress in the isolated liver samples of the indicated groups of mice (*n*=5 per group). (**C**) Volcano plots showing DEGs in the isolated liver samples from CCl_4_/NOTCH1^CKO^ mice versus the CCl_4_/NOTCH1^f/f^ group by RNA-Seq analysis (|LogFC|>1.5 & p.adj<0.05; *n*=5 per group). (**D**) Paired detection for fibrosis-, oxidative stress-, and inflammation-associated gene expression profiles following the RNA-Seq analysis. (**E**) GO enrichment analysis showing the enriched signaling pathways based on RNA-seq analysis; and KEGG terms related to ferroptosis based on the RNA-Seq dataset. (**F**) GSEA pathway enrichment analysis of pathways related to NOTCH1 intracellular domain regulates transcription, proinflammatory and profibrotic mediators, oxidative stress response and ferroptosis related to RNA-seq analysis. Data are presented as mean ± SEM. The associated experiments were performed independently at least three times. *P* <0.05 indicates statistical significance.


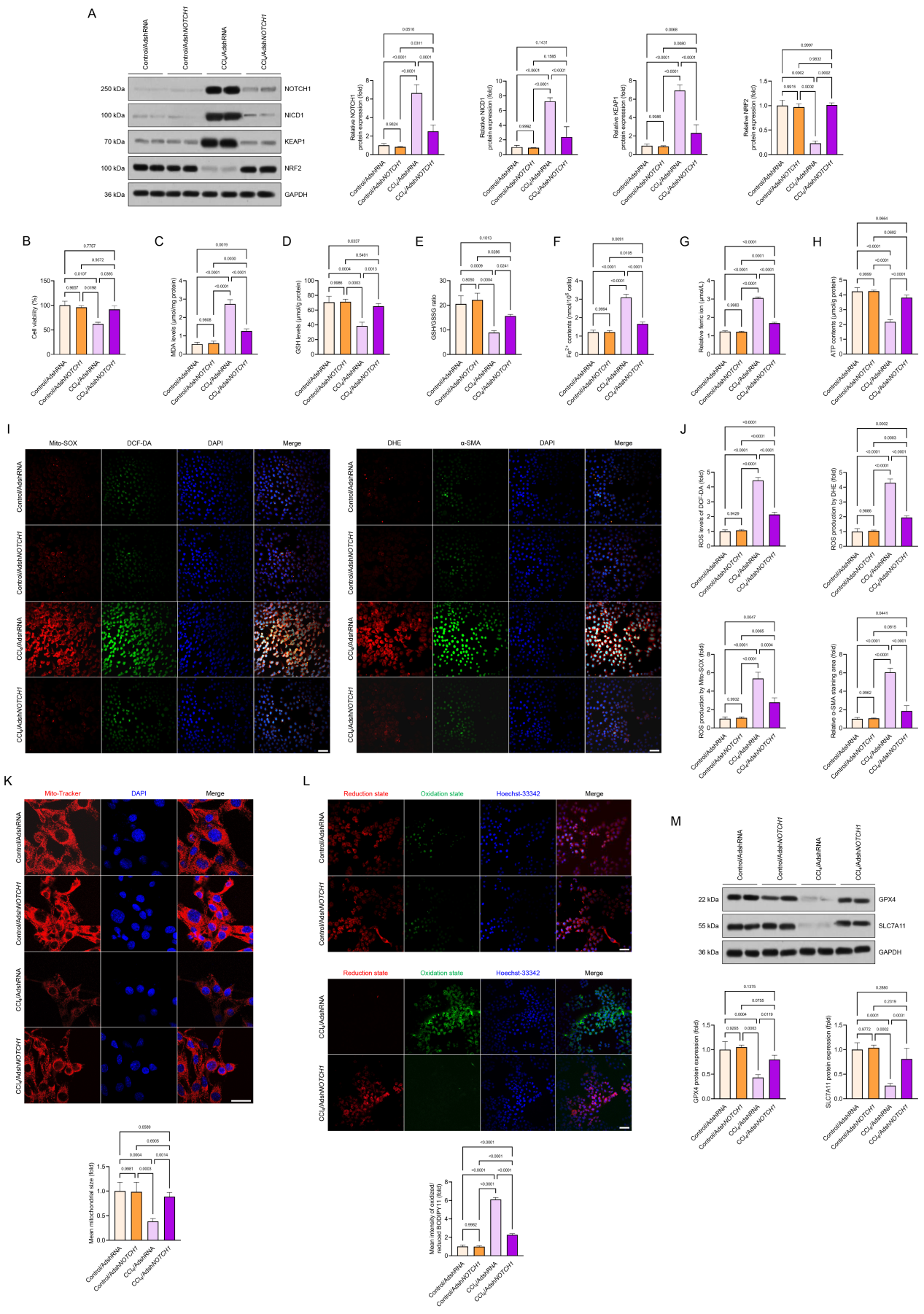


**Supplementary figure 4. Suppression of NOTCH1 mitigates oxidative stress and ferroptosis in human THLE2 cells in response to CCl_4_ challenge.** (**A**) Western blotting analysis for NOTCH1, NICD1, KEAP1 and NRF2 protein expression levels in human THLE2 cells with adenovirus transfection-mediated suppression of NOTCH1 (Adsh*NOTCH1*) in response to CCl_4_ challenge for 10 h. AdshRNA served as a control (*n*=5 per group). (**B**) Cell viability by CCK8 analysis in human THLE2 cells with Adsh*NOTCH1* transfection in the absence or presence of CCl_4_ challenge for 10 h. AdshRNA was used as a control (*n*=10 per group). (**C**) MDA levels, (**D**) GSH contents, (**E**) GSH/GSSG ratio, (**F**) Fe^2+^ levels, (**G**) relative ferric ion levels, and (**H**) ATP levels were examined in THLE2 cells from the indicated groups (*n*=10 per group). (**I**) DCF-DA, Mito-SOX, DHE and α-SMA staining were performed to examine ROS production and α-SMA expression in CCl_4_-treated THLE2 cells from the shown groups (*n*=6 per group). (**J**) Quantification for ROS generation was performed based on DCF-DA, DHE, Mito-SOX staining, and α-SMA expression respectively (*n*=10 per group). (**K**) Mito-Tracker staining was conducted to detect mitochondrial structures in THLE2 cells from the indicated groups. Mitochondrial size was quantified related to Mito-Tracker staining (*n*=6 per group). (**L**) BODIPY-C11 staining was performed to assess lipid peroxidation in NOTCH1-knockdown THLE2 cells after CCl_4_ treatment for 10 h. The ratio of oxidized fluorescence to reduced fluorescence was quantified based on BODIPY-C11 staining (*n*=10 per group). (**M**) Western blot showing the levels of GPX4 and SLC7A11 in THLE2 cells infected with NOTCH1 knockdown adenovirus (Adsh*NOTCH1*) in the absence or presence of CCl_4_ treatment. AdshRNA was used as a control (*n*=5 per group). Data are presented as mean ± SEM. The associated experiments were performed independently at least three times. *P* <0.05 indicates statistical significance.


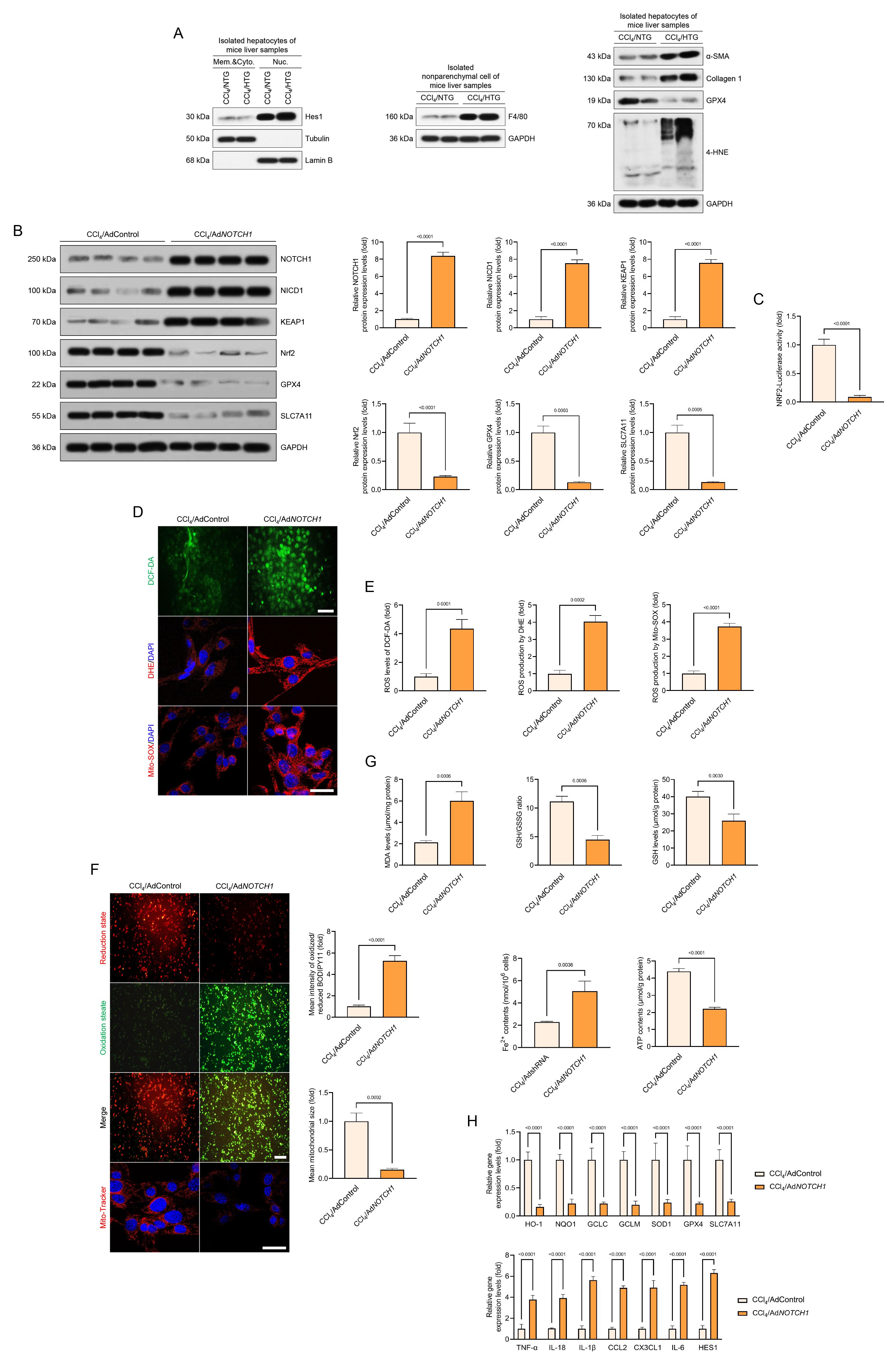


**Supplementary figure 5. NOTCH1 overexpression promotes oxidative stress and ferroptosis in human THLE2 cells in response to CCl_4_ challenge.** (**A**) Western blotting analysis showing the Hes1 protein expression in isolated hepatocytes nucleus (left), F4/80 protein expression in non-parenchymal cells (middle), and α-SMA, Collagen-1, GPX4 and 4-HNE levels (right) in the indicated groups (*n*=5 per group). (**B**) Western blotting analysis for NOTCH1, NICD1, KEAP1, NRF2, GPX4, and SLC7A11 protein expression levels in human THLE2 cells (*n*=6 per group). (**C**) NRF2 activity detected by luciferase assay after Ad*NOTCH1* transfection in response to CCl_4_ challenge (*n*=6 per group). (**D, E**) Quantification for ROS generation was performed based on DCF-DA, and DHE staining (*n*=10 per group). (**F**) BODIPY-C11 staining was performed to assess lipid peroxidation in Ad*NOTCH1-*transfected THLE2 cells after CCl_4_ treatment for 10 h. The ratio of oxidized fluorescence to reduced fluorescence was quantified based on BODIPY-C11 staining (*n*=10 per group). (G) MDA levels, GSH contents, GSH/GSSG ratio, Fe^2+^ levels, and ATP levels were examined in THLE2 cells from the indicated groups (*n*=10 per group). (**H**) mRNA expression levels of anti-oxidative stress-, ferroptosis-, and pro-inflammation-related genes expression in the indicated groups (*n*=10 per group). Data are presented as mean ± SEM. The associated experiments were performed independently at least three times. *P* <0.05 indicates statistical significance.


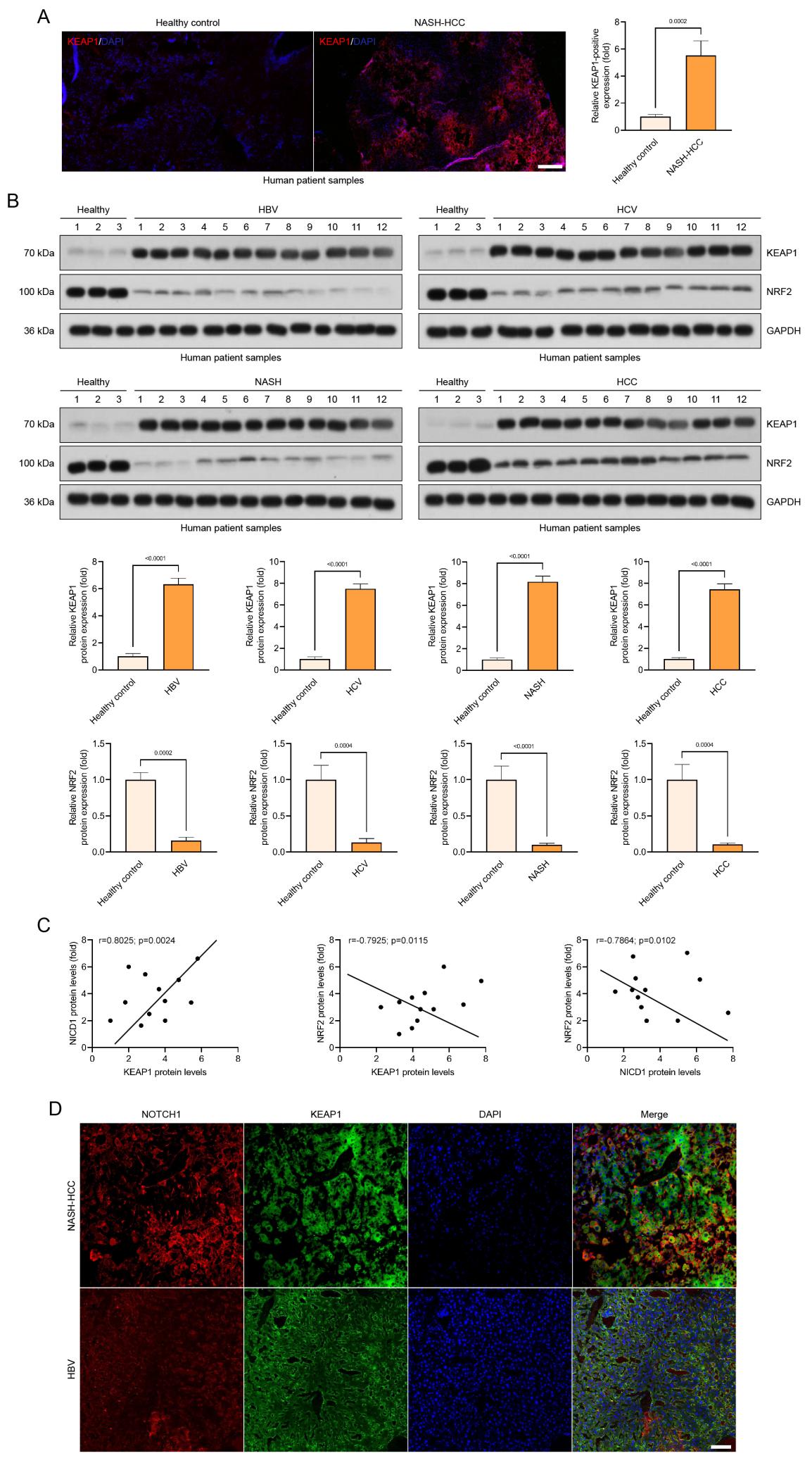


**Supplementary figure 6. A positive correlation between NOTCH1 and KEAP1 protein expression levels in livers injury patients.** (**A**) Representative immunofluorescence images showing positive staining of KEAP1 in liver tissue sections of liver injury patients (*n*=12) or the normal individuals (*n*=6). The mean immunostaining of KEAP1 was quantified following immunofluorescence analysis. (**B**) Western blotting analysis showing the KEAP1 and NRF2 protein expression in the liver samples collected from patients with HBV, HCV, NASH and HCC pathological phenotype (*n*=12 for patients; *n*=6 for controls). (**C**) Pearson’s r correlation analysis between NICD1, KEAP1 and NRF2 protein levels in liver injury patients (*n*=12). (**D**) Representative immunofluorescence images showing positive staining of NOTCH1 and KEAP1 in liver tissue sections of HBV and NASH-HCC patients (*n*=12). Data are presented as mean ± SEM. The associated experiments were performed independently at least three times. *P* <0.05 indicates statistical significance.


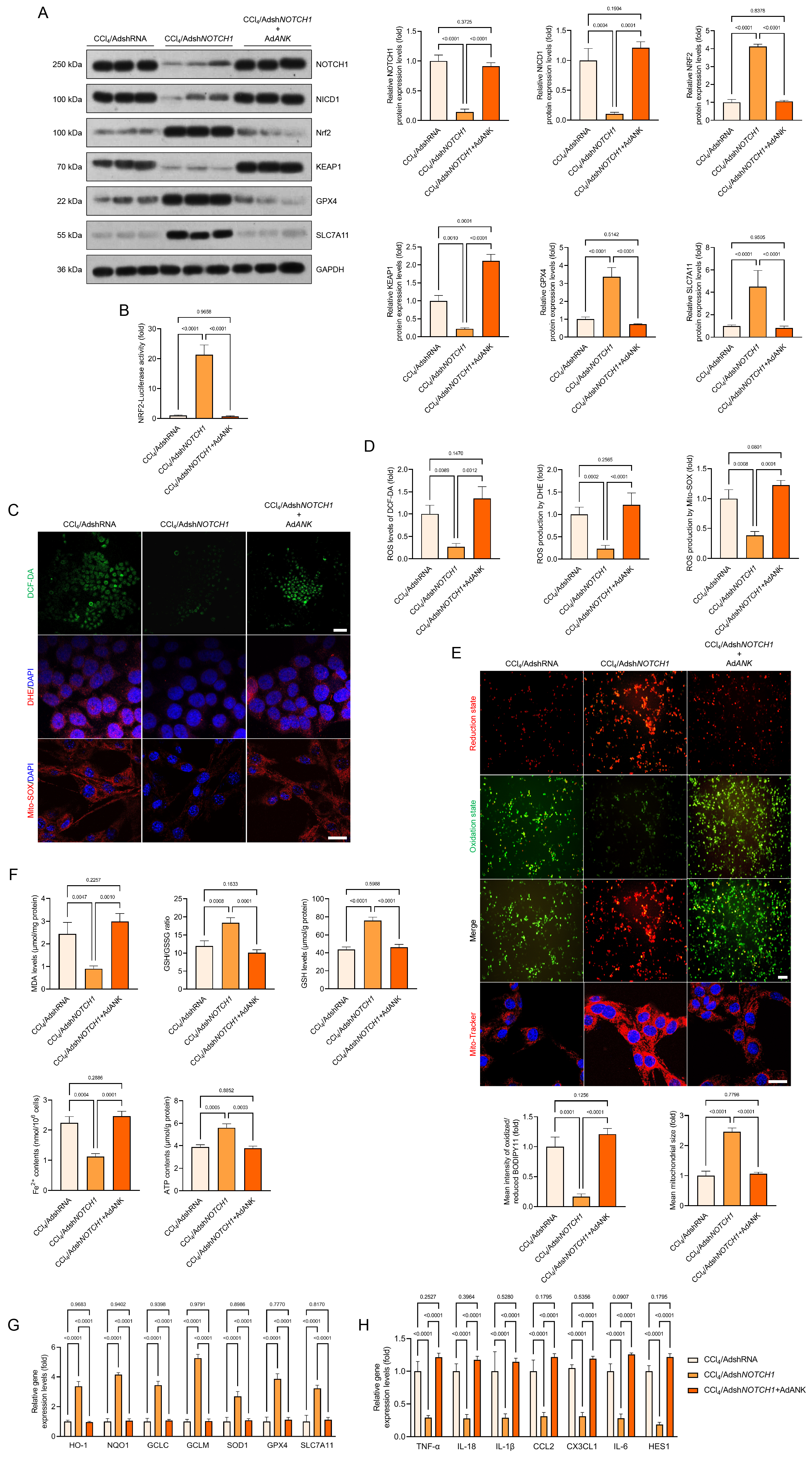


**Supplementary figure 7. ANK domain of NOTCH1 restoration re-facilitates oxidative stress and ferroptosis in human THLE2 cells in response to CCl_4_ challenge.** (**A**) Western blotting analysis showing the NOTCH1, NICD1, NRF2, KEAP1, GPX4 and SLC7A11 protein expression in the Adsh*NOTCH1*-, Adsh*NOTCH1*+AdANK-, and AdshRNA-transfected THLE2 cells under CCl_4_ challenge (*n*=4 per group). Data are presented as mean ± SEM. The associated experiments were performed independently at least three times. *P* <0.05 indicates statistical significance. (**B**) NRF2 activity detected by luciferase assay after Adsh*NOTCH1*-, Adsh*NOTCH1*+AdANK-, and AdshRNA transfection in response to CCl_4_ challenge (*n*=6 per group). (**C, D**) Quantification for ROS generation was performed based on DCF-DA, DHE, and Mito-SOX staining (*n*=10 per group). (**E**) BODIPY-C11 staining was performed to assess lipid peroxidation in the indicated transfected THLE2 cells after CCl_4_ treatment for 10 h. The ratio of oxidized fluorescence to reduced fluorescence was quantified based on BODIPY-C11 staining (*n*=10 per group). (**F**) MDA levels, GSH contents, GSH/GSSG ratio, Fe^2+^ levels, and ATP levels were examined in THLE2 cells from the indicated groups (*n*=10 per group). (**G, H**) mRNA expression levels of anti-oxidative stress-, ferroptosis-, and pro-inflammation-related genes expression in the indicated groups (*n*=10 per group). Data are presented as mean ± SEM. The associated experiments were performed independently at least three times. *P* <0.05 indicates statistical significance.


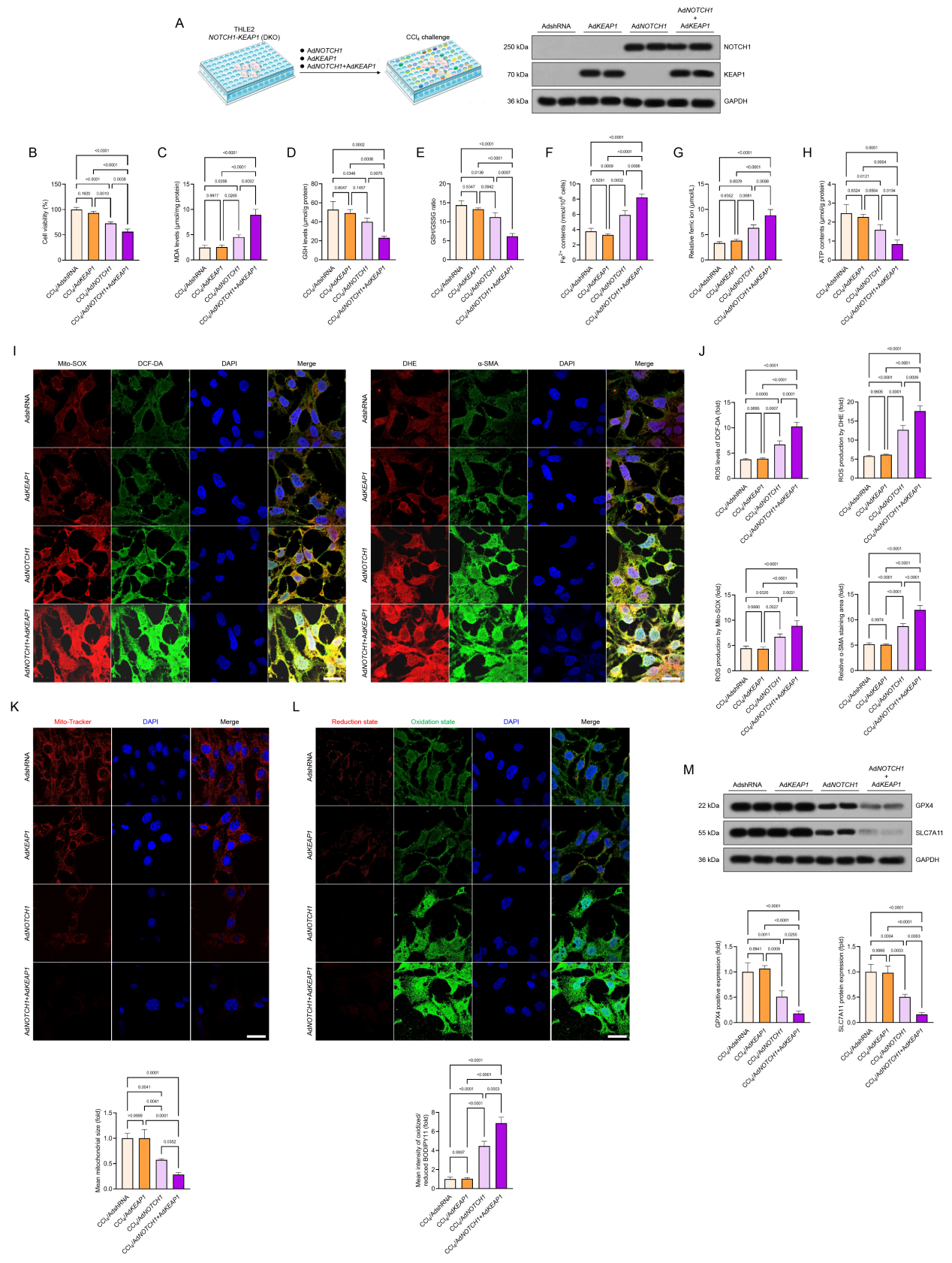


**Supplementary figure 8. KEAP1 is required for NOTCH1 function-mediated development of oxidative stress and ferroptosis in human THLE2 cells in response to CCl_4_ challenge.** (**A**) Schematic diagram for Ad*NOTCH1*-, Ad*KEAP1*-, and Ad*NOTCH1*+Ad*KEAP1*-transfected THLE2 cells with CCl_4_ challenge in *NOTCH1* and *KEAP1* dual-deficient THLE2 cells. The transfection efficiency in transfected THLE2 cells was detected by western blotting assay (*n*=4 per group). (**B**) Cell viability by CCK8 analysis in human transfected THLE2 cells in the absence or presence of CCl_4_ challenge for 10 h. AdshRNA was used as a control (*n*=10 per group). (**C**) MDA levels, (**D**) GSH contents, (**E**) GSH/GSSG ratio, (**F**) Fe^2+^ levels, (**G**) relative ferric ion levels, and (**H**) ATP levels were examined in THLE2 cells from the indicated groups (*n*=10 per group). (**I**) DCF-DA, Mito-SOX, DHE and α-SMA staining were performed to examine ROS production and α-SMA expression in CCl_4_-treated THLE2 cells from the shown groups (*n*=6 per group). (**J**) Quantification for ROS generation was performed based on DCF-DA, DHE, Mito-SOX staining, and α-SMA expression respectively (*n*=10 per group). (**K**) Mito-Tracker staining was conducted to detect mitochondrial structures in THLE2 cells from the indicated groups. Mitochondrial size was quantified related to Mito-Tracker staining (*n*=6 per group). (**L**) BODIPY-C11 staining was performed to assess lipid peroxidation in transfected THLE2 cells after CCl_4_ treatment for 10 h. The ratio of oxidized fluorescence to reduced fluorescence was quantified based on BODIPY-C11 staining (*n*=10 per group). (**M**) Western blot showing the levels of GPX4 and SLC7A11 in transfected THLE2 cells in the absence or presence of CCl_4_ treatment. AdshRNA was used as a control (*n*=5 per group). Data are presented as mean ± SEM. The associated experiments were performed independently at least three times. *P* <0.05 indicates statistical significance.


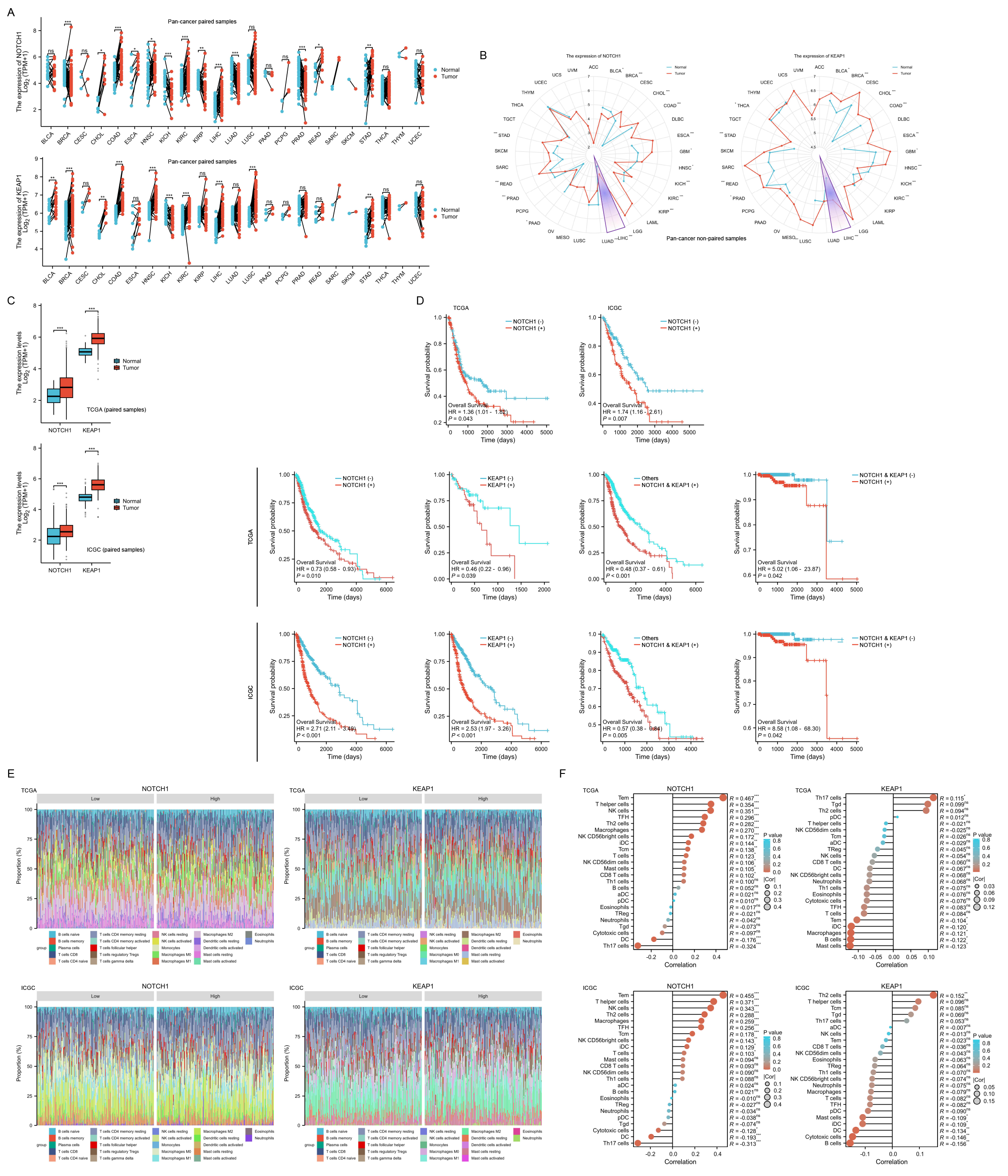


**Supplementary figure 9. NOTCH1 and KEAP1 expression are synergistic with liver cancer.** (**A**) Pan-cancer analysis showing the abnormal increased expression of NOTCH1 and KEAP1 in different tumor pathology. (**B**) Pan-cancer analysis in non-paired samples showing the abnormal increased expression of NOTCH1 and KEAP1 in different tumor pathology. (**C**) The expression of NOTCH1 and KEAP1 in patients with liver tumor based on TCGA and ICGC database. (**D**) Kaplan-Meier curves showing the survival analysis based on TCGA HCC and ICGC HCC prognostic data for NOTCH1-positive, KEAP1-positive, and NOTCH1 & KEAP1 co-positive patients. (**E, F**) Density heatmap and lollipop chart showing the NOTCH1- and KEAP1-positive expression-related immune infiltration based on TCGA and ICGC database.


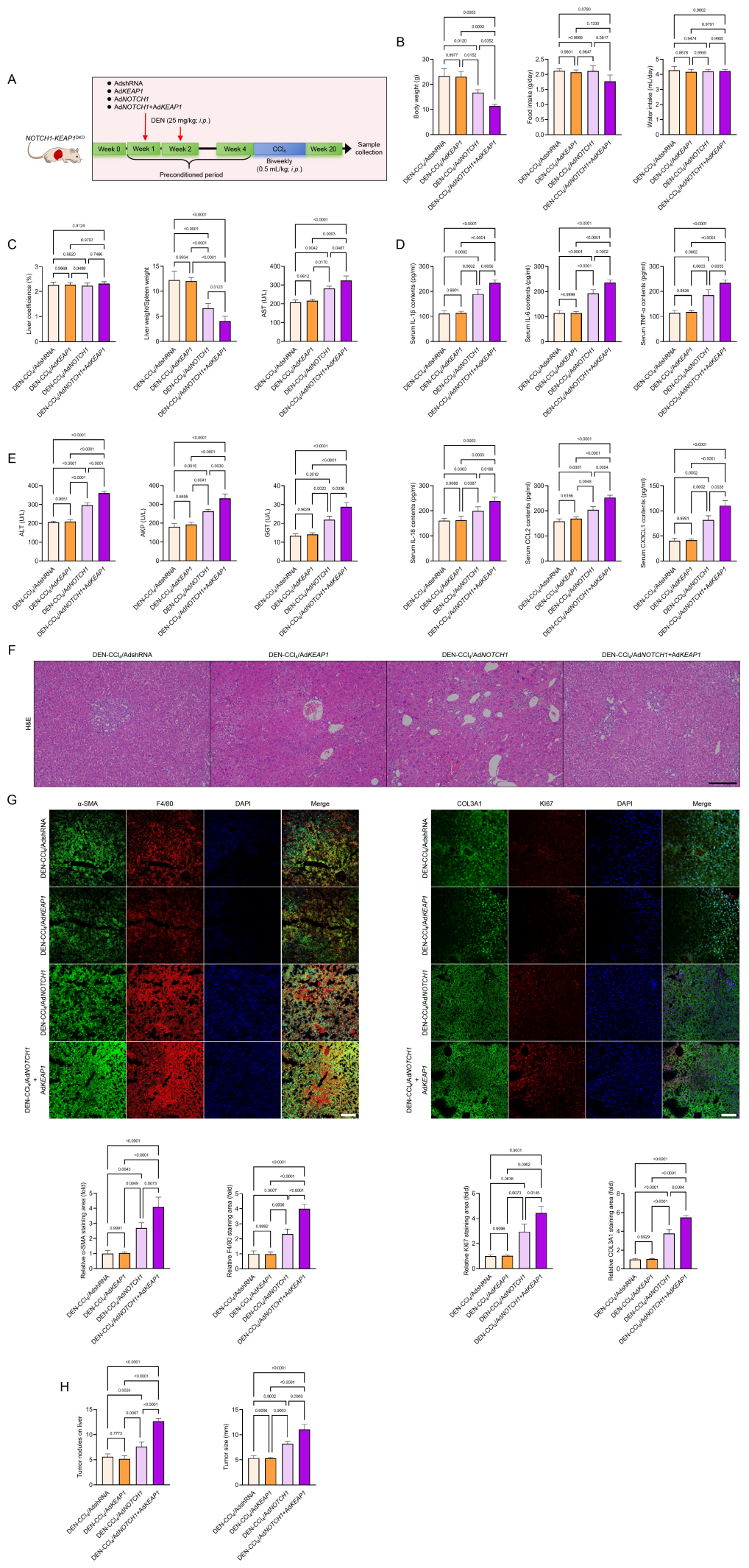


**Supplementary figure 10. KEAP1 is required for NOTCH1 function-mediated hepatocellular carcinoma (HCC) progression in *NOTCH1* and *KEAP1* dual-deficient mouse.** (**A**) Schematic diagram for Ad*NOTCH1*-, Ad*KEAP1*-, and Ad*NOTCH1*+Ad*KEAP1*-injected *NOTCH1* and *KEAP1* dual-deficient mouse with DEN+CCl_4_-induced HCC progression *in vivo* (*n*=10 per group). (**B-E**) Records of body weight, food intake, water intake, liver coefficience, liver-to-spleen ratio, serum AST, ALT, AKP & GGT contents, and serum pro-inflammatory cytokines IL-1β, IL-6, TNF-α, IL-18, CCL2 and CX3CL1 levels in the indicated groups (*n*=10 per group). (**F**) Representative histological analysis of liver sections detected by H&E staining in the indicated groups (magnification, 100×, *n*=10 samples). (**G**) Representative immunofluorescence analysis of liver samples showing the F4/80 and α-SMA co-expression, and COL3A1 and KI67 co-expression in the indicated groups, respectively (magnification, 100×, *n*=10 samples). (**H**) Measurement of tumor nodules on liver and tumor size for the indicated groups (*n*=10 samples). Data are presented as mean ± SEM. The associated experiments were performed independently at least three times. *P* <0.05 indicates statistical significance.


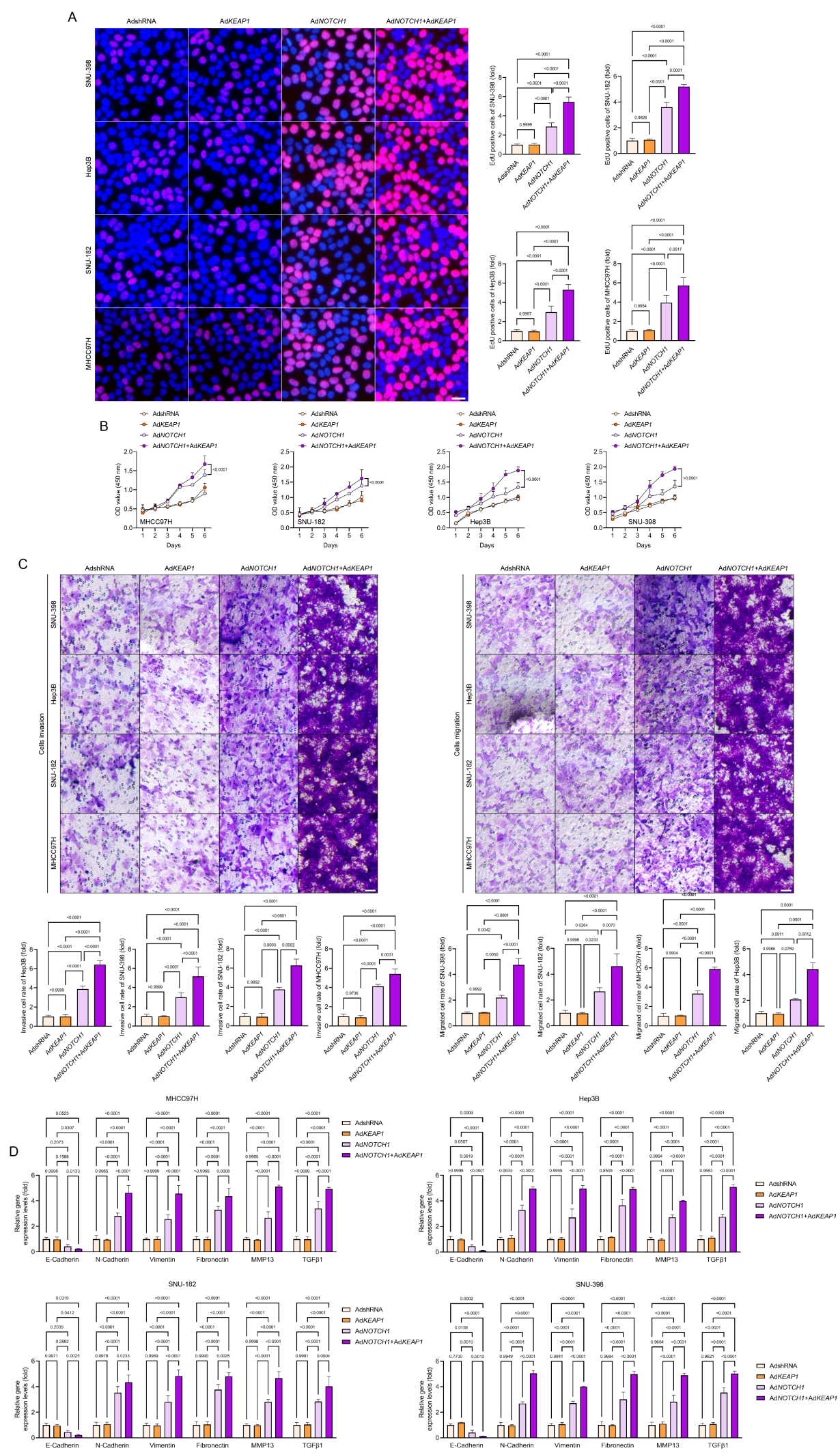


**Supplementary figure 11. Effects of NOTCH1-KEAP1 co-expression on EMT and metastasis *in vitro*.** Indicated NOTCH1-KEAP1 dual-deleted HCC cell lines (SNU-398, Hep3B, SNU-182 and MHCC97H) were transfected with AdshRNA, Ad*KEAP1*, Ad*NOTCH1* and Ad*NOTCH1*+Ad*KEAP1*, respectively. Subsequently, all cells were collected for further analysis as follows. (**A**) EdU analysis was used to assess the proliferation of HCC cells; and quantification for the percentage of EdU analysis for the indicated groups (*n*=10 samples). (**B**) CCK-8 detection was performed to evaluate the impact of NOTCH1 and KEAP1 on HCC cell growth (*n*=10 samples). (**C**) Transwell assay was performed to investigate cell migration and invasion in different HCC cell lines following transfection with AdshRNA, Ad*KEAP1*, Ad*NOTCH1* and Ad*NOTCH1*+Ad*KEAP1*, respectively (magnification, 100×, *n*=10 samples). (**D**) EMT-associated genes in HCC cells were analyzed using qPCR after manipulating AdshRNA, Ad*KEAP1*, Ad*NOTCH1* and Ad*NOTCH1*+Ad*KEAP1* transfection, respectively (*n*=10 samples). Data are presented as mean ± SEM. The associated experiments were performed independently at least three times. *P* <0.05 indicates statistical significance.
