# Supplementary material for: Targeting NOTCH1-KEAP1 axis retards chronic liver injury and liver cancer progression via regulating stabilization of NRF2
Source: J Exp Clin Cancer Res. 2025 Aug 9;44:232. doi: 10.1186/s13046-025-03488-3 (PMC12335071; doi:10.1186/s13046-025-03488-3)
Supplement: Supplementary file 2 — Supplementary Material 2 [file 13046_2025_3488_MOESM2_ESM.docx]

**Supplementary table 1. Antibodies information used in this work.**

| **Name** | **Dilution** | **Catalog number** | **Company** |
| --- | --- | --- | --- |
| GAPDH | 1/1500 | ab9485 | Abcam |
| NF-κB/p65 | 1/1000 | ab32536 | Abcam |
| p-NF-κB/p65 | 1/1000 | ab76302 | Abcam |
| 4-HNE | 1/200 | ab48506 | Abcam |
| F4/80 | 1/150 | ab16911 | Abcam |
| NOTCH1 | 1/150 | MA5-11961 | Thermo Fisher Scientific |
| NOTCH1 | 1/1000 | MA5-32080 | Thermo Fisher Scientific |
| SLC7A11 | 1/1000 | PA1-16893 | Thermo Fisher Scientific |
| Nrf2 | 1/1000 | PA5-27882 | Thermo Fisher Scientific |
| KEAP1 | 1/150-1/1000 | PA5-99434 | Thermo Fisher Scientific |
| α-SMA | 1/100 | 14-9760-82 | Thermo Fisher Scientific |
| Hes1 | 1/100-1/500 | sc-166410 | Santa Cruz Biotechnology |
| ACSL4 | 1/500 | sc-271800 | Santa Cruz Biotechnology |
| NICD1 | 1/200-1/1000 | 4147 | Cell Signaling Technology |
| Ub | 1/1000 | GB115700 | Servicebio |
| COL1A1 | 1/200 | GB11022 | Servicebio |
| GPX4 | 1/100-1/1000 | GB124327 | Servicebio |
| Flag | 1/1000 | 14793 | Cell Signaling Technology |
| HA | 1/1000 | ab9110 | Abcam |
| His | 1/1000 | D291-3 | MBL Beijing Biotech |
| HNF4A | 1/200-1/1000 | ab201460 | Abcam |
| α-Tubulin | 1/1000 | ab7291 | Abcam |
| Lamin B | 1/1000 | ab16048 | Abcam |
| F4/80 | 1/1000 | ab300421 | Abcam |
| F4/80 | 1/1000 | ab254293 | Abcam |
| Hes1 | 1/200-1/1000 | ab108937 | Abcam |
| α-SMA | 1/1000 | ab7817 | Abcam |
| Collagen 1 | 1/1000 | ab270993 | Abcam |
| 4-HNE | 1/200-1/1000 | MA5-27570 | Thermo Fisher Scientific |
| Ub-K48 | 1/1000 | MA5-35382 | Thermo Fisher Scientific |
| Anti-Mouse IgG (H&L) | 1/5000-1/10000 | ab6728 | Abcam |
| Anti-Rabbit IgG (H&L) | 1/5000-1/10000 | ab6721 | Abcam |

**Supplementary table 2. Primers for real-time RT-PCR.**

| **Items** | **Forward (5’ to 3’)** | **Reverse (5’ to 3’)** |
| --- | --- | --- |
| *Homo* NOTCH1 | GTGAGACCTGCCTGAATGG | GTTGGGGTCCTGGCATC |
| *Homo* GAPDH | GGAGCGAGATCCCTCCAAAAT | GGCTGTTGTCATACTTCTCATGG |
| *Homo* HO-1 | CTGGAGGAGGAGATTGAGCG | ATGGCTGGTGTGTAGGGGAT |
| *Homo* NQO1 | TGCAGCGGCTTTGAAGAAGAAAGG | TCGGCAGGATACTGAAAGTTCGCA |
| *Homo* GCLC | GAGGTCAAACCCAACCCAGT | AAGGTACTGAAGCGAGGGTG |
| *Homo* SOD1 | GACTGACTGAAGGCCTGCAT | TGATGGACGTGGAACCCATG |
| *Homo* GPX4 | CTCCATGCACCAGTTTCC | AGGTCGACGAGCTGAGTGT |
| *Homo* SLC7A11 | CCCAGATATGCATCGTCCTT | CCTGGGTTTCTTGTCCCATA |
| *Homo* TNF-α | CCTCTCTCTAATCAGCCCTCTG | GAGGACCTGGGAGTAGATGAG |
| *Homo* IL-1β | CCTGCGTGTTGAAAGATGATAA | CTGCTTGAGAGGTGCTGATGTA |
| *Homo* IL-6 | CCAGGAGCCCAGCTATGAAC | CCCAGGGAGAAGGCAACTG |
| *Homo* CCL2 | GTCTCTGCCGCCCTTCTG | ACTTGCTGCTGGTGATTCTTCT |
| *Homo* KEAP1 | CTGGAGGATCATACCAAGCAGG | GGATACCCTCAATGGACACCAC |
| *Mus* HO-1 | GGGTGATAGAAGAGGCCAAGA | AGCTCCTGCAACTCCTCAAA |
| *Mus* NQO1 | AGAAGAGCACTGATCGTACTGG | CGTAATTGTAAGCAAACTCTCCTATG |
| *Mus* GCLC | ACTAGGCTGTCCTGGATTCA | TTGCCCATCCCGAATCCCA |
| *Mus* SOD1 | GAGACCTGGGCAATGTGACT | GTTTACTGCGCAATCCCAAT |
| *Mus* GPX4 | TGTGCATCCCGCGATGATT | CCCTGTACTTATCCAGGCAGA |
| *Mus* SLC7A11 | GATGCTGTGCTTGGTCTTGA | GCCTACCATGAGCAGCTTTC |
| *Mus* SLC3A2 | TGTACTTGGCTGAGTGGCAG | AGGTCGCTGGTGGATTCAAG |
| *Mus* TNF-α | ACCTGGCCTCTCTACCTTGT | CCCGTAGGGCGATTACAGTC |
| *Mus* IL-1β | GCCACCTTTTGACAGTGATGAG | AGTGATACTGCCTGCCTGAAG |
| *Mus* IL-6 | GGGCAACAAGATTCCGATATA | AGCCACTACATGGAATCTAAT |
| *Mus* CCL2 | GGCTCAGCCAGATGCAGTTAA | CCTACTCATTGGGATCATCTTGCT |
| *Mus* COL3A1 | CCCAACCCAGAGATCCCATT | GAAGCACAGGAGCAGGTGTAGA |
| *Mus* COL1A1 | CGATGGATTCCCGTTCGAGT | AAGGGTGCTGTAGGTGAAGC |
| *Mus* α-SMA | TTCCTTCGTGACTACTGCCG | TATAGGTGGTTTCGTGGATGCC |
| *Mus* GAPDH | GGTGAAGGTCGGTGTGAACG | CCCGTAGGGCGATTACAGTC |
